# Publication guidelines for human heart rate and heart rate variability studies in psychophysiology—Part 1: Physiological underpinnings and foundations of measurement

**DOI:** 10.1111/psyp.14604

**Published:** 2024-06-14

**Authors:** Karen S. Quigley, Peter J. Gianaros, Greg J. Norman, J. Richard Jennings, Gary G. Berntson, Eco J. C. de Geus

**Affiliations:** 1 Department of Psychology, Northeastern University, Boston, Massachusetts, USA; 2 Department of Psychology, University of Pittsburgh, Pittsburgh, Pennsylvania, USA; 3 Department of Psychology, The University of Chicago, Chicago, Illinois, USA; 4 Department of Psychiatry & Psychology, University of Pittsburgh, Pittsburgh, Pennsylvania, USA; 5 Department of Psychology & Psychiatry, The Ohio State University, Columbus, Ohio, USA; 6 Department of Biological Psychology, Vrije Universiteit Amsterdam, Amsterdam, the Netherlands

**Keywords:** guidelines, heart rate, heart rate variability, methodology, respiratory sinus arrhythmia, statistics, study design

## Abstract

This *Committee Report* provides methodological, interpretive, and reporting guidance for researchers who use measures of heart rate (HR) and heart rate variability (HRV) in psychophysiological research. We provide brief summaries of best practices in measuring HR and HRV via electrocardiographic and photoplethysmographic signals in laboratory, field (ambulatory), and brain-imaging contexts to address research questions incorporating measures of HR and HRV. The *Report* emphasizes evidence for the strengths and weaknesses of different recording and derivation methods for measures of HR and HRV. Along with this guidance, the *Report* reviews what is known about the origin of the heartbeat and its neural control, including factors that produce and influence HRV metrics. The *Report* concludes with checklists to guide authors in study design and analysis considerations, as well as guidance on the reporting of key methodological details and characteristics of the samples under study. It is expected that rigorous and transparent recording and reporting of HR and HRV measures will strengthen inferences across the many applications of these metrics in psychophysiology. The prior Committee Reports on HR and HRV are several decades old. Since their appearance, technologies for human cardiac and vascular monitoring in laboratory and daily life (i.e., ambulatory) contexts have greatly expanded. This *Committee Report* was prepared for the Society for Psychophysiological Research to provide updated methodological and interpretive guidance, as well as to summarize best practices for reporting HR and HRV studies in humans.

## 1 | INTRODUCTION

Decades have passed since two *Committee Reports* were prepared for the Society for Psychophysiological Research on heart rate (HR; Jennings et al., 1981) and HR variability (HRV; Berntson et al., 1997). These *Committee Reports* still provide psychophysiologists with rich and detailed information on how to measure, process, analyze, interpret, and report measures of HR and HRV. Since their appearance, however, technologies for human cardiac and vascular monitoring in laboratory and field (i.e., daily life or ambulatory) contexts have expanded. This growth has coincided with ever-expanding analytic approaches, new measures, and greater knowledge of the neurophysiology of HR and HRV, and their interrelationships, all of which can guide more precise inferences about these chronotropic (rate) phenomena. In view of these developments, this updated *Committee Report* was prepared for the Society for Psychophysiological Research to provide researchers with methodological and interpretive guidance, as well as to summarize best practices for reporting HR and HRV studies in humans.

This *Committee Report* does not cover prominent theories and conceptual frameworks pertaining to HR and HRV, applications of HR and HRV in clinical and developmental psychology, cardiology, or studies of biofeedback, interpersonal synchrony, or interoception. Rather, it is intended to aid in replicable recording of HR and HRV, their careful interpretation, and to enhance transparent reporting on these parameters for all applications. To these ends, this *Committee Report*: (1) summarizes common measures of HR and HRV, as well as available recording methods suitable for laboratory and ambulatory (or other field) contexts; (2) reviews and compares conventional and emerging measures and associated analytic approaches; (3) considers specific issues arising from the use of HR and HRV in ambulatory and brain-imaging studies; and (4) describes what is known about the physiology of the heart and its intrinsic and extrinsic control. The *Committee Report* concludes with guidance on key elements of study design, data collection, data processing, and data analytic approaches, as well as guidance on the reporting of methodological details and characteristics of the samples under study. We also refer readers to a forthcoming companion *Committee Report* that will provide a detailed treatment of contextual, lifespan, and other person-level factors relevant to understanding HR and HRV in psychophysiological research.

Because some readers may be novices and others more seasoned in HR and HRV research, we utilize didactic figures and boxes to provide more in-depth information where applicable. A Glossary of terms is provided near the end of this *Report*. For readers interested in additional resources to supplement our coverage, we recommend reviews of cardiac neurophysiology and function (Baruscotti et al., 2010; Dobrzynski et al., 2013; Hall & Hall, 2021a; Kleber & Rudy, 2004; Mangoni & Nargeot, 2008; Monfredi et al., 2013; Palma & Benarroch, 2014; van Weerd & Christoffels, 2016), overviews of cardiac recording methods (Hall & Hall, 2021b; Kranjec et al., 2014), reviews of HRV measures (Acharya et al., 2006; Allen et al., 2007; Bravi et al., 2011; Sassi et al., 2015; Shaffer & Ginsberg, 2017; Sztajzel, 2004; Task Force of the European Society of Cardiology and the North American Society of Pacing and Electrophysiology, 1996; Xhyheri et al., 2012), and reviews of HRV and detailed treatments of its physiological bases (Acharya et al., 2006; Berntson et al., 1993b; Malik & Camm, 1995; Quintana & Heathers, 2014; Task Force of the European Society of Cardiology and the North American Society of Pacing and Electrophysiology, 1996). For historical coverage of HR, we refer readers to a review by Ghasemzadeh and Zafari (2011) and for HRV, a review by Billman (2011). Lastly, for readers interested in the relationship between the prevailing HR and concurrent HRV, we recommend a review that provides complementary reporting guidance to this *Committee Report* (de Geus et al., 2019).

The initial part of the *Committee Report* is organized to follow the development of a research project and writing of a research report, with measures described first, followed by their collection and associated best practices, and then analytic issues. Next, we describe, in brief, environmental and individual differences issues related to HR and HRV. Finally, we provide a description of the underlying physiological origins of these measures. Our recommendation is that before choosing measures, researchers carefully consider the underlying physiology, which necessarily constrains the inferences that can be made from these measures.

## 2 | HEART RHYTHM AND THE ELECTROCARDIOGRAM (ECG)

**FIGURE 1:**
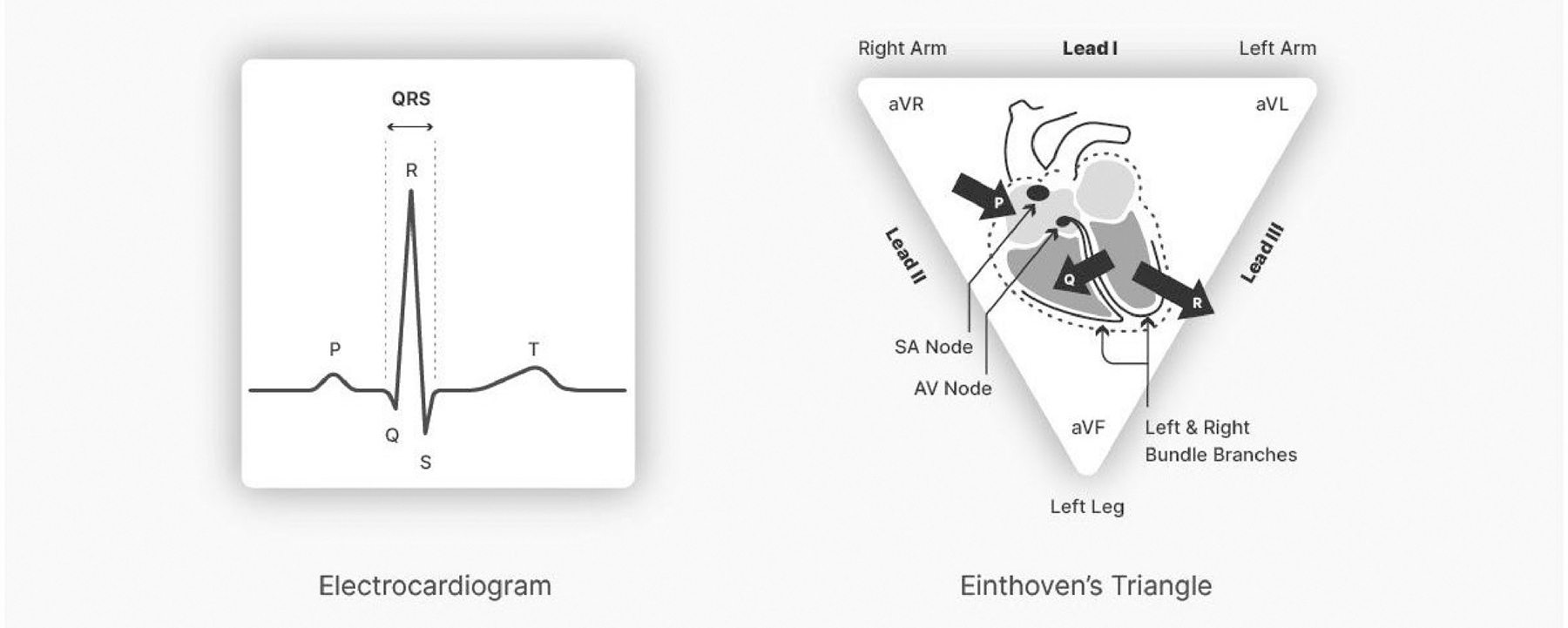
Electrocardiogram (ECG) and Einthoven’s triangle. The left panel shows the electrocardiogram (ECG) with waves P, Q, R, S, and T. The right diagram places the heart within Einthoven’s triangle with light gray shading in the atria and dark gray shading in the ventricles. The heart is depicted, by convention, as if one is looking into the chest from outside the body, hence the left heart is on the right side of the picture. Here, for clarity we show only the predominant electrical vector for the P, Q, and R waves.

Heart rate (HR) is the main index of the heart rhythm for a psychophysiologist. The typical measurement of HR is based on electrical events generated by the heart during its normal function. The ECG comprises a series of voltage deflections, each corresponding to electromechanical events in the heart and designated with the letters P through T (see Figure 1).

The P wave corresponds to the electrical depolarization of the left atrium, which leads to contraction of both atria. The P wave is followed by the QRS complex, which reflects voltage conduction across the heart, and the electrical depolarization of the ventricles that initiates contraction. This contraction forces blood into the pulmonary artery (from the right heart) and aorta (from the left heart). The QRS complex is followed by the T wave, which reflects repolarization of the ventricles. The time between any specific voltage deflection in the ECG across two consecutive heartbeats (e.g., between successive R waves) is referred to as the heart period (HP), which is synonymous with the terms, interbeat interval (IBI) and R–R interval. We will employ HP throughout most of this report to indicate the time between successive heartbeats.

### 2.1 | Quantification of HR

HR is typically considered to be the number of HPs in a 60-s period (and expressed in beats per minute or bpm). Thus, HR is the inverse of HP. However, it is important to keep in mind that the transformation of HP to HR is not linear, with HR (bpm) = 60,000/HP (ms). The reverse transformation is also not linear, with HP (ms) = 60,000/HR (bpm). Because HR and HP reflect the timing of cardiac phenomena, both are chronotropic (rate) measures. Although HR is most often employed as the basic metric in studies of cardiac function and cardiac rhythms, its nonlinear transformation from HP requires an explicit rationale. A consistent finding is that the function relating the frequency of vagal activation to HR is hyperbolic, whereas the relationship to HP is relatively linear, at least over typical operating ranges (Dexter et al., 1989; Parker et al., 1984; for reviews, see Berntson et al., 1995; de Geus et al., 2019; Eckberg & Sleight, 1992). Furthermore, within a moderate range of sympathetic activation, autonomic interaction effects between the sympathetic and parasympathetic branches are notable when indexed by HR but considerably less when indexed by HP (Quigley & Berntson, 1996).^1^ HP also typically shows a statistically more normal distribution than HR (Jennings et al., 1974). HP is therefore often the more appropriate metric to use in scientific analyses when inferring neural influences.

Beyond the issues related to autonomic influence over cardiac chronotropy, Graham (1978) cautioned that HR per second (in bpm units) and HP per beat (in ms units) are the only measures that can be used to correctly estimate common parameters such as the arithmetic mean. In addition, Porges et al. (1980) proposed a weighted HP measure that enables calculation of HP for any desired epoch. Failure to weight the HP by the duration of the epoch will have especially notable effects when epochs of interest are short, where the effect of the lack of weighting for partial beats can be substantial. Like HR per second, the weighted HP metric is independent of the duration of the epoch (See Box 1 for the calculation) (Figure 2).

### 2.2 | Resting HP and task-induced HP reactivity

Although resting HR is a commonly used term, based on the above here we discuss the determinants of its inverse, resting HP, to take advantage of the more linear relationship between autonomic inputs and HP. To succinctly summarize the main determinants of the resting HP, the formula in (1) can be used (see Berntson et al., 1993a):

(1) HP=intrinsicHP+ΔHP_SNS+ΔHP_PNS+ΔHP[SNS×PNS]+ε

where intrinsic HP (iHP) reflects the HP of the denervated heart, which can be assessed by dual pharmacological blockade of the cardiac sympathetic nervous system (SNS) and parasympathetic nervous system (PNS) effects. The intrinsic HP is an important determinant of individual differences in the observed resting HP. The intrinsic HP is relatively fixed over short time spans but can change in the longer term as seen with aging when intrinsic HPs are longer (Peters et al., 2020) as they are after prolonged exercise training (Boyett et al., 2017). The intrinsic HR (iHR) is simply the inverse (60,000/iHP).

ΔHP_SNS is the decrease in HP below the intrinsic HP caused by sympathetic activation, ΔHP_PNS is the increase in HP above the intrinsic HP caused by parasympathetic activation, and ΔHP_SNS×PNS is the interaction term, which derives from “accentuated antagonism” at the neurocardiac junction (Mizuno et al., 2008), and also mutual modulation of the SNS and PNS by higher brain systems (Rajendran et al., 2019). Of note, what appears as “accentuated antagonism” when using HR is less prominent when using HP (due to nonlinearities in the transform of HR into HP; Quigley & Berntson, 1996). Finally, the error term (ε) subsumes all humoral and intrinsic chronotropic effects plus any phasic variations around the average (or tonic) resting HP level described by the formula. Thus, a typical 40-year old at rest with an intrinsic HP of 600 ms (iHR = 100 bpm), a small cardiac sympathetic effect of ΔHP_SNS of −35 ms (+6 bpm), a moderate (and typical) cardiac parasympathetic ΔHP_PNS of +280 ms (−32 bpm), and no noticeable SNS × PNS interaction, has an average resting HP of 845 ms (71 bpm).

When the experimental focus is on short-term HR changes induced by physical or psychological manipulations, that is, “HR reactivity,” changes in intrinsic HP are not likely to play a role. Such changes in chronotropy are predominantly determined by changes in the activity of the two branches of the ANS. Often these changes have been erroneously assumed to act solely in a reciprocal manner, with increases in SNS activity paired to decreases in PNS activity. The origins of this dogma of strict reciprocal activation of the SNS and inhibition of the PNS (and vice versa) can be attributed most prominently to Walter Cannon and his contemporaries (Cannon, 1914, 1915; Fulton, 1949), and has been promulgated by Malliani and colleagues in relation to the construct of “sympathovagal balance” (Malliani et al., 1998). In contrast, substantial evidence shows that cardiac parasympathetic and sympathetic activity can be independent, circumscribing a two-dimensional space rather than occupying a single axis of reciprocally opposed sympathetic/parasympathetic changes (Berntson et al., 1995, 1991, 1993a; Berntson, Cacioppo, Binkley, Uchino, et al., 1994; Berntson, Cacioppo, & Quigley, 1994; Berntson, Cacioppo, Quigley, & Fabro, 1994; Cacioppo et al., 1994).

**FIGURE 3:**
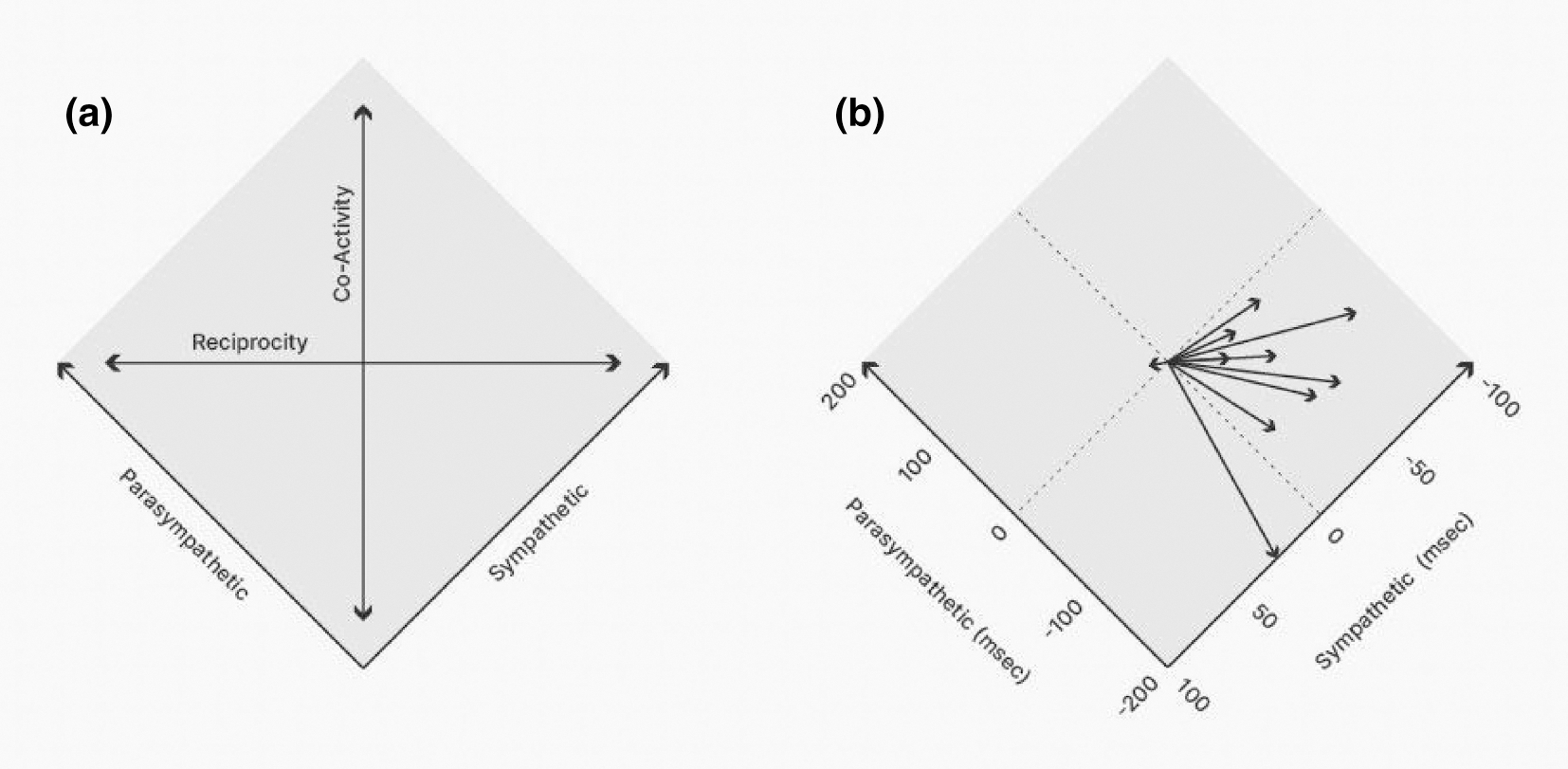
Patterns of reciprocal and coactivational change in sympathetic nervous system (SNS) and parasympathetic nervous system (PNS) activity within the Autonomic Space. Panel (a) shows the conceptual model of Autonomic Space, illustrating reciprocal and coactivational modes of change. Panel (b) shows mean responses (changes scores from the 0, 0 center point) for *n* = 10 different individuals to three stimuli (reaction time task, math task and speech task) within this space, in milliseconds of change in heart period (HP) (data from Figure 4 in Berntson, Cacioppo, Binkley, Uchino, et al., 1994).

As illustrated in Figure 3, we again express sympathetic and parasympathetic activity as a change in HP rather than HR and see that changes in HP are a functional outcome that results from the combined activity of the sympathetic and parasympathetic autonomic branches. Activity in these two branches indeed can be functionally antagonistic, a “Cannonical” reciprocal activation of SNS and deactivation of PNS or vice versa (with movement in autonomic space along the horizontal axis shown in Figure 3). This is an oft-observed pattern, for instance, in prototypical psychological stressor tasks common to psychophysiological research (Berntson, Cacioppo, Binkley, Uchino, et al., 1994; Brindle et al., 2014). However, activity in the two autonomic branches also can exhibit coactivation and coinhibition (along the vertical co-activity axis in Figure 3; e.g., see Gianaros & Quigley, 2001), leading to substantial heterogeneity across individuals in response to the same stimulus, even when HP changes are comparable (Berntson, Cacioppo, Binkley, Uchino, et al., 1994). This means that a given HP change can be achieved by multiple different combinations of change in sympathetic and parasympathetic activity. This is an example of a broader, common biological phenomenon known as degeneracy, whereby multiple patterns of biological activity or change can result in the same functional outcome (Edelman & Gally, 2001; Leonardo, 2005; Whitacre & Bender, 2010).

The concept of autonomic space has important implications for interpreting changes in HP across experimental conditions and daily life contexts. Namely, a change in HP alone cannot disambiguate the underlying sympathetic and parasympathetic contributions to that chronotropic change. This further emphasizes that if one wants to make inferences about the underlying autonomic contributions to a chronotropic metric, one needs to measure (or at minimum, estimate) those contributions independently (e.g., Berntson et al., 2008; Gianaros & Quigley, 2001).

## 3 | SIGNAL ACQUISITION, PREPROCESSING, AND QUANTIFICATION OF THE HP TIME SERIES

### 3.1 | Signal acquisition

#### 3.1.1 | Electrocardiographic recordings

The cardiac signal can be recorded with the highest fidelity via the ECG. The hardware and software of many current commercial data acquisition systems result in excellent ECG signal quality. The ECG is a voltage signal, which is acquired most commonly via electrodes made of an amalgam of silver and silver chloride (Ag/AgCl) and using a high-conductivity gel (generally 3–10% sodium chloride (NaCl)). Electrodes are affixed after skin preparation with alcohol and/or light skin abrasion to remove dead skin cells and oils to reduce resistance at the electrode-skin interface and enhance signal quality.

**FIGURE 4:**
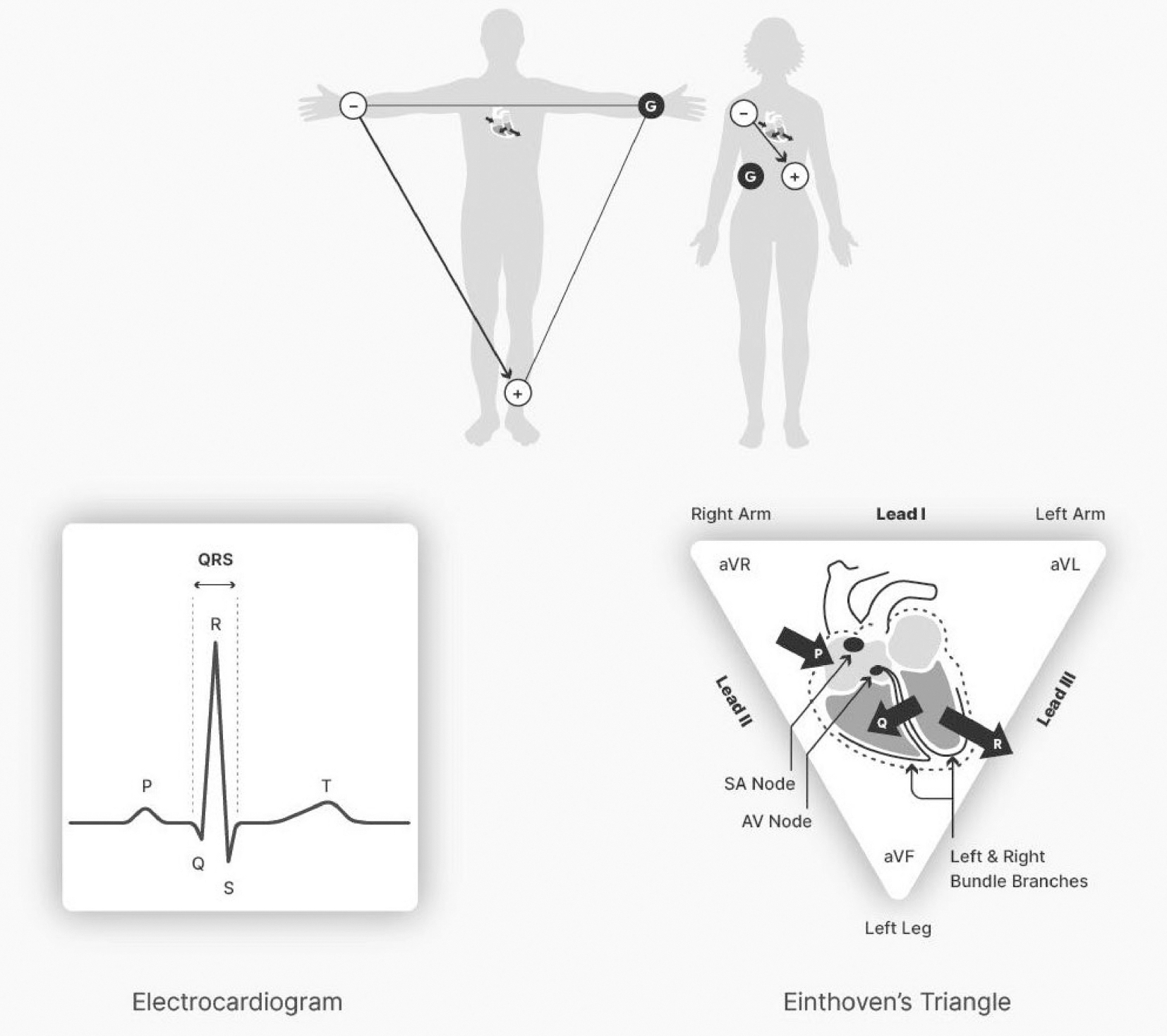
Lead II (top left) and modified Lead II (top right) electrocardiogram (ECG) placements. The top left figure illustrates the electrode placements for ground (G) as well as negative (−) and positive (+) electrodes corresponding to Einthoven’s scheme. The top right figure illustrates a commonly used modified Lead II placement in which the ground (G or reference) is placed on the lower right thorax, rather than on the arm to minimize movement artifact. These lead configurations are applicable to both females and males. The bottom panels illustrate the ECG and the heart within Einthoven’s triangle.

Electrode placements should be specified anatomically. For many psychophysiological applications, electrode placements are used that result in a prominent R wave relative to other waves in the ECG. Common placements include either a Lead II (e.g., active leads on the left leg and right arm, reference on the right leg) or a modified Lead II configuration (i.e., active leads on a lower left rib and right collarbone, reference on a lower right rib). The modified Lead II (torso) placement minimizes movement artifact that can occur with limb leads. This configuration permits electrode placement without disrobing if garments that open at the waist are worn. In a wired configuration, movement artifacts can be reduced further by taping down a loop of electrode lead, to provide strain relief so that there is no tension on the lead wire. Comfortable positioning of the participant is also advisable to minimize fidgeting and postural adjustments. Figure 4 (top) shows electrode placements for limb-based (top left) and modified (top right) Lead II placements with a typical modified Lead II-derived ECG waveform (bottom left) and Einthoven’s triangle (bottom right).

Deriving a cardiac chronotropic metric from an ECG signal, whether HP or its inverse, HR, requires reliable identification of a specific fiducial point in the ECG, most typically the peak of the R wave of the QRS complex. The HP also can be derived from other peaks in the ECG, for example, in medical applications or when other features of the ECG waveform are of interest (Takahashi et al., 2016). When the HP is derived from a waveform feature other than the R wave, for example, from the P wave onset, even greater methodological care is required as these waveform features are harder to detect with high precision.

The analog ECG signal is typically sampled at 250–1000 Hz for measurement of HP, converted to digital form, and stored via a computer-based data acquisition system. Modern laboratory-based data acquisition systems impose few limitations on the sampling rate, and higher sampling rates will reduce error in detecting the R wave (e.g., at 1000 Hz, the R-wave can be detected with ±1 ms accuracy, whereas 250 Hz would reduce accuracy to ± 4 ms). 1000 Hz is therefore the recommended frequency, particularly when the R wave is linked to parallel events as in cardiac cycle time effects (Grund et al., 2022), cardiac monitoring during brain imaging (Gray, Rylander, et al., 2009), or ensemble averaging in impedance cardiography (de Geus & Gevonden, 2023; Hoemann et al., 2020). In other contexts, where the researcher may need to balance the need for high sampling rates against limited data storage capacity or device limitations (Riniolo & Porges, 1997) and where averaging is done across many beats, lower sampling frequencies are feasible. This may include prolonged recording with ambulatory devices where the availability of a very large data set with many repeated measures might offset the measurement error induced by the lower sampling frequency. However, researchers must recognize the inherently higher error introduced by this choice and consider its potential impact, especially for HRV metrics.

#### 3.1.2 | Photoplethysmographic recordings

A photoplethysmographic (PPG) signal is a light-derived signal obtained by illuminating the skin, typically via an infrared emitter and a light-sensitive phototransistor as the receiver (Allen et al., 2007; Jennings et al., 1980; Lee et al., 2021). Typically, both emitter and receiver are contained within the same small housing unit and affixed to the skin. In some cases, the emitting source may be opposite to the receiver (e.g., on opposite sides of a finger or earlobe), but more typically, the two are adjacent to one another. Blood flow through the area is measured by how absorption, scattering, and reflectance of the infrared light activates the receiver. With each heartbeat, blood flow within the vasculature produces a beat-by-beat blood volume pulse (BVP) signal. The BVP signal is dependent on both blood flow and pressure. A HP time series can be derived from the PPG signal or from a continuous blood pressure signal recorded via means other than the PPG. An increasingly common light-based method, pulse oximetry, produces a signal that has the same visual appearance as a PPG signal, but this method uses different colors of light stimulation to assess blood oxygenation under the sensor. By comparison, the PPG is more suitable for HR or HRV measurement than pulse oximetry. These signals are commonly used to estimate HP and HRV in ambulatory settings or during magnetic resonance imaging of the brain. For a more detailed discussion of PPG measures, we refer readers to Lee et al. (2021) and Allen (2007).

The ease of use and noninvasiveness of PPG should be weighed against several factors, including noise sources and variations in specifications across devices (Fine et al., 2021). In these regards, the PPG method has a much lower signal-to-noise ratio than the ECG, and this difference will be amplified by movement and physical activity (Georgiou et al., 2018; Nelson et al., 2020; Yuda et al., 2020). Second, accurate detection of a peak from the PPG output is usually more difficult than detecting an R wave on the ECG because PPG signals lack sharp peaks. Even with good template-matching approaches, accurate fiducial-point identification for this waveform can be problematic. This is particularly true for some placements of the PPG, for example, on the ear lobe, but also true for commonly measured sites such as a finger or wrist. Third, individual differences in skin color, skin thickness, and adiposity can impact PPG signals (Fine et al., 2021). Tissue composition and thickness, as well as pressure on the skin, will alter PPG morphology and amplitudes, creating problems both for between-participant and repeated (or between-session) studies. PPG devices using infrared stimulation frequencies (typically in 800–950 nm wavelength range) are minimally sensitive to skin color, but green-light stimulation, which is often used in ambulatory devices will be more affected by skin color, and may affect reliability, although the effects depend upon the device and the level of physical activity during measurement (Bent et al., 2020). Placement over tattooed skin is not advised.

Finally, propagation of the BVP is influenced by peripheral vascular changes that can alter the shape of the blood pressure waveform from beat to beat. The ejection of blood from the heart is itself partially a function of both pressure and characteristics of the aorta and other arterial vessels, so as the pulse travels away from the heart, vascular characteristics transform BVP waveform morphology. Hence, peak timing and pulse shape can be influenced by factors such as changes in compliance in the aorta, artery size, vasoconstriction or dilation due to thermoregulation or other factors, changes in viscosity of the blood, or changes in overall blood volume in the perfused bed (Yuda et al., 2020). Acute vasoconstriction or vasodilation related to psychological or physiological stimuli also alters the BVP. Different peak timing and pulse shape will be observed at different locations, which can reveal useful information about arterial stiffening; it also means that PPG-derived HP measures differ across recording sites, for example, ear versus wrist. Group comparisons using PPG-based measures are made difficult by between-subject differences in arterial compliance, such as vessel stiffening with age or structural changes like stenosis. All of these considerations mean that two equivalent R–R intervals derived from R-wave timing from the ECG can be associated with different pulse-to-pulse intervals derived from the PPG.

Good to acceptable correspondence between HP derived from simultaneous PPG and ECG recordings under resting conditions typically deteriorates with increasing physical activity (Bent et al., 2020; Nelson et al., 2020). A comparison of commercial-grade PPG-based beat detection to ECG-based detection under resting and physically active conditions yields greater variation with activity (vs. rest) and considerable variation across devices (Bent et al., 2020; Dooley et al., 2017; Jo et al., 2016; Stahl et al., 2016); also see review by Schäfer (Schäfer & Vagedes, 2013). There has been increasing interest in video-based measures of HP (Kranjec et al., 2014; Poh, McDuff, & Picard, 2010; Sun & Thakor, 2015) that rely on the peripheral pulse detected from blood flow-associated color changes or subtle bodily movements in the face or other body parts. However, the same cautions about the accuracy of beat detection timing apply as for PPG and other vascular-based measures.

Regardless of the nature of the hardware, the accuracy of any newly introduced recording system should be evaluated against a recording system that utilizes a known (not proprietary) and tested technology (i.e., considered a gold standard) or against simulated calibration signals with known characteristics, for example, from Physionet (Vest et al., 2018) or NeuroKit2 (Makowski et al., 2021; Pham et al., 2021).

### 3.2 | Preprocessing

The foundation for measures of cardiac chronotropy (i.e., HP and HR) rests upon the integrity of the input signal (e.g., ECG or PPG) and the accuracy of the detection of the point in each cardiac cycle from which measurements are made, that is, the fiducial or timing point. The time series created by the consecutive intervals between the fiducial points is the interbeat interval or HP time series. We recommend using data preprocessing that includes one of the many software solutions that automates the detection of potential heartbeats, followed by interactive visual inspection (e.g., Allen et al., 2007; Bartels et al., 2017; Blechert et al., 2016; de Geus et al., 1995; Kaufmann et al., 2011; Makowski et al., 2021; Martínez et al., 2017; Nabian et al., 2018; Pham et al., 2021; Rodriguez-Linares et al., 2014; Schulz et al., 2009; Vest et al., 2017, 2018; Vollmer, 2019). Artifacts due to technical, movement, and other non-physiological sources or the presence of abnormal beats (e.g., arrhythmias other than veridical beat-to-beat variation) are crucial to identify and address, because artifactual beats negatively impact the quality of the HP time series required to compute averaged HP (or HR) and HRV metrics. Below, we describe the preprocessing steps for an ECG-derived HP times series, but similar principles apply to PPG-derived data.

#### 3.2.1 | Handling technical, movement, and other non-physiological artifact sources

Artifacts may arise from poor electrode contact, faulty conduction by lead wires, extraneous magnetic or power-line noise, excessive movement, myographic signal intrusion, hardware or software errors, and experimenter-(or algorithm-) induced errors during preprocessing. This may lead to spurious labeling of large positive-going deviations in the ECG signal as R waves, or to veridical R waves being missed. Spurious and missed beats can also be introduced by variations in peak amplitude, such as those related to breathing.

In the presence of spurious (extra) R waves, the true HP can be restored precisely by simply summing the two (or more) spuriously short periods that constitute the true HP. When an R wave goes undetected (missed beat), the true location of the missed beat within the spuriously long R–R interval may be unknown making it impossible to precisely determine the location of the undetected beat(s) from the R–R interval series alone. Several approaches to the resolution of missed beats can be used. In order of preference, these are (a) measuring the actual R–R intervals, if visually identifiable, from the ECG record, and manually marking the R wave, (b) using adjacent beats to interpolate the missing R waves; or (c) splitting the spuriously long (misidentified) beat into two (or more) equivalent R–R intervals. None of these approaches will seriously bias estimates of the overall mean of the HP series (for any multi-second or longer epoch), although the latter two approaches may affect the variance, and hence HRV metrics.

Both spurious and missed beats produce large deviations in estimated HPs that usually can be identified visually in graphical displays of the HP series. Consistent assessment and correction of such deviations as described above can be tedious when done entirely interactively, especially with large numbers of participants or long-term recordings. This has led to the development of automated preprocessing strategies that range from simple smoothing or filtering of the digitized data to peak-detection algorithms that detect potentially spurious beats based on the extent of variability in the duration of nearby HPs (Berntson et al., 1990; Lipponen & Tarvainen, 2019) and algorithms that use newer analytic techniques, such as independent components analysis or wavelet-transform-based approaches, to improve the accuracy of detecting R waves (Neha et al., 2021a; Rincon Soler et al., 2018; Sahoo et al., 2020). A follow-up of automated approaches by interactive visual inspection is recommended, because automatically detected outliers are not always due to technical, movement, and other non-physiological artifacts, but can also be caused by abnormal sinus beats. An ectopic beat may reset or otherwise alter the ongoing cardiac rhythm. Thus, it may not be possible for the HP time series to be simply “corrected,” since summing multiple short beats cannot restore a normal RR interval if the short interval contains an ectopic beat. Visual inspection may in some cases justify automated corrections and in other cases point to substantial occurrence of ectopic beats, which then requires user-guided tailoring of automated detection algorithms or even manual peak-picking.

#### 3.2.2 | Handling abnormal sinus beats

**FIGURE 5:**
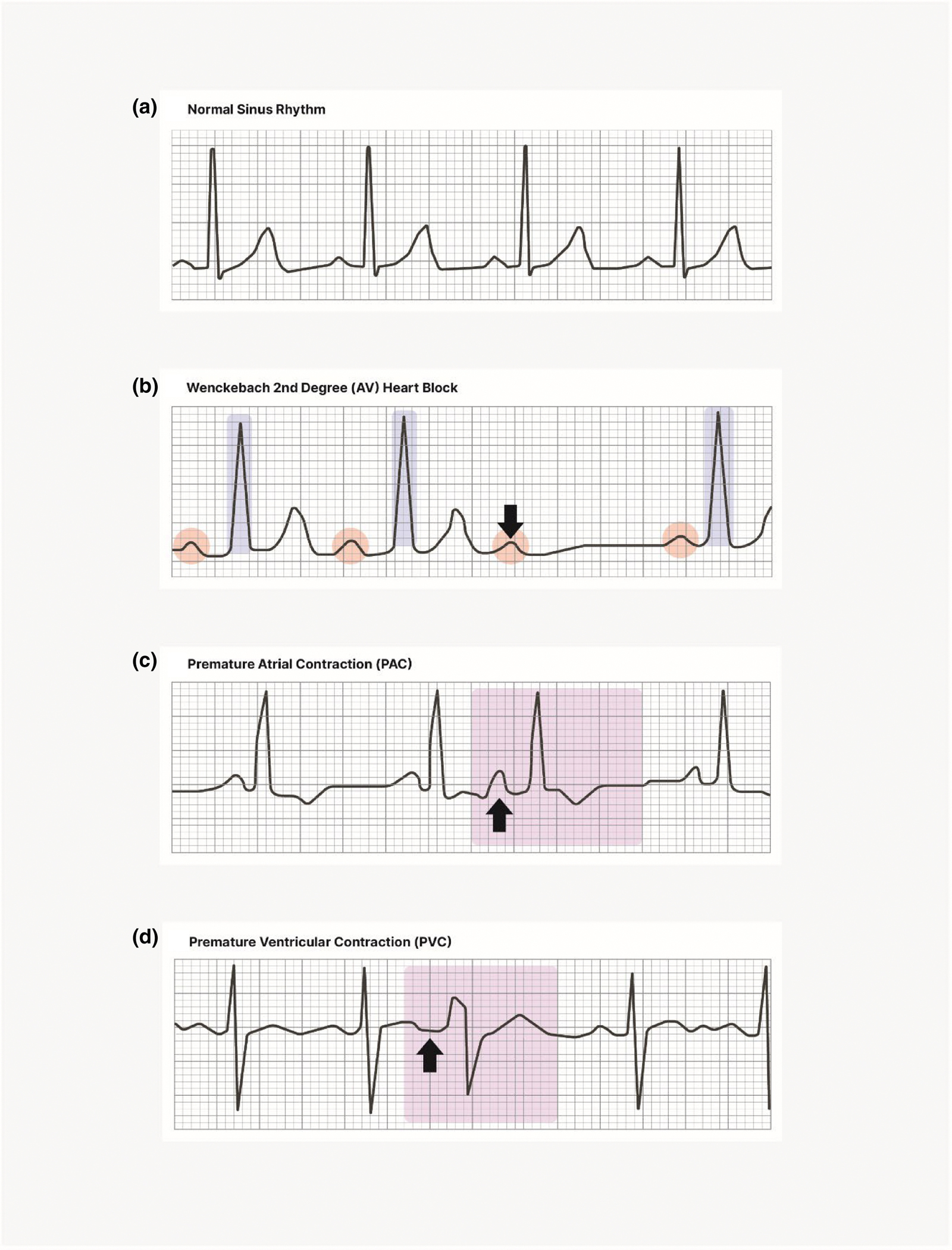
Normal sinus rhythm and sample cardiac arrhythmias. (a) Normal sinus rhythm. Note the small positive-going P wave just before the large QRS complex. (b) Wenckebach second-degree heart block. Note the presence of a P wave (arrow) not followed by a QRS complex (conduction failure). (c) Premature atrial contraction (PAC), with an often atypical (but not always) P wave, followed by a QRS complex. (d) Premature ventricular contraction (PVC). Note the generally widened and abnormally shaped QRS that is not preceded by a P wave (see arrow).

Artifacts of physiological origin can occur when HPs are not generated by the SA pacemaker cells (i.e., not due to normal sinus rhythm). Premature (or ectopic) beats can be produced within either the atria or ventricles, resulting in an atrial premature contraction (APC) or ventricular premature contraction (VPC), respectively. Figure 5 illustrates APCs and VPCs, and for comparison, also shows both normal sinus rhythm and another type of arrhythmia (AV heart block) that can alter beat-to-beat timing. Studies with a moderate or larger sample size and those with older or less healthy samples are especially likely to yield participants having abnormal sinus beats. Many of these are benign, but strings of abnormal beats generated by the ventricle require medical attention. Familiarity with the varieties of harmless and harmful abnormalities is advisable. Use of a standard text such as Goldberger et al. (2018) or Dubin (2000) is recommended to provide guidance for research staff working in psychophysiological laboratories.

If a researcher’s interest is in the normal rhythm of the heart with HP or HR as the metric of interest, analyses can be limited to data segments that are free of abnormal sinus beats. This can lead to selection bias, however, and any bias would be exaggerated with increasing numbers of abnormal beats. This problem should be acknowledged explicitly, and some indication of the extent of data selection should be reported. When the metric of interest is HRV, handling of ectopic beats requires considering whether the cardiac rhythm has been reset by the ectopy (Mateo & Laguna, 2003). When the rhythm is not reset (most commonly when the ectopic focus is ventricular, for example, in the case of a PVC; see Figure 5d), the effect of the ectopic beat on the HRV can be addressed by assigning the ectopic beat to the midpoint between the two beats adjacent to the ectopic beat. Because there is no rhythm reset, this will minimally impact the HRV. In the case of a reset of the cardiac rhythm (as most typically happens when the ectopic focus is supraventricular, for example, as may happen with A/V block or a PAC, see Figure 5b,c), this approach will not work. In this case, part of the affected data should be removed (e.g., removal of part of the time series). In some individuals, the number of abnormal beats can be large. In such cases, reporting the concordance between results from segments of normal R–R intervals and from interpolated data or where data have been removed would raise confidence in the outcomes.

#### 3.2.3 | Choosing an HP measurement epoch duration

The optimal choice of HP epoch duration depends on the psychological phenomenon of interest and the question at hand. A classical approach is to choose an epoch of several minutes (or seconds) of a pre-stimulus period from which one calculates a baseline HP and a comparable period of some minutes (or seconds) of a post-stimulus period to calculate a task HP. Using this approach, questions typically take the form of a reactivity or change score: for example, What is the phasic change in HP over some relevant post-stimulus period compared with the baseline? Change scores may differ across different stimulus conditions, which typically reveals how these conditions differ in their autonomic effects on the heart. However, change scores can partly depend on baseline levels, and these effects can be considerable when there is large between-subjects variation in baseline levels. When comparing stimulus-induced within-subject changes across groups in between-subject designs, the potential physiological dependency of change scores on baseline levels should be assessed and reported. In practice, a covariate approach is frequently used whenever a significant correlation is encountered. However, the phenomena of autocorrelation (i.e., data points closer to each other in time are more correlated) and regression toward the mean requires a different null hypothesis than assuming that a baseline and a change score are uncorrelated (Tu & Gilthorpe, 2007). Various approaches have been suggested to assess the “true” dependency of change scores on baseline when justifying the use of covariance analysis (Geenen & van de Vijver, 1993; Tu & Gilthorpe, 2007).

Other work explicitly considers temporal effects relative to the phase of a single beat, which requires synchronization of clock time and cardiac time at <1-s resolution. For example, in classic studies of cardiac cycle time effects, both reaction times and cardiac timing itself have been shown to be altered by the timing of a stimulus presentation and response initiation relative to the timing of the heartbeat (Grund et al., 2022; Jennings & van der Molen, 2005; Jennings et al., 1991). More recently, there has been considerable interest in how freely generated movements are timed with respect to the heartbeat (Galvez-Pol et al., 2020; Ohl et al., 2016); see also Sherman et al. (2022). In these cases, tight synchronization of the timing of stimuli or responses with respect to cardiac timing is important.

## 4 | HEART RATE VARIABILITY (HRV)

**FIGURE 6:**
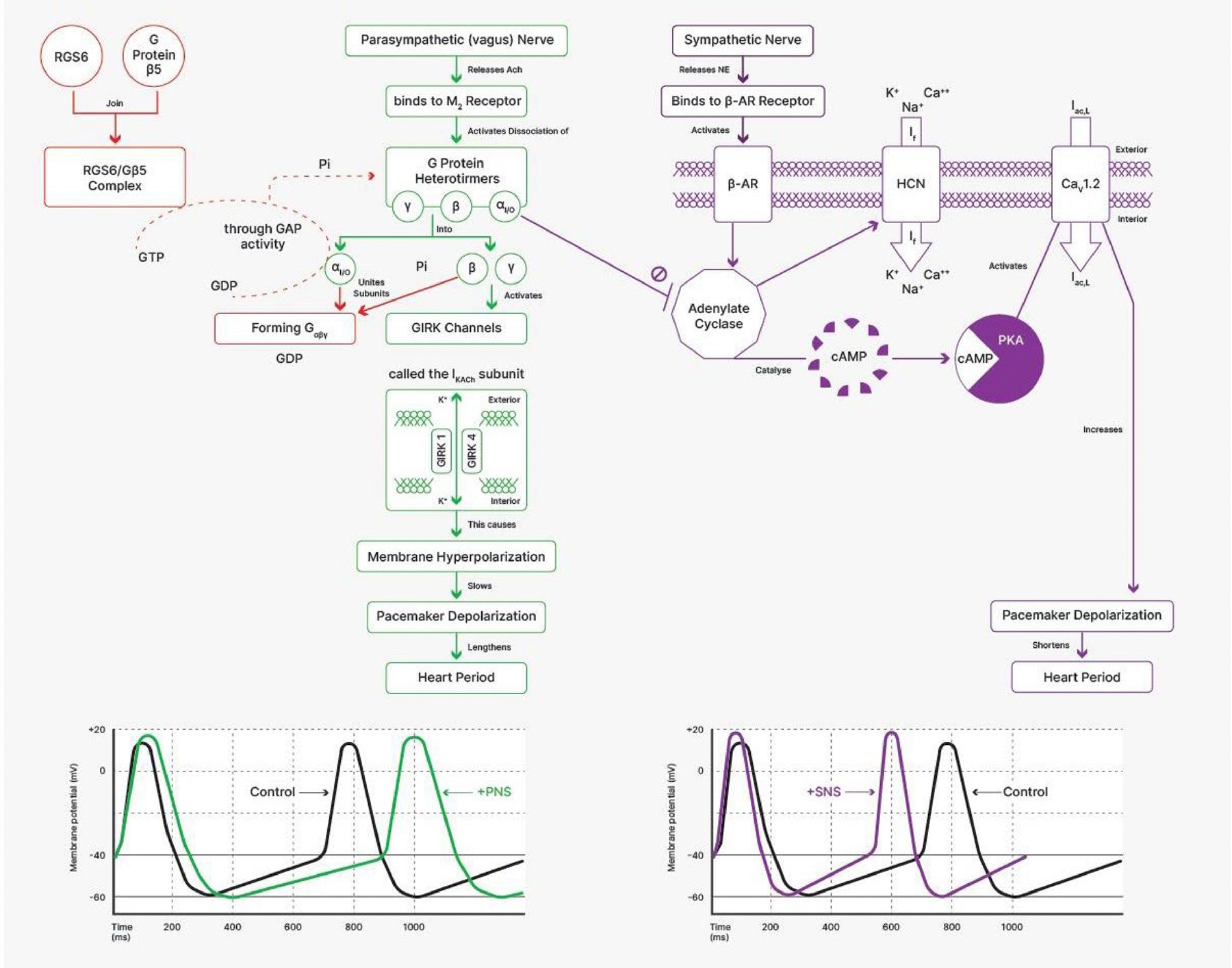
Extrinsic regulation of pacemaker cell activity by parasympathetic nervous system (PNS) and sympathetic nervous system (SNS) activity. Signaling pathways that translate SNS and PNS activity to effects on the depolarization of the sinoatrial pacemaker cells (upper part) translating into lengthening (PNS) and shortening (SNS) of the heart period (lower part). Upper part of the figure redrawn from Supplementary Figure 9 by Nolte et al. (2017) under the Creative Commons Attribution International License.

**FIGURE 7:**
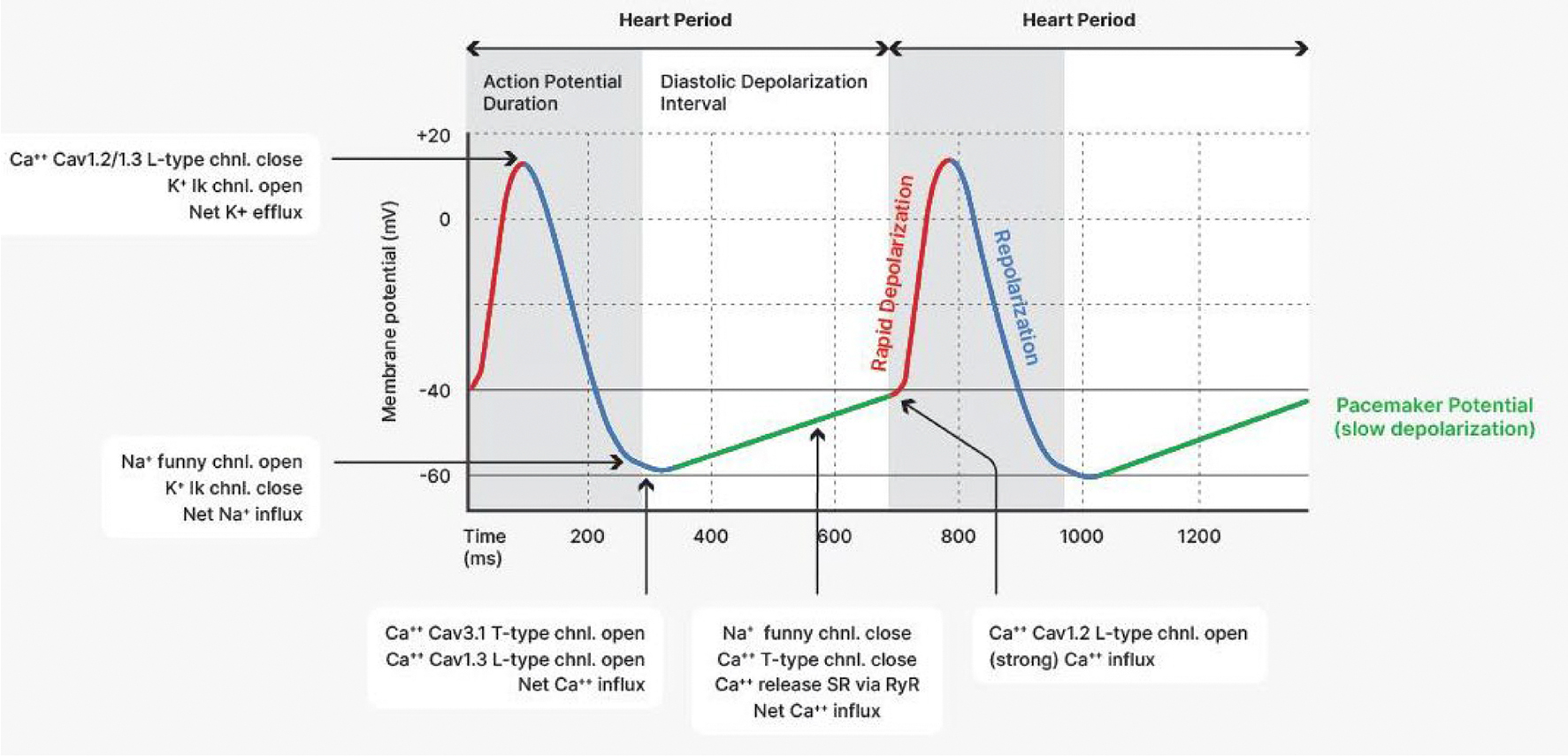
Slow and rapid depolarization, and repolarization phases of the pacemaker potential.

**FIGURE 8:**
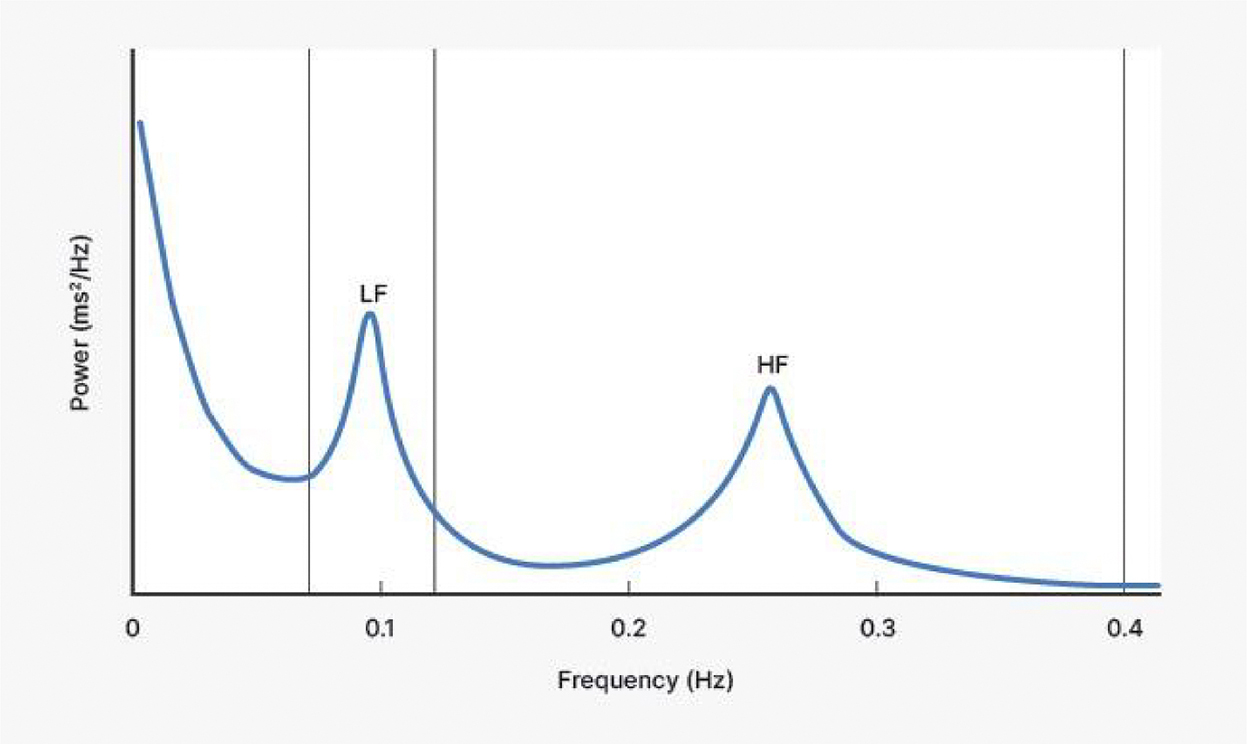
Two prominent frequency peaks in a frequency decomposition of heart rate variability (HRV). Squared variation in ms at a particular frequency (power) is shown for all frequencies of variation in heart period (HP) from 0 to 0.4 Hz. Very slow and ultra-slow frequencies, for example, diurnal variation, are shown on the left (below 0.05 Hz) and are rarely assessed except in chronobiological research (see Section 4.4).

The interval between adjacent R waves in the ECG is not fixed. This interval tends to be variable or irregular over time. This variability in heart period is a result of dynamic interactions between influences on the sinus node of the heart referred to as extrinsic (e.g., autonomic and humoral) and intrinsic (e.g., mechanical stretch and intracardiac cell-to-cell) (for more detail, see Figure 6, Box 2, Figure 7, and Figure 12). These influences induce cycles of speeded or slowed sequences of HPs. The periodic components of this variability in the HP time series are known to cluster around two prominent frequency peaks, which can be exploited to derive noninvasive measures of autonomic cardiac effects. Figure 8 illustrates these two prominent peaks by showing the power (corresponding to the variance across frequencies) in the low-frequency (LF) and high-f requency (HF) bands typically observed at rest.

Although the importance of periodicity in cardiac rhythms has been recognized for centuries, the study of variability in the timing of consecutive beats, HP variability, became central to psychophysiological investigations only with the development of the ECG and subsequent advances in signal processing (see Berntson et al., 1997, pp. 623–625, for an expanded historical overview). Confusingly, history has endowed us with the terminology of heart *rate* variability (HRV) at the expense of the more appropriate heart *period* variability (HPV). Note that HR is not a concept defined at the individual beat level but derives from counting beats per time unit (typically, a minute). Thus, expressing beat-to-beat variability in HR terms does not make sense. Even so, the term HRV is now strongly ingrained in the field and we will therefore adhere to the common practice of labeling the beat-to-beat variability in HP as “HRV.”

For psychophysiologists, there is a natural attraction to use (changes in) HRV as measures of (changes in) autonomic activity related to psychological traits and states and other behavioral phenomena. Accordingly, as the field of psychophysiology developed, utilization of HRV measures expanded concurrent perspectives related to HP, giving rise to three general early theoretical perspectives on their interpretation. First, HRV measures were conceptualized as “trait-like” individual difference variables that reflected behavioral and autonomic phenotypes (e.g., Porges, 1972; Thackray et al., 1974). Another perspective conceptualized HRV as a state-like variable that could be used to index aspects of mental effort and attentional modulation (e.g., Kahneman, 1973; Porges et al., 1973). Finally, a third trend is reflected in interest in the control of HRV by conditioning or biofeedback techniques (e.g., Dworkin, 1993; Lang et al., 1967). Aspects of these three conceptual perspectives developed somewhat independently, but these siloed trends are rapidly changing due to the role that psychophysiology has come to play in contemporary interdisciplinary research in the basic and medical sciences.

Measures of HRV are now central in psychophysiology (Berntson et al., 1997) and several conceptual or theoretical frameworks utilize HRV as a major feature (Grossman, 2023; Grossman & Taylor, 2007; Lehrer & Gevirtz, 2014; Porges, 2011; Thayer & Lane, 2007). Despite over 100 years of investigation, however, the relative contribution of the central and peripheral mechanisms underlying HRV and their functional significance remain the subject of considerable controversy, ambiguity, and active investigation (Ben-Tal et al., 2012; Costa et al., 2017; Eckberg, 2003, 2009; Eckberg & Team, 2017; Goldstein et al., 2011; Karemaker, 2020; Reyes del Paso et al., 2013). This is due to several complications in interpreting HRV as reflecting central nervous system effects on autonomic activity, which are further amplified when researchers attempt to interpret HRV as *exclusively* reflecting the effects of psychological events on either cardiac parasympathetic or sympathetic activity. Before addressing these complications and how to minimize them in research, we will first review what is known about the relationship between HRV in specific frequency bands and cardiac parasympathetic and sympathetic activity. Although our primary focus is on the HF band that dominates the psychophysiological literature, we also discuss in some detail LF rhythms.^2^ For completeness, we also briefly consider HRV in the ultralow- and very low-frequency bands (for a review, see Rana et al., 2020).

### 4.1 | HRV and its relation to cardiac autonomic activity

At the outset it is important to distinguish between the mean (tonic) level of cardiac parasympathetic and sympathetic activity, and the rapid beat-to-beat (phasic) variations superimposed on this mean autonomic activity. It is exclusively the phasic modulation of tonic cardiac parasympathetic and sympathetic nerve activity that generates HRV. The extent to which HRV also reflects tonic levels of parasympathetic and sympathetic cardiac control depends on the extent to which these tonic levels determine the phasic variation in cardiac parasympathetic and sympathetic activity. If the phasic variation in autonomic activity is an exclusive (and ideally linear) function of the tonic level of autonomic activity, then assessment of phasic variation by an appropriate HRV metric can be used to measure tonic levels of autonomic control, as well as changes in this level induced by experimental conditions (e.g., emotional, affective, or cognitive). Factors that influence HRV independent of tonic levels of autonomic cardiac control will necessarily complicate the use of HRV as a measure of (changes in) tonic autonomic activity.

There are two major sources of phasic variation in cardiac parasympathetic and sympathetic nerve activity: the baroreflex and cardiorespiratory gating. Afferent carotid, aortic, and cardiopulmonary baroreceptors are the sensors of the afferent (sensory) limb of the baroreflex, which responds to short-term arterial blood pressure variations. The baroreflex serves to buffer blood pressure fluctuations by (1) correcting for *increases* in blood pressure by increasing cardiac parasympathetic activity, and decreasing cardiac and vascular sympathetic activity, or (2) correcting for *decreases* in blood pressure by decreasing cardiac parasympathetic activity and increasing cardiac and vascular sympathetic activity. In humans, mean arterial blood pressure demonstrates a regular periodic pattern of increases and decreases of a few mmHg with a center frequency of about once per every 10 s, or 0.1 Hz, with variation at this frequency referred to as the Mayer wave (Julien, 2006; see also footnote 2).

**FIGURE 9:**
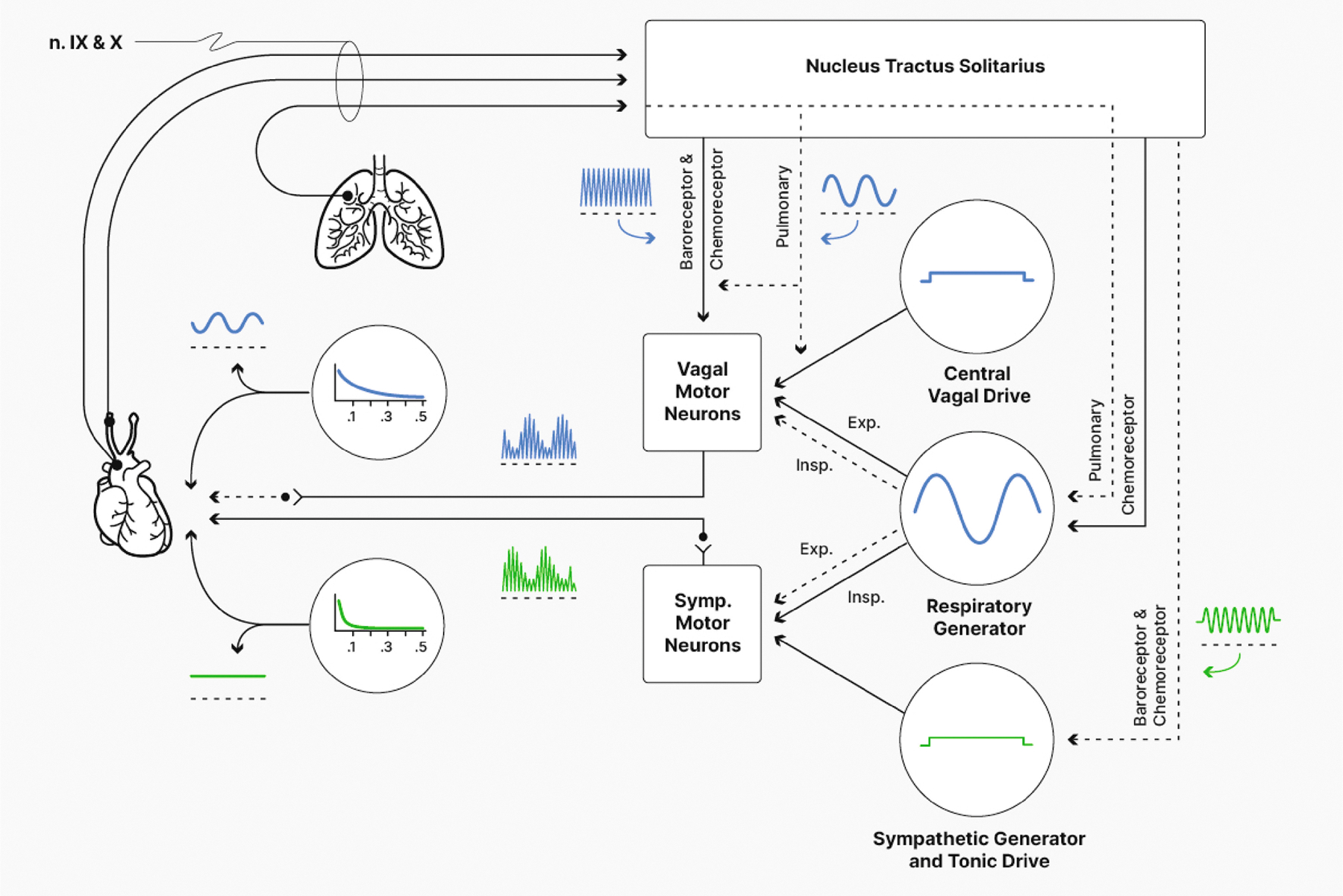
Origins of respiratory sinus arrhythmia (RSA). (Redrawn from Berntson, Cacioppo, & Quigley, 1994). Most cardiorespiratory coupling leading to RSA occurs by inhibiting and enhancing effects of the respiratory generator on the activity of parasympathetic and sympathetic motor neurons. This modulates the tonic input from their generating circuits to yield a respiratory rhythm in the output of these visceromotor neurons to the sinoatrial (SA) node. These effects are mirrored for parasympathetic and sympathetic activity. Parasympathetic activity is enhanced during expiration, whereas sympathetic activity is enhanced during inspiration. Superimposed on the respiratory rhythm are rhythms caused by input from the baroreceptors, chemoreceptors, and pulmonary stretch receptors. Prolonged or exaggerated lung inflation induces the Hering–Breuer reflex through stretch receptors, which terminates inspiration and prevents over-inflation of the lungs. The latter can directly influence brainstem processing to induce respiratory patterns in autonomic activity linked to heart rate variability (HRV). During normal respiration, however, the Hering–Breuer reflex plays little role, and changes in the functioning of these stretch receptors by disease or artificial stimulation also does not substantially impact respiratory-linked HRV (e.g., only around 10 percent of the amplitude of HRV could be attributed to such reflex effects; Koh et al., 1994). The induced rhythms in the sympathetic and parasympathetic nerves to the SA node do not translate to similar periodic patterns in HP due to differential filter characteristics of the SA node for norepinephrine- versus acetylcholine-mediated neurotransmission, indicated in green and blue, respectively. See text for discussion.

*Cardiorespiratory gating* refers to respiratory rhythms found in parasympathetic and sympathetic nerve activity influencing the pacemaker cells of the sinoatrial (SA) node that drive the rhythmic electromechanical activity of the heart (see Figure 9). These respiratory rhythms in autonomic activity at the SA node arise through interactions between cell groups in the brainstem that contribute to the generation of the respiratory rhythm and cell groups that contribute to autonomic cardiovascular control (Dutschmann & Dick, 2012). These interactions lead to a relative inhibition of parasympathetic activity and a reciprocal increase in sympathetic activity during inspiration and, in contrast, a relative inhibition of sympathetic activity and an increase in parasympathetic activity during expiration. These effects of breathing on chronotropic function via predominantly autonomic mechanisms give rise to what is known as respiratory sinus arrhythmia or RSA (Berntson et al., 1993a, 1993b; Eckberg, 2003). “Respiratory” simply denotes the specific origin of the rhythm, “sinus” refers to the sinoatrial node, and “arrhythmia” denotes deviation from a steady or metronomic rhythmicity. RSA is mostly reflected in the HRV around the HF peak, as the band from around 0.12 to 0.4 Hz^3^ generally contains the average respiratory frequency in adult humans.

#### 4.1.1 | Filter characteristics of the SA node for phasic parasympathetic and sympathetic activity

**FIGURE 10:**
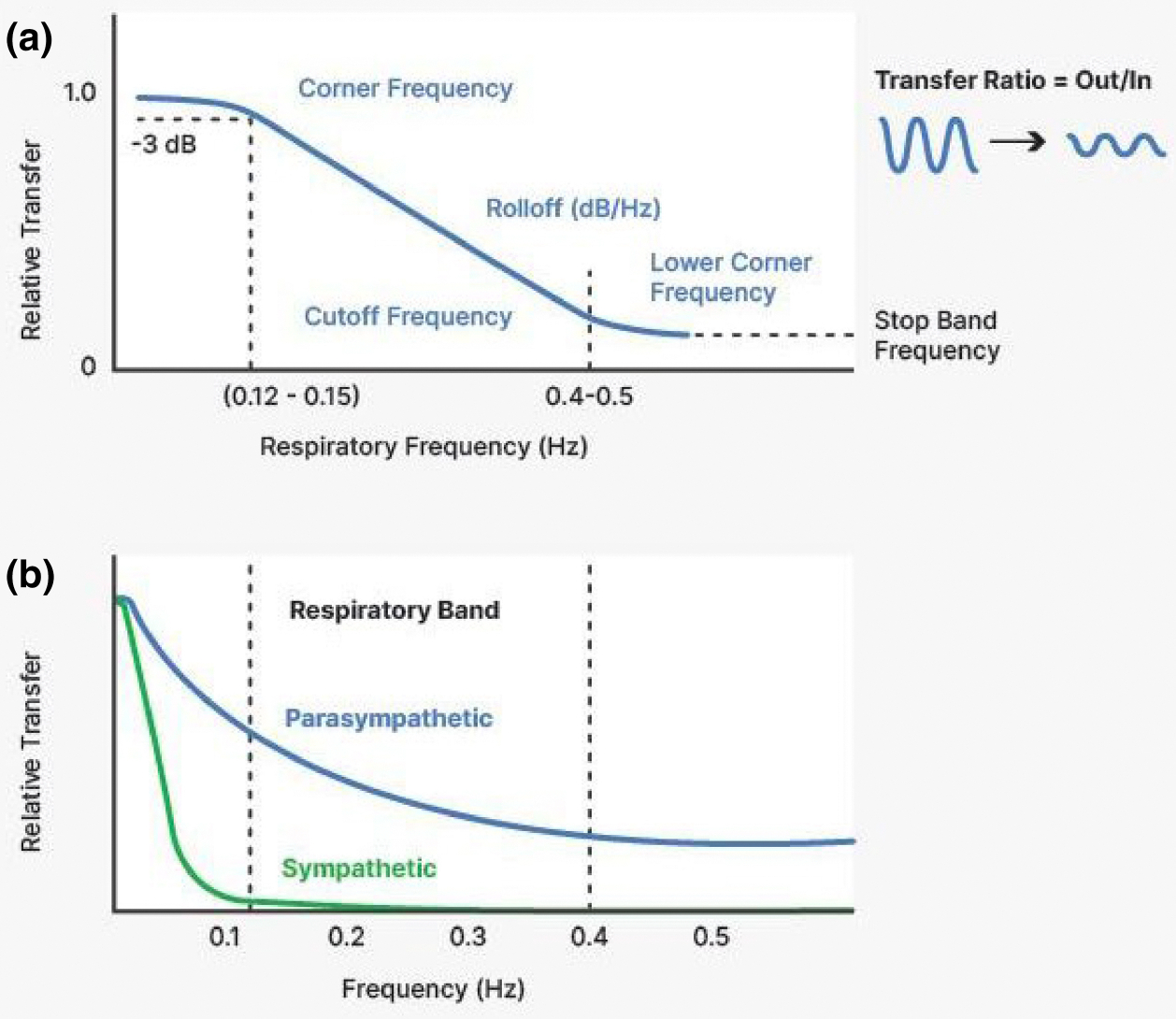
Transfer function (a) and how it differs for parasympathetic (b—blue line) and sympathetic (b—green line) nerve traffic. Panel (a) illustrates relevant transfer function terminology and describes how stimulation frequency is transferred largely undiminished (indexed as 1.0) up to a corner frequency where transfer begins to decrease (Rolloff) and then a point (lower corner frequency) where transfer effectively ceases. In Panel (b), these features can be seen as transfer function graphs for the parasympathetic (blue) and sympathetic (green) inputs to the heart.

Although both cardiac parasympathetic and sympathetic nerve activity show rhythmicity induced by the baroreflex and cardiorespiratory gating, the transfer of this activity into actual effects on HP by the SA node substantially differs for SNS and PNS activity. This is a result of the specific filter characteristics of the SA node. For both parasympathetic and sympathetic responses, the SA node effectively acts as a low-pass filter, with the addition of a delay in the case of the sympathetic response. The vagal filter has a nominal corner frequency of approximately 0.12–0.15 Hz (see Figure 10, also see footnote 3), with the gain falling to around 80% of direct current (DC) by 0.5 Hz. Berger et al. (1986, 1989) found that the response characteristics and particularly the lower corner frequency varied slightly as a function of the mean vagal stimulation frequency. At lower stimulation frequencies, the DC intercept was larger and the corner frequency lower than at higher frequencies. Similar results were found by Mizuno et al. (2010) in rats, suggesting the generality of these findings in mammals. Berger and colleagues also examined the response of the SA node to sympathetic stimulation. Consistent with the more dampened impulse response characteristics observed by Spear et al. (1979), Berger et al. (1989) found that a sympathetic frequency response cutoff between 0.01 and 0.02 Hz was consistent with a low-pass filter.

In Figure 10 Panel A, we illustrate how filtering with specific corner frequencies and roll-off characteristics affects the relative transfer of the amplitude (gain) of a periodic signal in response to manipulating the frequency of the signal. Panel B shows how the SA node affects vagally (blue) and sympathetically (green) induced HRV as a function of the frequency components present in HRV. For both autonomic branches, there is a decreasing transfer of the oscillations in nerve activity into effects on HP at faster breathing rates. However, for vagal nerve traffic some transfer is still present in the typical respiratory frequency band (0.12–0.40 Hz), whereas the effect of sympathetic nerve traffic is apparent primarily in the lower portion of the low-frequency band (0.05–0.12 Hz).

Overall, studies reveal that the cardiac response to parasympathetic activity is rapid, typically within the same or the subsequent heart period interval, whereas that to sympathetic activity is characterized by a time delay (of around 1.7 s) and a transfer of effects only at slower frequencies into an observable end-organ response. The differences in delay of the cardiac response to sympathetic and parasympathetic activation appear to relate largely to SA node receptor processes and postsynaptic responses along with the timing of the stimulus within the cardiac cycle (Somsen et al., 2004). Hill-Smith and Purves (1978) determined that the response characteristics were not due to differences in diffusion within the synapse to the muscarinic or adrenergic receptors, because iontophoretic delivery of either acetylcholine or norepinephrine to within 5 μm of the cell surface still resulted in a delay, suggesting this delay arose from processes subsequent to ligand binding at the postsynaptic receptor. Consistent with this interpretation, Hille (1992) demonstrated that changes in ionic currents by muscarinic receptor activation are mediated by signaling located largely within the cell membrane. In contrast, adrenergic effects are initiated in the membrane, but also require second-messenger activation of a protein kinase in the cytosol, adding delay to the process (Hille, 1992). These features, plus differences in the rate of termination of receptor action (Levy et al., 1993) appear to underlie differences in the time constants of the parasympathetic and sympathetic responses. These findings are consistent with observations in humans that the parasympathetic nervous system can modulate HP effectively at all frequencies between 0 and 0.4 Hz, whereas the sympathetic nervous system modulates HP with significant gain only below around 0.12 Hz.

### 4.2 | RSA and cardiac parasympathetic activity

The above suggests that, at typical breathing frequencies, HF HRV (as a measure of the phenomenon of RSA) has the potential to selectively index phasic changes in parasympathetic activity rather than phasic changes in sympathetic activity. Provided the phasic changes in parasympathetic activity scale with the mean ongoing level of cardiac parasympathetic activity, HF HRV could then be used to measure individual differences in tonic levels of parasympathetic activity, as well as changes in this level induced by experimental manipulations. The dependency of RSA on tonic levels of parasympathetic activity is now generally accepted with support from both direct nerve stimulation/assessment and blockade studies. Within-subject variations in mean vagal nerve outflow have been shown to be related closely to resting HP and to HP fluctuations associated with respiration and baroreceptor activation (Bittiner & Smith, 1986; Katona et al., 1970; Koizumi et al., 1985; Lumbers et al., 1979; Taylor et al., 2001; Yang & Levy, 1984). Consistent with earlier findings in dogs (Akselrod et al., 1981), Pomeranz et al. (1985) reported that cardiac parasympathetic blockade in humans eliminated all HP fluctuations above 0.15 Hz and around 75% of those below 0.15 Hz, whereas sympathetic block-ade did not attenuate and often enhanced fluctuations above 0.15 Hz. These findings have been repeatedly replicated (Berntson, Cacioppo, Binkley, Uchino, et al., 1994; Berntson, Cacioppo, & Quigley, 1994; Cacioppo et al., 1994; Grossman & Kollai, 1993; van Roon et al., 2004).

The increases in RSA with sympathetic blockade may arise from a number of factors, including (a) local sympathetic/parasympathetic interactions (“accentuated antagonism”) at the level of the SA node, and (b) central cardiovagal potentiation or modulation of frequency-dependent oscillations (Bittiner & Smith, 1986; Taylor et al., 2001; Yang & Levy, 1984). These findings confirm the differential frequency response of the sinus node to parasympathetic and sympathetic activity and indicate that high-frequency cardiac rhythms are mediated primarily by vagal innervation of the SA node, although they may be modulated by sympathetic control. This could be a consideration when large changes in sympathetic control are exhibited (e.g., with exercise; Casadei et al., 1996). Finally, the relationship between cardiac parasympathetic activity and HP or RSA can also be dissociated if peripheral transduction of efferent outflow is interrupted by, for example, blockade at the sinus node or conduction disturbances such as those that may occur with drugs or in clinical disorders (Katona et al., 1977).

#### 4.2.1 | HF HRV and RSA

Although HF HRV may be useful as an *index* of RSA, HF HRV and RSA are not identical. RSA is a respiratory-related cardiac chronotropic biological rhythm, whereas HF HRV is the variation of the heart in a pre-determined “respiratory” frequency band (typically 0.12–0.40 Hz) corresponding to respiration rates between 7.2 and 24 breaths per minute. This frequency band will capture mean respiratory behavior of many human adults in most conditions, but not necessarily of *all* individuals in *all* conditions. Also, even if the peak respiratory frequency for an individual is 12 breaths per minute, that individual may take a substantial number of longer or shorter breaths (see Grossman, 1992, Figure 8). Therefore, a part of the respiratory-related and vagally mediated influence can spill over into a considerable portion of the LF band, and if so, would not be visible in the HF HRV measure that is bounded by a frequency cutoff (e.g., breathing slower than 0.12 Hz). In such cases, using the label “RSA” for HF-HRV power would be imprecise. If one wishes to specifically infer that the HF HRV metric reflects RSA, it is always necessary to state that the HF HRV may be compromised by respiratory fluctuations outside the selected HF HRV band, or by rhythmically occurring external events that affect HP that occur at a frequency within this respiratory band (e.g., such as stimulus presentations presented at frequencies within the selected HF HRV band).

#### 4.2.2 | RSA and the cardiac vagal baroreflex

The baroreceptor cardiac reflex is measured as the HP response (in ms) per mmHg change in blood pressure. Between-subjects correlations between RSA and different measures of baroreflex control are on the order of 0.6 (Bigger & Schwartz, 1994; Grossman et al., 1996). In addition, a linear within-subjects relationship has been reported between RSA magnitude and vagal baroreflex response during pharmacological manipulation (Bloomfield et al., 1998). A respiratory-associated rhythm in blood pressure (Traube–Hering wave; Barnett et al., 2020) could, via the baroreflex arc, contribute to RSA (Karemaker, 2009). Indeed, there is a clear decrease in RSA with baroreceptor denervation (Rimoldi et al., 1990). Notably, with brief arterial baroreceptor stimulation in humans, the minimum reflex latency is around 0.25 s, the time-to-peak effect is around 2.5 s, and the decay of the response is around 2.0 s (Eckberg, 1976, 1980; Eckberg & Eckberg, 1982); see also Borst and Karemaker (1983). These delays appear incompatible with a substantive contribution of baroreceptor reflexes to RSA (for review of RSA and relationships to baroreceptors and sympathetic activation beyond immediate reflex control, see Eckberg, 2003). Rather, the predominant determinants of RSA appear to be central respiratory rhythm generators, pulmonary stretch receptors, and other reflexes (for reviews, see Berntson et al., 1995; Dutschmann & Dick, 2012; Farmer et al., 2016).

#### 4.2.3 | Is there a functional or physiological role for RSA?

The prevalence of RSA and its widespread appearance across species suggest that it may have a physiological function. This, however, has defied clear description. Three suggestions have been made with equivocal support for each, namely RSA has been postulated to: (a) enhance pulmonary gas exchange by clustering more heartbeats during inspiration, which could enhance lung perfusion and oxygen extraction (Giardino et al., 2003; Hayano & Yasuma, 2003), (b) improve cardiac efficiency while maintaining physiological levels of arterial carbon dioxide (Ben-Tal et al., 2012), and/or (c) stabilize systemic blood pressure and blood flow (see review by Elstad et al., 2018). Notwithstanding these postulates, a complete, precise, and fully supported understanding of the possible functional role of RSA has not yet been achieved.

### 4.3 | LF HRV and its relation to cardiac sympathetic activity

In accord with the temporal dynamics of the parasympathetic and sympathetic cardiac innervations outlined earlier, both autonomic branches can influence lower frequency cardiac rhythms, typically defined as HRV in the 0.05–0.15 Hz frequency band (although a lower limit of 0.04 has also been used).

Published literature on autonomic mediation of LF HRV is controversial. A strong claim is that LF HRV indexes sympathetic nervous system activity. Some researchers, notably Malliani and associates (Malliani et al., 1991; Pagani et al., 1986), argued initially that LF HRV reflects mainly fluctuations of sympathetic traffic to the SA node. Because sympathetic nerve traffic to the human heart has not been measured specifically or directly, conclusions regarding the mediation of LF HRV are based on indirect evidence. Sympathetic traffic to skeletal muscle (Saul et al., 1990) and the vasculature (Guzzetti et al., 1994; Koh et al., 1994) both fluctuate at low frequencies. In accord with the low-pass characteristics of sympathetic cardiac synapses, LF HRV has been reported to be reduced by pharmacological blockade of cardiac sympathetic receptors (Akselrod et al., 1981; Murphy et al., 1991; Pomeranz et al., 1985) or stellectomy (Pagani et al., 1986). Consistent with these observations, numerous investigators have found that sympathetic activation and parasympathetic withdrawal, known to occur with maneuvers such as tilt, are reflected in a relative shift from higher to lower frequency HRV. Also consistent are reports that sympathetic activation triggered by vasodilators can lead to enhanced LF HRV (Pagani et al., 1986).

However, other evidence refutes LF HRV as an index of sympathetic nervous system activity. β-adrenergic blockade does not always appreciably reduce LF cardiac rhythms and can even enhance them slightly (Cacioppo et al., 1994; Taylor et al., 1998). The LF HRV, whether expressed in absolute or normalized units, does not change consistently with other pharmacological manipulations that enhance or reduce sympathetic adrenergic influences on the heart (Ahmed et al., 1994; Jokkel et al., 1995; Kingwell et al., 1994; Saul et al., 1990). In addition, changes in LF HRV do not correspond to variations in direct measurements of norepinephrine spillover in the heart or other tissues (Kingwell et al., 1994; Saul et al., 1990), nor do they significantly correlate with circulating catecholamines (Sloan et al., 1996). Additional studies have clearly indicated that LF rhythms are influenced by both sympathetic and parasympathetic mechanisms (Kromenacker et al., 2018; Pomeranz et al., 1985; Saul et al., 1991). Atropine, a selective parasympathetic antagonist, produces a dose-related reduction in LF HRV, with an eventual elimination of this cardiac rhythm at doses corresponding to complete parasympathetic blockade (Akselrod et al., 1981; Cacioppo et al., 1994; Koh et al., 1994; Murphy et al., 1991; van Roon et al., 2004). These findings suggest that sympathetic activity may not be the sole, nor even predominant, determinant of LF HRV.

A second perspective is that LF HRV is an index of central baroreflex control. The LF rhythms in HP and blood pressure, with a center frequency of around 0.1 Hz, appear to reflect a baroreflex resonance frequency (de Boer et al., 1987; Sleight et al., 1995; van Roon et al., 2004). The conventional LF bandwidth, however, is considerably broader (0.05–0.12 Hz). This broader bandwidth can complicate interpretation as it allows RSA to contribute to the 0.1-H z rhythm, when respiration rate is lower than around 10 breaths/min, and by partial overlap from adjacent slower rhythms that sometimes are observable at a (center) frequency of around 0.03 Hz. Other observations support a relationship to baroreceptor function while implicating parasympathetic rather than sympathetic effects. Tetraplegic patients, with intact central vagal outflow to the heart but severed brainstem-spinal sympathetic pathways, still manifest LF rhythms (Koh et al., 1994). Because descending baroreflex control of sympathetic outflow is abolished in tetraplegics, baroreflex-induced changes in vagal outflow must be responsible for LF HRV in such patients. Similarly, studies of high spinally transected animals show slow arterial pressure rhythms mediated by spinal sympathetic reflexes, and these pressure oscillations in turn evoke baroreflex-mediated rhythms of cardiac parasympathetic response (Polosa, 1984). This result is in accord with several validated models of human baroreflex mechanisms that account for LF HRV in terms of simple cardiac parasympathetic responses to periodic baroreceptor stimulation (de Boer et al., 1987; Madwed et al., 1989). These models are based on known characteristics of each of the components of the baroreflex (e.g., delays and time constants of parasympathetic and sympathetic responses) and correspond closely to observed behavior of the cardiovascular system.

There is considerable evidence for a causal relationship between LF rhythms and baroreflex-mediated cardiac parasympathetic responses to arterial blood pressure fluctuations of sympathetic vasomotor origin. Arterial pressure and HP oscillations of around 0.10 Hz (10-s rhythm) are very tightly linked (Cevese et al., 1995). Selective pharmacological blockade of sympathetic efferent traffic to the vasculature reduces the magnitude of 0.10 Hz blood pressure and HP oscillations (Scheffer et al., 1994), indicating that HP rhythms at this frequency are mediated by vagal baroreflex responses to 0.10 Hz sympathetic vasomotor fluctuations. Similarly, experimentally induced changes in arterial baroreceptor stimulation produce corresponding alterations in LF HRV (Bernardi et al., 1994; Koh et al., 1994; Sleight et al., 1995), and exercise-induced changes in LF HRV appear to be mediated by vagal baroreflex responses to blood pressure rhythms (Mukai & Hayano, 1995). One view is that LF HRV thus reflects cardiac baroreceptor control (Goldstein et al., 2011), which is compatible with other perspectives suggesting that LF HRV does not index sympathetic activity, but rather reflects baroreceptor control, among other factors (Malpas, 2002).

A third perspective is that LF HRV is a complex index of the resonance of the sympathetic nervous system that reflects phasic engagement and disengagement of sympathetic activation (Eckberg, 2000). Sympathetic and parasympathetic systems may interact in complex ways in the generation of LF HRV. Because healthy humans operate along the relatively linear portion of the arterial pressure-vagal baroreflex function (Rea & Eckberg, 1987), changes in blood pressure trigger corresponding changes in vagal-cardiac nerve traffic. Thus, sympathetically mediated arterial pressure changes may translate into vagal-cardiac nerve responses so that the latter may bear some quantitative relation to sympathetic traffic. In this regard, Pomeranz et al. (1985) reported that either cardiac parasympathetic or sympathetic blockade attenuated LF HRV by around 75%. This result suggests a degree of interaction or nonlinear resonance at lower frequencies due to a combination of parasympathetic and sympathetic effects.

#### 4.3.1 | Sympathovagal balance

The Malliani group has further proposed that power in the LF and HF bands, especially when expressed in normalized units reflects the relative balance between sympathetic and vagal effects and that this sympathovagal balance can be indexed by the LF/HF ratio (e.g., see Malliani et al., 1994; Montano et al., 1994). Studies of graded orthostatic challenge, known to increase sympathetic efferent traffic (Burke et al., 1977; Iwase et al., 1987) and decrease vagal outflow, have provided the most consistent evidence of an association between normalized LF HRV and grade of head-up tilt, although the correlation coefficients are rarely above 0.7 (Bootsma et al., 1994; Montano et al., 1994). Cardiac sympathetic activation induced by exercise, however, evoked a decrease rather than the expected increase in LF HRV, whether calculated in absolute or normalized units (Ahmed et al., 1994; Arai et al., 1989; Perini et al., 1990).

Others have argued that the LF/HF ratio cannot be considered as an index of sympathovagal balance and argued that separate reporting of LF and HF is more meaningful than using a ratio (e.g., see Billman, 2013; Eckberg, 2000). This relates to the observation that the autonomic branches are not always reciprocally controlled, and therefore can vary independently, or demonstrate coactivation or coinhibition (Berntson et al., 1993a; Berntson, Cacioppo, Binkley, Uchino, et al., 1994; Koizumi & Kollai, 1992). For these reasons, the formal status of the construct of a bipolar sympathovagal balance is no longer tenable, nor is the use of the LF/HF ratio to index it.

#### 4.3.2 | Summary of LF HRV and HF HRV

Although both sympathetic and parasympathetic systems are sensitive to respiratory rhythms, the impact of these rhythms on sympathetic cardiac effects are functionally eliminated because of the long latency of effector action after sympathetic stimulation, that is, by the low-pass filter properties of the sympathetic sinoatrial node synapses. Therefore, the evaluation of HRV within the HF band is generally considered to reflect cardiac parasympathetic effects (Berntson et al., 1993b). Lower frequency bands of HRV have also been defined in the literature. Unlike variability in the HF band, however, these lower frequency bands generally reflect a dynamic mixture of sympathetic and parasympathetic rhythms, which renders them less informative as measures of autonomic cardiac activity. Reviews focusing on the human literature conclude that, at least at rest, LF HRV is not an appropriate index of sympathetic activity (Goldstein et al., 2011; Reyes del Paso et al., 2013), but reflects an unknown mixture of sympathetic and parasympathetic effects (Cohen & Taylor, 2002). Without refuting their potential clinical utility, we recommend not using either LF/HF ratio or the LF HRV as selective measures of sympathetic activity in psychophysiological research.

### 4.4 | Brief overview of ultralow (ULF) and very low (VLF)-frequency HRV

Ultralow frequencies are those lower than 0.0033 Hz and include HR rhythms in the circadian range (approx. 24-h cycle; e.g., sleep–wake cycle), and ultradian range (<24-h cycle; e.g., sleep stage cycling across the night). Although these longer ULF rhythms may be of interest, they have been largely out of scope for psychophysiology.

The VLF band for HRV has different definitions across the literature (see Berntson et al., 1997 for additional detail), but it is probably best characterized as the frequencies just below the 0.1 Hz HP and blood pressure rhythms. For the purposes of this *Committee Report*, we consider VLF variability to include frequencies between the 0.0033-Hz lower frequency limit as defined by Bigger et al. (1992) up to frequencies of 0.05 Hz, a commonly used value for VLF oscillations (e.g., Madwed & Cohen, 1991). HRV in the VLF range often dominates recordings of HRV under spontaneous ambulatory conditions (Bigger Jr. et al., 1995). Proposed explanations for VLF rhythms are diverse and numerous, both in the human literature (Cherniack & Longobardo, 1973; Goldberger et al., 1984; Novak et al., 1995; Pomeranz et al., 1985; Saul, Arai, et al., 1988) and non-human animal literature (Madwed & Cohen, 1991; Salomao et al., 2015). McCraty and Shaffer (2015) noted that work in autotransplanted canine hearts (Murphy et al., 2000) could imply a possible intracardiac source of VLF variability, since Murphy et al. (2000) saw cardiac reinnervation by sympathetic and parasympathetic extrinsic fibers, and yet the extent of reinnervation by these extrinsic neurons did *not* co-vary with VLF HRV at 1-year post-transplant. Furthermore, VLF in transplanted dogs was similar to that in control (non-transplanted) dogs, again suggesting the possibility that VLF may arise from intracardiac sources. Other researchers have suggested the possibility of hormonal influences on VLF, since transplant patients can modulate their HRs at very low frequencies with exercise and during 24-h ambulatory activity (Arai et al., 1989; Bernardi et al., 1996).

The etiology of ULF and VLF rhythms and their relevance in clinical conditions remain important areas of research. Regardless of the physiological mechanisms, there remains interest in these slow rhythms because they are strong, independent predictors of cardiac death after myocardial infarction (Bigger et al., 1992, 1996). congestive heart failure (Hadase et al., 2004), and dysregulated fasting glucose levels (Stein et al., 2005).

### 4.5 | RSA as an index of individual differences in cardiac parasympathetic control

As outlined above, activity of the parasympathetic autonomic branch is the major determinant of RSA. HRV measures reflecting RSA are therefore often interpreted as indices of cardiac parasympathetic activity. However, an important distinction should be made between the interpretation of differences in absolute RSA between individuals and changes in RSA across different contexts within a single individual. Somewhat different caveats apply for interpreting RSA in within-subject and between-subject designs.

Evidence for RSA as an index of individual differences in tonic cardiac parasympathetic activity comes from a few reports that described very close associations between RSA and atropine-derived measures of the chronotropic vagal effect at rest (Fouad et al., 1984; Hayano et al., 1991). Other investigations, however, have reported lower between-subjects correlations among the chronotropic effects of parasympathetic blockade and RSA (Grossman & Kollai, 1993; Kollai & Mizsei, 1990; Maciel et al., 1985). In part, this may be due to individual differences in respiratory parameters and in the strength of respiratory-vagal coupling that could influence the RSA level independent of the individual’s cardiac parasympathetic activity. A larger concern is that RSA reflects the *effects* of cardiac parasympathetic activity on the SA node and not cardiac parasympathetic *activity* itself. In a between-subject comparison, all factors that can influence the relationship between vagal activity at the SA node and the effect of that activity on the pacemaker cells become potential confounders of the relationship between vagal activity and RSA. These factors include the sensitivity of the muscarinic receptor and the efficiency of its signaling pathways, which can be influenced by a range of factors including age, sex, genetics, and medication use (see Section 6). In view of these caveats, researchers should exercise caution when inferring individual differences in cardiac parasympathetic activity from individual differences in RSA. We further recommend use of the term cardiac parasympathetic control rather than cardiac parasympathetic activity (or drive or tone) when the focus is on *individual differences* in RSA.

### 4.6 | RSA reactivity as a measure of within-subject changes in cardiac parasympathetic activity

In short-term within-subject designs, the impact of between-subject factors will be greatly attenuated, allowing the changes in RSA across experimental conditions or treatments to be interpreted as changes in cardiac parasympathetic activity with more confidence. However, here too, a number of factors may distort the within-subject relationship between changes in RSA and parasympathetic activity, of which respiratory behavior has emerged as the most prominent factor to be taken into account. Parenthetically, variables that modify RSA may also be for some studies/investigators legitimate dependent measures. That is, modulatory variables, for example, respiratory parameters, muscle activity, and speech, may change directly as a function of experimental manipulations. Such changes are open to interpretation themselves but must be considered modifiers or even confounders of concurrent estimates of RSA.

#### 4.6.1 | Effects of respiration

Changes in respiratory behavior (rate and depth) have a strong impact on HRV measures, and on the phenomenon of RSA (Eckberg, 2003; Grossman et al., 1991; Grossman & Kollai, 1993; Grossman & Taylor, 2007; Kollai & Mizsei, 1990; Saul et al., 1989; Taylor et al., 2001). Within individuals and within the typical range of resting breathing frequencies (e.g., approx. 7–24 breaths/min in adults), RSA is inversely related to respiratory rate and directly related to tidal volume, that is, slowed respiratory rates or deeper inspiratory volumes lead to an increase in RSA (Hirsch & Bishop, 1981). This relationship is the same for variation during spontaneous breathing and due to voluntary (or paced) changes in ventilation (Hayano et al., 1994; Hirsch & Bishop, 1981). Whereas rate is the more potent determinant of RSA amplitude than depth within typical breathing ranges, depth should ideally also be considered (see Egizio et al., 2011; Ernst et al., 1999). Breathing that occurs outside the HF HRV band typically results in lower HF HRV. This is because the respiratory frequency is either below the lower end of the HF HRV band (e.g., respiratory frequencies of 0.03, 0.08, and 0.1 Hz as shown in Schipke et al., 1999) or above the upper end of the HF HRV band (e.g., respiratory frequency >0.5 Hz), where respiratory oscillations can no longer impact the HP (e.g., Schipke et al., 1999; also see Hirsch & Bishop, 1981). Similar effects were not observed when using time-domain HRV measures (see Section 5 below), likely because these reflect the full range of variation in HP rather than just the HF HRV frequencies associated with respiration (Grossman et al., 1991; Hayano et al., 1994; Schipke et al., 1999). Moreover, the relationship between RSA and respiratory parameters may be modulated by stressors or other affectively evocative situations (e.g., Houtveen et al., 2002; Ritz et al., 2020). Attributing within-subject changes in RSA across experimental conditions to changes in cardiac parasympathetic activity would require an adjustment that takes the condition-induced changes in respiratory behavior into account (Grossman et al., 1991). Given the importance of respiratory rate and tidal volume as critical determinants of RSA values independent of efferent cardiac vagal activity, respiratory behavior should be measured (or at minimum estimated, e.g., from the HRV spectrum). Thus, an essential requirement for appropriate interpretation of RSA is a reliable assessment of respiratory behavior, ideally through a concurrently measured respiratory signal that reflects both frequency (respiratory rate) and depth (Houtveen et al., 2006; Ritz et al., 2002; Wientjes, 1992). Recording of the respiratory signal is outside the scope of this article, but excellent sources are available (Lorig, 2017; Ritz et al., 2002).

#### 4.6.2 | Effects of speaking

Respiratory effects of speaking present a special challenge to the interpretation of changes in RSA as reflecting changes in parasympathetic activity. Recording contexts where people are regularly speaking will strongly impact measures of HRV, including RSA (e.g., Beda et al., 2007; Reilly & Moore, 2003; Tininenko et al., 2012). Speech, even in the form of subvocalizations during difficult cognitive tasks, requires complex respiratory maneuvers that naturally disrupt the inspiratory/expiratory rhythm. Indeed, some research suggests that changes in the inspiratory/expiratory ratio, as can happen during speech, impacts RSA, even when mean respiratory rate and depth cannot account for these changes in RSA (Strauss-Blasche et al., 2000). It is often unclear whether a change in RSA reflects parasympathetic effects of the act of speaking itself or the parallel respiratory changes induced by speaking. Given this, changes in parasympathetic activity cannot easily be inferred from RSA during manipulations involving speech. For these reasons, it is problematic to compare recording contexts where people are speaking with those where they are not, that is, when comparing the baseline of the Trier Social Stress Test (TSST) to the part of the TSST when a participant is giving a speech. We recommend deriving RSA estimates for such paradigms from appropriate non-speaking periods (such as preparing to speak and baseline epochs) or using as a comparison a non-stressful speaking baseline.

#### 4.6.3 | Effects of posture

Postural changes have a profound impact on cardiac autonomic activity with a lower mean vagal firing rate and a higher mean sympathetic firing rate during standing (or other upright positions such as head-up tilt) compared with sitting, with even larger differences in a supine position. For this reason, psychophysiologists should avoid confounding psychological manipulations with postural manipulations (as occurred in earlier versions of the TSST where HRV was measured during public speaking in a standing participant compared with a seated resting baseline). An additional complication was already demonstrated in the earlier work of Saul et al. (1989) who showed that the transfer function between respiration and HP is itself affected by posture, such that the corner frequency of the transfer function shifts to lower frequencies when going from supine to an upright position (see also Mukai & Hayano, 1995). Attributing within-subject changes in RSA across experimental conditions or treatments to changes in cardiac parasympathetic activity would require posture to be kept constant, or to adjust RSA statistically in a way that takes posture-induced changes in RSA into account.

### 4.7 | Other caveats in the interpretation of HRV

Apart from the above cautions on respiratory and postural effects, two other concerns have been voiced that should be addressed when interpreting HRV measures to reflect cardiac parasympathetic activity both in between-subject and within-subject designs; dependency of HRV on HP and ceiling effects.

#### 4.7.1 | Dependency of HRV on HP

Claims have been made that HRV is determined, at least in part, by HP and that (changes in) HRV should be “corrected” for (changes in) HP to get a better index of (changes in) cardiac parasympathetic activity. The biological, quantitative, and interpretive issues engendered by this claim have been reviewed in detail elsewhere (de Geus et al., 2019). Both chronotropic measures, HP and HRV, indeed correlate highly, but there are no easy answers to the question of whether and how HRV should be adjusted for HP and knowledge gaps remain with respect to assumptions underlying existing HRV adjustment approaches. Given this, joint examination of both measures is advised because this can guide interpretation. If the goal is to predict cardiac parasympathetic activity, the best approach is to include both RSA and mean HP in the prediction model (Grossman & Kollai, 1993), which can be done by latent variable (e.g., structural equation) modeling or a comparable approach that simultaneously treats HP and RSA as facets of a common construct (de Geus et al., 2019). We do not recommend using a residualized approach to remove the variance in RSA attributable to variance in HP because it will also remove the autonomic effects of potential interest. If an adjustment of HF HRV (or another HRV metric) for HP is nonetheless applied, then the parsimonious coefficient of variation is recommended. When an adjustment is applied, the recommendation is to report both the adjusted and unadjusted results. Associated caveats relate to the fact that RSA may be reduced at very high levels of vagal control (which could be associated with low HR) or high levels of sympathetic control (which could be associated with high HR). These caveats should be considered in interpreting HF HRV.

#### 4.7.2 | Ceiling effects on HRV

At very high levels of sinoatrial receptor occupancy by muscarinic M2- or adrenergic β-receptors, saturation effects may strongly reduce the effects of cardiac parasympathetic or sympathetic activity, causing floor (PNS) or ceiling (SNS) effects on the heart period that will also impact on HRV. For example, RSA is nearly abolished at very high levels of cardiac parasympathetic activity (Anrep et al., 1936a; Eckberg & Orshan, 1977; Goldberger et al., 1994), which can be induced by vasoconstrictive agents (Goldberger et al., 1994, 2001). This finding suggests a ceiling effect on respiratory modulation of parasympathetic effects on the heart and indicates that RSA and cardiac parasympathetic activity can be dissociated. This ceiling effect is expected to cause a quadratic relationship between HP and RSA, with paradoxically low levels of RSA at high levels of parasympathetic activity, which has indeed been found for vigorous regular exercisers during sleep (van Lien et al., 2011). This reinforces the idea that caution should be used in interpreting RSA when high levels of vagal activation can be expected (Malik & Camm, 1993). Such high cardiac parasympathetic activity can be encountered in 24-h recordings during deep sleep (Neijts et al., 2014). We recommend inspection of the relationship between HP and RSA to detect this potential ceiling effect in between-subject studies and in multi-day within-subject studies.

## 5 | QUANTIFICATION OF HRV

### 5.1 | Signal acquisition and preprocessing of the HP time series

The quality of any HRV metric will depend on the quality of the HP time series, and therefore all steps described under Section 3 apply. However, compared with HP and HR, derivation of HRV metrics is even more sensitive to errors in fiducial-point detection accuracy, and errors in turn more strongly impact the ability to reliably detect any experimental or contextual effects, individual differences of interest, and other related phenomena. When a researcher’s interest is in HRV metrics, artifact-laden epochs within a HP series cannot simply be deleted because this would disturb the continuity of the time series, which must be maintained. Very serious biases are known to occur when artifacts go undetected and unresolved, with even a single missed R wave within a 2-min recording epoch increasing estimates of HRV several fold (Berntson & Stowell, 1998). Uncorrected artifacts likewise impact many nonlinear dynamical measures (Voss et al., 2009). This has led to a large number of approaches that try to correct for artifacts in the HP time series preserving its continuity in time (Peltola, 2012). From the many classes of artifact editing, most publications in psychophysiology have favored interpolation methods that replace the deviant HPs with new interpolated HPs.

One popular interpolation method is cubic spline interpolation, where smooth curves are estimated by fitting a third-degree polynomial through multiple adjacent HPs (Kaufmann et al., 2011; Tarvainen et al., 2014). Other approaches may further reduce the impact of missed R peaks on HRV metrics (Citi et al., 2012; Mateo & Laguna, 2003), but additional studies are needed to formulate specific guidance on the extent of “acceptable” bias in HRV metrics when a larger number of beats are corrected. The interpolation approach would be expected to produce minimal distortion in measures from healthy individuals who have relatively few abnormal beats. For recordings with a high rate of abnormal beats across the entire length of the recording (e.g., >1%), HRV analyses could be limited to the global descriptive measures described below. The alternative is to quantify the amount of correction that is required in each condition and ensure that the within- and/or between-subject comparison of HRV metrics are not biased due to the correction steps.

A question that has attracted much attention is the minimal sampling frequency needed to obtain reliable HRV estimates. Although a sampling frequency of 1000 Hz should also be considered the default for HRV, the large variance seen in many HRV measures, even after rescaling by log transforms, will drown out the extra measurement error induced by lower sampling frequencies in between-subject (Ellis et al., 2015) and even within-subject comparisons (Burma et al., 2021). Provided there is interpolation of the HP time series prior to HRV calculation, sampling frequencies can go well below 100 Hz without affecting group comparisons (Ellis et al., 2015) or the intraclass correlation of original and down-sampled ECG signals (Burma et al., 2021).

When using the PPG instead of ECG as the source of HP time intervals, the expectation would be that HRV from PPG is of lower quality, due to the resolution of R-wave peak picking and vascular issues noted above. Concordance between ECG-based HRV metrics and PPG-based HRV metrics is indeed modest (Wong et al., 2012; Yuda et al., 2020). In addition, with physical activity shifts in the position of the sensor due to wrist movement will cause measurement error, especially in ambulatory assessments. Even a change in posture may induce a change in the PPG waveform that can affect the fidelity required for estimating HRV (Schäfer & Vagedes, 2013). Emerging methods in engineering and allied fields attempt to correct PPG signals for motion artifact using co-recorded accelerometry and machine learning. To date, evidence suggests promise for using PPG signals to compute HRV metrics under controlled laboratory recordings, but there is wide variability in accuracy under varying time frames of measurement (Foo et al., 2004; Navalta et al., 2020; Poh, Swenson, & Picard, 2010). Unless practical constraints dictate otherwise the ECG is recommended as the best source of HRV derivation (for more, see Section 7 on HP and HRV recording in the MRI scanner and Section 8 on ambulatory recordings).

### 5.2 | Classes of HRV measures

Three classes of measures are most commonly used to quantify HRV: (a) global descriptive measures to characterize the distribution of HPs (e.g., variance and geometrical shape), (b) periodic patterns with specific frequency components of HP variance that relate to functional processes or physiological mechanisms (e.g., RSA), or (c) quantification of nonlinear HRV metrics, such as entropy or other scale-invariant measures, when HRV is viewed as generated by nonlinear dynamical systems. Numerous measures of HRV in these three classes have been employed or proposed over the past several decades and multiple reviews of these measures are available (Acharya et al., 2006; Allen et al., 2007; Bravi et al., 2011; Sassi et al., 2015; Shaffer & Ginsberg, 2017; Sztajzel, 2004; Task Force of the European Society of Cardiology and the North American Society of Pacing and Electrophysiology, 1996; Xhyheri et al., 2012). Table 1 summarizes the most commonly employed HRV measures derived from the HP time series and Table 2 summarizes those that use both the HP time series and the continuous recording of respiration signals. We discuss a selection of these in more detail below, organized by the three classes of measures. Many freely available tools in Matlab, R or Python are available to extract HRV measures from raw ECG (and respiration) signals or from the raw HP time series, with preprocessing options for automated or interactive artifact detection and rejection (Bartels et al., 2017; Blechert et al., 2016; Kaufmann et al., 2011; Nabian et al., 2018; Rodriguez-Linares et al., 2014; Schulz et al., 2009; Vest et al., 2017, 2018), see for example QRSTool by John Allen’s group (Allen et al., 2007, Appendix), HRVtool by Vollmer (2019); PhysioNet Cardiovascular Signal Toolbox by Vest and colleagues (Vest et al., 2017, 2018), RHRV by Martínez et al. (2017), and NeuroKit2 by Makowski et al. (2021) and Pham et al. (2021).

#### 5.2.1 | Global descriptive measures

Global descriptive measures of HRV express variability by conventional variance measures, by the geometric properties of histograms, by sequential differencing or by metrics derived from graphical representations like a Poincaré plot. As early as 1996, the Task Force of The European Society of Cardiology and The North American Society of Pacing and Electrophysiology (1996) recommended descriptive measures for general use in clinical studies. Two that have been used extensively since are the standard deviation of all normal heart periods (SDNN) and the root mean square of the successive beat differences (RMSSD). Both can be used to estimate short-term variability and variability across longer periods, traditionally 24 h. These measures have two major advantages over the more sophisticated measures reviewed below: they are easy to compute and are relatively less sensitive to incorrectly handled artifacts or true rhythm deviations like ectopic beats. However, they may not optimally describe the periodic patterning that characterizes HRV, although RMSSD does reflect short-term periodicities, is abolished by vagal blockade (Penttila et al., 2001) and can be highly correlated with other measures of short-term variability, such as HF HRV, under certain conditions (for a discussion of limits on these correlations see Berntson et al., 2005).

#### 5.2.2 | Measures of periodic patterns

Measures reflecting periodic patterns treat the HP time series as auto-correlated and generated by a series of oscillators with different center frequencies (that can themselves change over time). As described before, the two main sources of variation in HP are respiration (Anrep et al., 1936a, 1936b; Hirsch & Bishop, 1981; Katona & Jih, 1975) and the slow (~0.1 Hz) oscillation in blood pressure (Sayers, 1973). The periodicity in HP that is linked to respiration and blood pressure can be captured by time-domain measures like the peak-valley measure that quantifies RSA from the HP time series and a co-recorded respiration signal (Grossman & Wientjes, 1986; Houtveen et al., 2002), or the sequence method that uses the HP time series and a co-recorded beat-to-beat blood pressure to detect the action of the baroreflex (Omboni et al., 1993; Steptoe & Vogele, 1990), although not necessarily its sensitivity (Diaz & Taylor, 2006; Lipman et al., 2003). Cross-spectra between the HP time series and the respiration or blood pressure signals also can be examined (de Boer et al., 1985a, 1985b; Mulder, 1992; Porges et al., 1980; Robbe et al., 1987). The periodic patterns linked to respiration and blood pressure can alternatively be derived from just the HP time series by frequency-domain analyses such as autoregressive (AR) functions or spectral analyses based on the Fourier transformation (Akselrod et al., 1981; Parati et al., 1990).

The psychophysiological significance of RSA has led to a predominance of modeling HRV as a periodic pattern in our field (Grossman & Svebak, 1987; Mulder et al., 1991; Porges, 1995, 2003), whereas in cardiology, descriptive statistical measures of HRV are more widely used, owing to their long track record in predicting disease outcomes (Chung & Morgan, 1969; Kleiger et al., 1987; Wolf et al., 1978). Below, we describe methods that serve to selectively parse respiratory frequency oscillations from other periodic components, that is, time- and frequency-domain methods used to estimate RSA.

##### Peak-to-valley RSA

In this method, RSA is quantified breath-by-breath, using a peak-to-valley measure of HP fluctuation within each respiratory cycle (Grossman, van Beek, & Wientjes, 1990; Katona & Jih, 1975). The peak-valley RSA (pvRSA) statistic represents the difference between the longest HP during expiration and the shortest HP during inspiration, and was first implemented by Grossman and colleagues (Grossman & Svebak, 1987; Grossman, van Beek, & Wientjes, 1990). Inspiratory and expiratory intervals are extended forward by 750 ms to accommodate phase shifts in the effect of the respiratory phase change on the HP. Mean RSA is computed across all breaths with a clear respiratory-linked shortest and longest HP. Breaths that show no phase-related acceleration or deceleration are assigned an RSA value of zero. This is accomplished by requiring that both the shortest HP during inspiration is preceded by a longer HP, and the longest HP during expiration is preceded by a shorter HP. Thus, an RSA score of zero would be assigned in cases with, for example, a linear decrease in HP over an entire respiratory cycle, which would yield the shortest inspiratory HP, but no longest expiratory HP (de Geus et al., 1995; Goedhart et al., 2007; Grossman et al., 1991; Grossman & Svebak, 1987). By its nature, peak-valley RSA acts as a dynamic time-domain filter, with the ongoing respiratory frequency as its center frequency. Byrne and Porges (1993) provide a detailed description of the method and filtering characteristics that can lead to mis-estimation in the presence of trends in HP over the measurement period and phase shifts. Formulae in the article can assist in determining and addressing possible mis-estimations in cases where there is not a sufficiently stable HP over the measurement period.

##### Moving polynomial filter

An additional time-domain approach that has been employed frequently in the literature to measure RSA is the adaptive polynomial filter method of Porges and Bohrer (Lewis et al., 2012; Porges, 1986; Porges & Bohrer, 1990). This uses a patented algorithm to yield the $\hat{V}$ metric of RSA—commonly called the V-hat metric. This approach is a hybrid time-domain method (but with frequency-domain band-pass filtering). Like spectral techniques, this technique enables derivation of HRV within specified frequency bands. Briefly, in this method one first derives a HP time series and then applies a moving polynomial filter to remove slow trends in the data. A specified band-pass filter is then applied to the data to remove variance outside the target frequency band. The statistical variance of the residual data is derived as an estimate of HRV within the target frequency band, usually over several short time epochs (e.g., 30–60 s) to produce multiple estimates that can be averaged. A log-transform is an inherent step in the computation of the $\hat{V}$ metric, because without the transform, this metric typically has a non-normal distribution.

##### Frequency analyses

Periodicities in a HP time series also can be captured by frequency-domain methods using the efficient implementation of the Fourier Transform, the Fast Fourier Transform (FFT; Akselrod et al., 1981), trigonometric regressive spectral analysis (Rudiger et al., 1999), normalized Lomb-Scargle periodograms (Clifford & Tarassenko, 2005), or parametric autoregressive (AR) modeling (Cerutti et al., 2001). These analyses generate a power density spectrum (PSD) that plots the mean amplitudes of the periodic oscillations in the HP at different frequencies across the frequency range from 0 to 0.5 Hz. The plot thus shows quantitatively the relative contribution of oscillations in different HP frequency ranges to the total variance (power) in the HP time series.

Spectral power in the typical respiratory frequency range of 0.12 to 0.40 Hz can be used to specifically index respiration-HP coupling. HF HRV is most often derived from FFT or AR approaches, and their main difference is the way in which the data are viewed. The FFT analysis assumes that the time series contains only deterministic components, whereas the AR analysis treats data as a composite of deterministic and stochastic components. The spectrum computed with the FFT is derived from all the data regardless of how well they fit a model based on peaks in the spectral distribution. With AR techniques, the time-domain data are used to identify a best-fit model from which multiple peaks and the final spectrum are derived. AR techniques concentrate on the more significant peaks, attempting to exclude “noise,” whereas FFT-based techniques include all data. Thus, the FFT approach could be considered a descriptive method and the AR approach would be more consistent with a stochastic or statistical approach. In practice, this distinction is blurred by the common application of smoothing algorithms or windowing to stabilize variance estimates from FFT analyses. FFT does not require specification and testing of an optimal model (order), which is an advantage. AR has the advantage of not suffering from spectral leakage due to windowing and production of a smoother spectral density plot with better identification of central frequencies of the HF band. In practice, however, the methods usually lead to essentially equivalent results (Hayano et al., 1991; Parati et al., 1995).

##### Cross-spectral analysis

Other methods to describe HRV have been employed. The quantification of RSA can be viewed as a spectral analysis problem associated with a bivariate time series consisting of HP data and an appropriate measure of respiratory activity. Early work by Berger and Saul and colleagues gives the first and most descriptive account of cross-spectral analysis as a method for evaluating the coupling between respiration and HP variability in humans (Berger et al., 1989; Saul et al., 1989; Saul et al., 1991). This approach determines the frequency-dependent transfer of respiratory modulations to HP rhythms, with the magnitude and phase angle associated with the transfer function providing insights into the dynamics of respiratory-cardiac coupling. Porges et al. (Porges & Bohrer, 1990; Porges et al., 1980) introduced the metric of $C_{w}$ or weighted coherence, which is the proportion of variance shared by the HP and respiration signals in the respiratory frequency range (0.12–0.42 Hz, in the original formulation by Porges et al., 1980). While crucial for the early understanding of the transfer function between respiration and HP and its modulation by respiratory parameters, the basic principles of cross-spectral analysis have been employed most extensively in research on the cardiac baroreflex (Di Rienzo et al., 2009). They are, however, re-utilized in more recent quantifications of RSA (Cui et al., 2020).

#### 5.2.3 | Measures from nonlinear dynamics

Complex interactions between the pre-autonomic cell groups in the brain and the peripheral autonomic effectors generate hemodynamic, respiratory, and endocrine effects as well as complex interactions among these effects that together appear to operate as a nonlinear dynamical system. Such systems can be described by measures of short-term complexity (e.g., detrended fluctuation (DFA) analysis short-term α1 exponent), entropy (e.g., sample entropy), and long-range correlation and fractal scaling (e.g., 1/f power law, DFA analysis long-term α2 exponent). For data representation, Poincaré plots, low-dimension attractor plots, singular value decomposition, and attractor trajectories have been used (Acharya et al., 2006; Bravi et al., 2011; Sassi et al., 2015; Voss et al., 2009). These measures have not often been used in psychophysiology but are increasingly used as risk markers in cardiology. For example, predictive clinical value has been found for beat-to-beat dynamics characterized by HP turbulence after ventricular premature complexes (Bauer & Schmidt, 2003; Schmidt et al., 1999) and the deceleration capacity of HP (Bauer et al., 2006).

A chaotic system is described by a set of state variables (that can be represented by coordinates in an n-dimensional space) and a dynamical rule that specifies the future values of all state variables. The collection of coordinates at any time is the state space or the phase space if connected in a smooth manifold. HP variability tends to be attracted to a subset of the phase space called an *attractor*. The parameters that have been used to describe and quantify chaotic properties of HRV include different measures of predictability and fractal dimension (Acharya et al., 2006; Bravi et al., 2011; Sassi et al., 2015). In contrast to most of the linear time- or frequency-domain HRV measures, deterministic and chaotic nonlinear HRV measures require having substantial knowledge of underlying fractal dynamics. Understanding and applying chaos theory and implementing critical steps, like phase space construction and choosing embedding dimensions, are non-trivial. Also, a clear correspondence of these parameters to aspects of the physiological regulatory systems that generate the observed HP and HRV remains to be established.

### 5.3 | Derivation of an appropriate time series for HRV analysis

Successive HPs entail a series of data points spaced unevenly in time, whereas spectral methods assume that the data are sampled at equal time intervals. The HP time series (or beat-by-beat HR series) can be analyzed by spectral methods because the data points are equidistantly spaced in the beat series (de Boer et al., 1985b; Rompelman et al., 1982). The direct submission of a HP series to spectral analyses is not optimal for most purposes, however, because the abscissa of the spectral plot is expressed in units of cycles/beat rather than of cycles/second. A problem with this approach is that beats vary in duration, and results expressed in cycles/beat may not be related in a simple way to events or processes (such as respiration) expressed in seconds. However, this is less of a problem with another time series that is also measured on a beat-to-beat basis such as systolic blood pressure (de Boer et al., 1985a, 1985b).

For the analysis of HRV from HP time series, the generally preferred approach is to derive an equal-interval time series so that data points are equally spaced in time. A variety of methods have been used to derive such an equidistant time series by directly sampling the discontinuous HP time-series signal at regular intervals or by transforming it to a continuous signal, for example, by creating a low-pass filtered event series that reflects the HP-generating process (de Boer et al., 1985a). When the former is used, sharp transitions should be avoided by using by spline interpolation or the weighted average HP of the beats that fall within the sample interval (Berntson et al., 1995; See Box 1 for computation of weighted HP). An important consideration is the selection of an optimal sample interval for this newly derived time series. By definition, a HP represents a single sample per beat and has a maximum frequency content of half the sampling frequency or 0.5 cycles/beat. For an average HR of 60 bpm (1000 ms HP or 1 Hz), the maximum frequency content is 0.5 Hz. To avoid aliasing, the required sampling rate must be at least twice as high (i.e., 1 Hz), but a sampling rate of 4 Hz is generally preferred, which also allows processing of the shorter HPs of newborns or adults during exercise.

#### 5.3.1 | Appropriate choice of epoch lengths

An important consideration in HRV analysis is the standardization of recording lengths used for comparisons between studies and for within-study experimental contrasts. HRV tends to increase with the length of the analyzed epoch (Saul, Albrecht, et al., 1988) because the chance of nonstationarity will increase, so total variance and to some extent that of its spectral components is not a well-defined measure in the absence of information about the duration of the recording. A second important consideration is that the recording duration must allow enough cycles of the oscillation of interest to be included to calculate a reliable estimate of the phenomena. The Task Force Guidelines (Task Force, 1996) recommended, and we concur, that the recording duration be at least 10 times the wavelength of the lower frequency bound of the investigated frequency band. On this basis, a recording of approximately 30 s (in typical adults with a HP ≤ 1000 ms) or 1 min (at a HP > 1000 ms) is needed to assess the HF component, and approximately 2 min are needed to assess the LF component. With children having an HP ≤ 500 ms, 15 s epochs may be sufficient (although requirements for the LF are the same as for adults). When replicate samples are desired, they could be obtained over successive epochs or over discrete trials separated in time. In either case, analyses of multiple, short recording epochs (30 s-2 min) would minimize the likelihood of nonstationarities and permit evaluation of trial-to-trial variance and potential systematic changes over trials. In the absence of systematic change, the aggregate results of this approach should be comparable to metrics computed over longer epochs (e.g., 10 min). In fact, recordings of RMSSD as short as 10 s and SDNN as short as 30 s, when aggregated across multiple epochs during a resting baseline, can closely approximate results with longer duration recordings (Munoz et al., 2015).

#### 5.3.2 | Appropriate definition of the HF frequency band

To measure RSA from spectral methods purely based on a HP time series, it is important to define an appropriate frequency band that captures the variation in HP linked to respiration when using frequency analyses or the adaptive polynomial filter method. This task is non-trivial as the standard HF band between 0.12 and 0.40 Hz (corresponding to respiration rates between 7.2 and 24 breaths per minute) will hamper interpretation of the HF HRV metric as reflecting the RSA phenomenon in slower or rapidly breathing individuals. Of note, this issue is not limited to frequency-domain methods. If RMSSD, for example, is used with participants showing slow breathing rates, the results may reflect both sympathetic and parasympathetic influences, thereby defeating the effort to isolate an index of parasympathetic activity.

For infants and children, who breathe faster than adults, the reverse problem arises in that the upper bound of 0.4 Hz of the HF-HRV measure also may filter out respiratory effects on the HP. For this reason, it has been suggested to adjust the respiration range (and thus HF-HRV band) for this population to 0.24–1.04 Hz (Shader et al., 2018). Finally, “cardiac aliasing” (Witte et al., 1988) may occur if the HR is not at least twice the respiration rate, because it would fail to meet the minimal Nyquist frequency for sampling (minimum of two HPs per respiratory cycle). Fortunately, this problem (which is not correctable by changing the upper end of the respiratory frequency band) appears to be limited to human neonates with higher respiration rates (e.g., >60 breaths per min) and longer HPs (e.g., >500 ms) and to non-human animal studies.

The recommended way to detect the presence and extent of problems in defining the appropriate HF frequency band, is to measure respiratory behavior by co-recording a respiration signal. Measurement of respiration permits identification and exclusion of participants with respiratory rates outside the selected HF variability band. Epochs containing substantial respiratory activity outside the HF band can be removed from analyses. The co-recorded respiration signal is also important for detecting artifacts due to respiratory maneuvers such as yawning, coughs, or speech. If no respiration signal is available, the respiratory peak in the power spectrum of the HP time series can be examined to see that it occurs within the defined 0.12 to 0.4 Hz range for each participant, although this assumes that the respiratory peak in the power spectrum is focal and visually apparent. Furthermore, respiratory frequency can be estimated from the HP itself, generally to within around one breath/min (Thayer et al., 1996, 2002).

#### 5.3.3 | Stationarity

Methods that extract periodic components of variance over time require that the data be at least weakly stationary and may produce biased results if this assumption is not met (Weber et al., 1992a, 1992b). Strict stationarity requires that the distributional characteristics of a series (including all moments) be invariant over time, whereas weak stationarity requires only that the first and second moments (mean and covariance) are stable across time. Periodic pattern analyses assume that the HP time series shows at least weak stationarity. This assumption applies to both time-based and frequency-based methods (Weber et al., 1992a). Violations of stationarity of mean and variance in HP time series may be quite common in short-term recordings (Grossman, 1992; Porges & Bohrer, 1990; Weber et al., 1992b) and almost certain in long-term recordings.

Nonstationarities in the mean can be dealt with by removing trends based on linear or more complex (e.g., polynomial) models (Litvack et al., 1995; Porges & Byrne, 1992). The application of band-pass filters to isolate the periodicities of interest may further minimize the effects of nonstationarities on the mean (Porges & Byrne, 1992), but at the risk of distorting the data when the pass band excludes a main part of the respiratory frequencies (Litvack et al., 1995). However, nonstationarities of variance, because the target cardiac rhythm itself truly varies over time, cannot be removed in a similar way. A first approach to this problem includes restricting the analysis of multiple short epochs within which reasonable stationarity is attained (Weber et al., 1992a, 1992b). A concern with this approach is that the prevalence of nonstationary segments may result in highly selected samples, which may not be representative of the entire data set (Grossman, 1992). To address this problem, results from nonstationary epochs can be compared with those that are stationary. If the results are comparable, it may be possible to “rescue” data from nonstationary epochs. Another approach is the use of methods to quantify HRV that are less sensitive to nonstationarity (e.g., peak-valley approach, Grossman, 1992) or those explicitly designed to characterize nonstationary signals, including modified Wigner–Ville distributions (Martin & Flandrin, 1985), moving periodograms, or wavelet transforms (Houtveen & Molenaar, 2001). These are conceptually more complex approaches and often require more intense data preparation and inspection.

In summary, nonstationarities in HP data may be common and can bias estimates of HRV, especially when they are more prevalent in one experimental condition or behavioral (e.g., ambulatory) context than in another comparison condition because this difference would confound the results. Various approaches of incremental complexity from selection of stationary epochs only, to removing polynomial trends, to approaches explicitly designed to characterize nonstationary signals (like wavelets) can reduce confounding by nonstationarity, but also introduce new problems. Under many circumstances, even large violations of stationarity may not seriously affect the most common uses of LF HRV and HF HRV to warrant the effort (Grossman, 1992; Houtveen & Molenaar, 2001). If (non)stationarity is not explicitly addressed in the analytic approach, we recommend to at least report indicators of the degree of (non) stationarity in the data (for stationarity tests, see Bendat & Piersol, 1966; Weber et al., 1992a, 1992b).

#### 5.3.4 | Impact of experiment design on HRV measures

A specific concern for short-term HRV recordings in a laboratory setting is that periodic patterns in HP in the LF and HF ranges could be induced by the experiment itself, for example, by repetitive or oscillatory time-varying experimental stimuli or events (e.g., task stimuli or responses) that evoke phasic HP changes that are in the LF or HF HRV bands. If a paradigm entails repeated experimental events, the repetition rate should be well outside the frequency band of interest for HRV measures. Even so, caution should be exercised because stimulus or response-driven cardiac reactions are rarely sinusoidal and may introduce broadband spectral noise or harmonics that lie well outside the basic event repetition rate.

### 5.4 | Selection of the appropriate HRV measures

Power spectral (FFT, AR modeling), the adaptive polynomial filter method, RMSSD, and the peak-to-valley statistic are among the most commonly used approaches currently available to analyze periodic components of HRV. Each of these methods can provide valid estimates of respiratory-linked components of HRV when the target rhythm is sinusoidal, there are no exogenous (e.g., experimental) sources of bias, and the data are reasonably stationary. The relative advantages and limitations of these methods will strongly depend on research design and instrumentation but also on the research purpose (e.g., using a measure that has a predictable response to a stressor, or understanding the fundamental biology of the generation of HP rhythms). This means that no universal recommendations for a specific method can be given.

Even so, a sensible criterion to favor one HRV method over another would be to consider its relative ability to track the HP response to parasympathetic blockade. One study has conducted such a head-to-head comparison between various HRV measures and did so, understandably, in a small sample (Lewis et al., 2012). This, unfortunately, raises statistical power concerns and complicates the interpretation of results. For example, Lewis et al. (2012) suggested that the adaptive polynomial filter (Porges-Bohrer) measure was superior to the pvRSA measure because the former was correlated 0.59 with the change in HP during glycopyrrolate infusion, and the latter only at 0.35. However, pvRSA was left untransformed whereas the $\hat{V}$ metric was ln-transformed, which makes for an unbalanced comparison. Furthermore, a simple Fisher Z transformation test on these correlations yields a *Z* of −1.036 (*p* = .15), suggesting the difference was not significant. Additional and larger studies are needed to detect which of the HRV measures used best captures the HP response to parasympathetic blockade, and whether that generalizes to different conditions and populations. We identify this as an important knowledge gap.

Direct comparisons of the estimates from different methods have yielded generally comparable rank-order results with cross-method correlations of > 0.80 (de Geus et al., 1995; Friedman et al., 2002; Grossman, van Beek, & Wientjes, 1990; Hayano et al., 1991; Hill et al., 2009; Lewis et al., 2012; Penttila et al., 2001), even in prolonged recordings (Goedhart et al., 2007; Kleiger et al., 1991). Comparison of these methods to nonlinear methods is more rare, but generally also produces comparable results (Voss et al., 2009). Despite this strong empirical convergence, researchers may favor one of these methods over others based on merits such as ease of derivation, reduced sensitivity to ectopic beats (due to less dependency on the quality of automated/interactive correction of deviant beats), the ability to generate more detailed time-frequency information (e.g., wavelets, Wigner–Ville distributions), and insensitivity to violations of the stationarity assumption or the assumption that the signals derive from linear sinusoidal processes. Weighing of practical and theoretical considerations will typically influence the selection of methods. The peak-to-valley statistic is limited to RSA and, like weighted coherence or other cross-spectral techniques, requires co-recording of the respiration signal. However, taking respiratory behavior into account in the analytic approach is optimal when interpreting RSA to reflect parasympathetic activity. Approaches like wavelet or nonlinear dynamical methods are mathematically more complex and correct interpretation requires greater than average signal-analytic skills. Ease of derivation and reduced sensitivity to artifacts clearly favor descriptive measures like RMSSD and SDNN or even FFT-based measures.

Several other often-cited merits of one method over another are a cause of some concern, as they seem to lack rigorous biological plausibility and empirical grounding in validation studies. For example, the Task Force Guidelines (1996) are often quoted as indicating that “Frequency–domain methods should be preferred to the time–domain methods when investigating short-term recordings (page 364).” However, no strong rationale was provided in these guidelines and many time-domain alternatives for short-term recordings like the peak-valley statistic or the adaptive polynomial filter method were not considered. We do not see a specific overarching reason to generally prefer frequency-domain over time-domain measures for short-term recordings, except when precise specification of frequency bands is necessary or desirable.

Another misunderstanding is a common assumption that detrending and filtering the HP time series and logarithmic transformation of RSA measures are properties of a specific method. Although they are inherent in the adaptive polynomial filter method, trend removal can be employed by any HRV analysis method and likewise any HRV measure can be transformed to conform more closely to a statistically normal distribution. When applied under comparable conditions (trend removal, band-pass filtering, natural log transformations, and aggregation across short analytical epochs), highly correlated estimates of RSA were obtained with FFT analyses and the adaptive polynomial filter method for both actual and simulated data, although the moving polynomial method may yield distortions at selected frequencies (Litvack et al., 1995). In a similar vein, Lewis et al. (2012) compared reliability within and across sessions of the peak-to-valley method and moving polynomial filter method under baseline conditions as well as their differential sensitivity to respiration and vagal blockade. The sensitivity of peak-valley and spectral-based measures to changes in respiration or mean HP were notable in the absence of logarithmic transformation and baseline adjustments for these measures. Thus, there are no firm grounds for concluding that one method is superior to another under all conditions. The general recommendation is that, regardless of the measure employed, it is always prudent to make appropriate adjustments for distributional characteristics, remove general trends in the overall HP, and attend to the potential effect of changes in respiration.

### 5.5 | Minimizing confounding of HRV measures by respiratory behavior

Particularly (but not exclusively) when the interest is in an HRV measure of RSA, taking respiration into account is highly recommended given the importance of changes in respiratory rate and tidal volume as critical determinants of changes in RSA independent of changes in cardiac parasympathetic activity. Three approaches have been used to minimize confounding of HRV measures by respiration: (1) manipulation of respiration by paced breathing, (2) verification of the (reasonable) assumption that experimental conditions do not alter respiratory parameters appreciably, and (3) statistical adjustment of changes in RSA measures for parallel changes in respiratory behavior across experimental conditions.

In controlled laboratory settings, respiration can be experimentally controlled by pacing it to external cues for rate and/or depth (Brown et al., 1993; Grossman et al., 1991; Grossman, Stemmler, & Meinhardt, 1990). Changes in tonic cardiac parasympathetic activity have been shown to closely correspond to within-subject changes in mean HP and RSA during behavioral tasks when respiratory parameters were controlled experimentally (Grossman et al., 1991; Grossman & Kollai, 1993). This relationship appears to hold even when alterations of cardiac parasympathetic activity are modest (Grossman, Stemmler, & Meinhardt, 1990). A disadvantage of paced breathing is that the respiratory control requires appreciable effort, particularly when moved outside of the subject’s spontaneous breathing range or when combining it with experimental manipulations that require considerable mental effort, thereby effectively resulting in a divided attention or divided effort paradigm. The extra effort induced by the “paced breathing task” could exert a direct effect on cardiac parasympathetic activity. Separating the effects of the experimental manipulation of interest and the paced breathing task on RSA may be problematic. When studying RSA outside of a laboratory setting, for example, during ambulatory recording, paced breathing will not be feasible.

Even without experimental control over changes in respiratory measures, it may still be possible to interpret within-subject changes in RSA as mainly reflecting changes in cardiac parasympathetic activity when the experimental conditions do not appreciably alter respiratory parameters. The effects of some manipulations on respiration rate are in the range of two to three breaths per minute (Houtveen et al., 2002). As can be gauged from the transfer functions such changes have only modest effects on RSA (Berger et al., 1989), particularly above respiration rates of nine breaths per minute (0.15 Hz). Indeed, adjustment of HRV for respiration has been found to barely change experimental effects on HRV (Gianaros et al., 2003; Houtveen et al., 2002). However, when experimental manipulations induce moderate to severe stress, the typical respiratory pattern will change to shallow breathing characterized by higher frequency, lower tidal volume, relative hypocapnia, and a predominant thoracic mode (Grossman, 1983; Grossman & Taylor, 2007). Even more significant changes can occur when experimental manipulations include overt speech, calming breathing as an active relaxation technique (as in mindfulness or meditation), or static and dynamic exercise activities.

If experimental conditions *do* result in meaningful changes in respiratory parameters, interpretation of associated changes in RSA as reflecting changes in cardiac parasympathetic activity is problematic (Grossman & Taylor, 2007; Ritz, 2009, 2023). Various approaches can be used to statistically adjust changes in RSA for parallel changes in respiratory parameters. One is to remove respiratory contributions to RSA in each condition by regressing out respiratory effects on RSA and using the resulting residualized scores in a repeated measures analysis (Berntson, Cacioppo, Binkley, Uchino, et al., 1994; Grossman et al., 1991). This, however, assumes that between-subject relationships between RSA and respiration translate to within-subject relationships, which may not be true (Ritz & Dahme, 2006). Inspired by the early work on the transfer function (Berger et al., 1989; Grossman et al., 1991; Saul et al., 1989), Ritz and Dahme (2006) introduced a comprehensive strategy to compute a respiration-controlled RSA that removes the variance in changes in RSA caused by changes in respiratory rate and depth (see also Schulz et al., 2009). This method exploits the near-linear relationship between the RSA/tidal volume (RSA/VT) quotient and the respiratory frequency (which they express as average duration of a breath, Ttot) in the predominant range of natural breathing. In a baseline calibration procedure, the within-subject regression of the quotient of peak-valley RSA and VT (RSA/VT) on Ttot is established for each breath across three to four epochs of paced breathing (e.g., 7.5, 5, and 3.3 s, equals 8, 12, and 18 breaths/min). The regression parameters are then used to estimate RSA/VT for each breath of a given Ttot in a subsequent experiment. The difference between the observed RSA/VT at each breath and the RSA/VT that was predicted by the baseline regression coefficients is the respiration-adjusted RSA measure (RSA/VT_c_). Because changes in RSA/VT_c_ closely correlated with changes in beta-blocked heart rate, it captures the experimentally induced changes in parasympathetic activity (Grossman et al., 1991; Grossman & Kollai, 1993).

Another way to allow for individual differences in the regression of RSA on respiratory changes is to use a multilevel approach to RSA adjustment, with mean-centered respiratory variables as time-varying covariates at Level 1 and participant characteristics (e.g., age, sex, but also baseline respiration frequency) modeled as Level 2 factors. This method can use multiple respiratory parameters as predictors, allowing for only respiratory rate to be used when no tidal volume is available (Cui et al., 2015), or measures of central respiratory drive (i.e., end-tidal partial pressure of CO_2_) to be added if they are measured (Houtveen et al., 2002). A test of an interaction effect between changes in inspiration and participant characteristics on experimentally induced RSA changes would show presence and extent of the moderation of the within-subject RSA-r espiration relationship by participant characteristics.

The regression-based adjustment approaches are conservative and run the risk of removing actual experimental effects on RSA that correlate with respiratory changes (Miller & Chapman, 2001), although this is not always observed in practice (Grossman, Stemmler, & Meinhardt, 1990). The most straightforward interpretation would be possible when a significant experimental effect is observed both with analysis of the raw unadjusted data and after adjustment for possible respiratory contributions. In this case, it would be possible to rule out the contribution of respiratory effects to the experimental effects.

The above adjustment procedures assume a unidirectional causal model, in which experimental manipulations alter respiration, more or less directly, and these respiratory alterations then produce secondary changes in RSA. However, respiratory-vagal coupling itself may be altered in specific behavioral contexts because descending projections from rostral neurobehavioral substrates modulate brainstem autonomic mechanisms (e.g., Inui et al., 1995; Loewy & Spyer, 1990; Neafsey, 1990). Moreover, stressors, even as modest as mental arithmetic, can modulate the baroreceptor-HP reflex (Ditto & France, 1990; Lawler et al., 1991; Stephenson et al., 1981; Steptoe & Sawada, 1989) and possibly the transfer function between respiration and HP. Because central substrates determine respiratory parameters, covariations between RSA and respiration cannot be assumed to be merely secondary to respiratory effects under all conditions (e.g., Denver et al., 2007). Clearly, this is a complex issue and another knowledge gap that calls for additional research. Of particular relevance would be a systematic examination of the impact of respiratory parameters on RSA amplitude during typical psychological tasks, including laboratory stressors, and under different levels of parasympathetic activity. Comparable issues extend to ambulatory recording contexts where respiration changes across behavioral states. For extended discussions of these issues, see Ritz (2009, 2023).

A suggestion to use the RMSSD has been made as an approach for addressing respiratory confounding on the basis that “RMSSD appears to be less affected by fluctuations in respiration, and may thus be a more robust indicator of vagal influence” (p. 71, Hill et al., 2009). The origin of this idea comes from the landmark blockade study by Penttila et al. (2001) in which they reported on 12 healthy volunteers. Breathing was fixed at 6, 15, or 24 breaths per min with spontaneous tidal volume and at 15 breaths per min with tidal volume at 500 mL. The increase in breathing rate from 15 to 24 bpm decreased HF HRV (*p* < .009), whereas the changes in RMSSD were non-significant. In a similar vein, Schipke et al. (1999) observed minimal changes in RMSSD across a wide range of breathing frequencies relative to the changes observed in HF HRV. As described above (Section 4.6.1), the impacts on HF HRV were as expected; HF HRV was maximal when the breathing frequency was in the HF HRV band, and lower outside it. Although the RMSSD depends on both respiratory rate and depth, their effects are less notable than the respiratory effects on HF HRV (Berntson et al., 2005). Furthermore, in Schipke et al.’s (1999) study, there was no control for possible changes in respiratory depth when breathing was paced at widely different respiratory rates, so the effects of variation in respiratory depth in this study are unknown. When considering whether time-domain measures such as RMSSD are less sensitive to respiration, it is worth recalling that RMSSD is a measure of overall HRV, albeit with some greater inclusion of higher frequency variation, than say a measure like SDNN. Since RMSSD is partially influenced by frequencies outside the typical breathing frequencies, it may be unsurprising that it is somewhat less sensitive to respiration rate manipulations. Because RSA is a phenomenon that reflects phasic parasympathetic activity due to cardiorespiratory coupling, this detracts from the use of RMSSD as an index of RSA (or cardiac parasympathetic activity).

## 6 | PARTICIPANT CHARACTERISTICS THAT INFLUENCE HR AND HRV

A range of factors contribute to individual differences in HR^4^ and HRV. These factors provide a context in which to interpret, accumulate, and compare findings across studies. Commonly studied factors include development and aging, biological sex, race and ethnicity, adiposity and obesity, aerobic fitness, health status, and use of drugs. With exceptions for development and aging influences, findings are not uniform with respect to the precise influence and direction of effects that most of these factors exert on HR and its variability. Owing to the complexity and scope of the literatures bearing on the sources of influence on individual differences in HR and HRV, a forthcoming companion *Report* to the current *Committee Report* will provide expanded coverage and additional reporting recommendations to complement those provided here. Accordingly, we provide only brief treatment of participant characteristics and provide basic reporting recommendations below.

We may ask “what is the difference in HR or HRV over a period of hours (or longer) between two groups of people who differ on some feature (e.g., low stress exposure vs. high stress exposure, depressed vs. non-depressed, or treated vs. untreated)?” When comparing HR or HRV in (groups of) participants that differ in a characteristic of interest, it is important to measure and control for other between-subject variables that can independently alter HR or HRV. For instance, if HR or HRV differs between two groups, for example, depressed versus non-depressed, it is important to both measure and control for potential confounding by age, biological sex, race and ethnicity, polygenetic risk scores for depression with potential pleiotropic effects on HR/HRV, socioeconomic status, adiposity and obesity, aerobic fitness, health status, and use of drugs.

**FIGURE 11:**
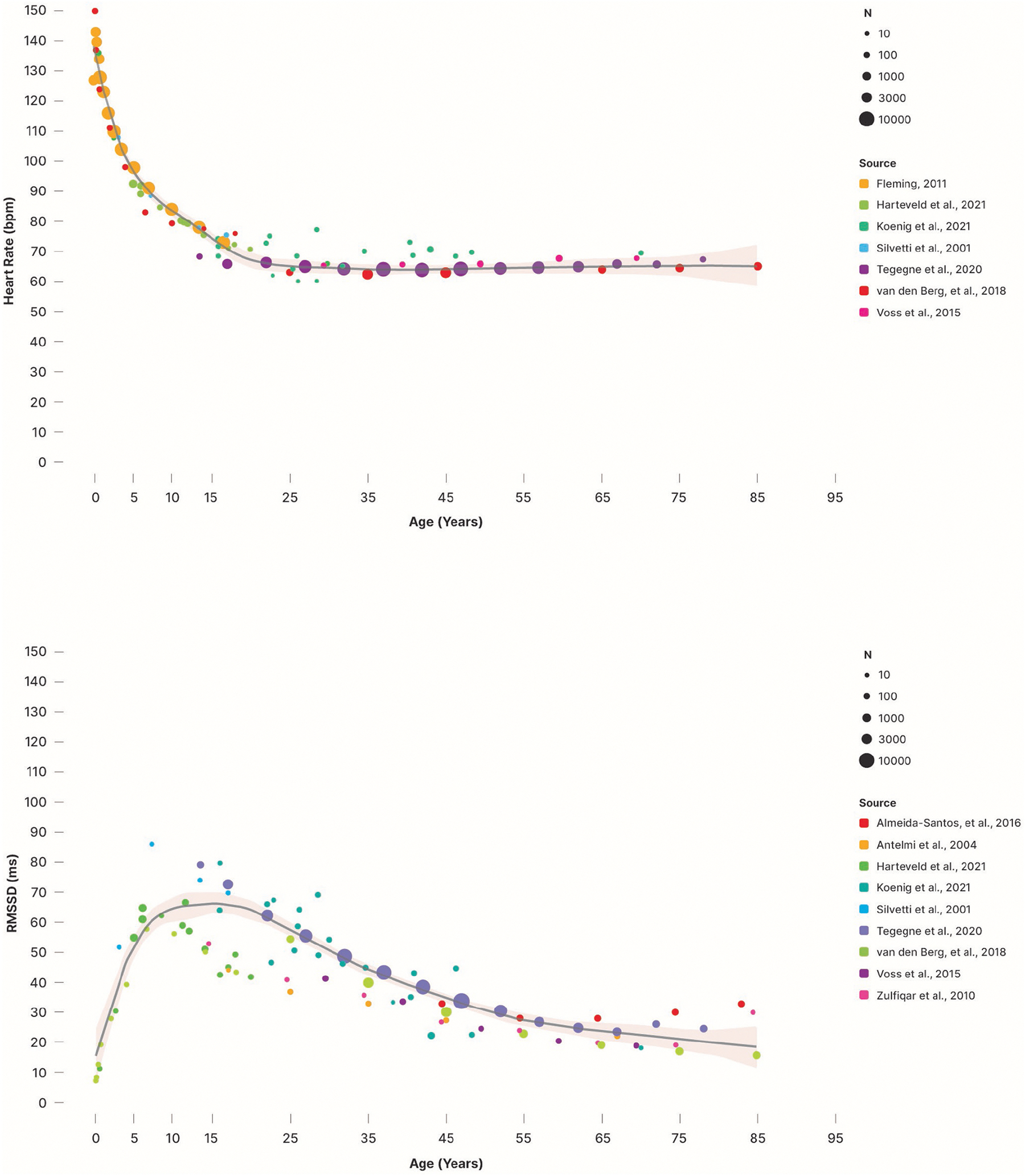
Age effects on resting levels of heart rate (HR) (upper panel) and root mean square of the successive beat differences (RMSSD) (lower panel) from childhood to adulthood. The figure displays data from large (>3500) population-based studies (Fleming et al., 2011; Harteveld et al., 2021; Tegegne et al., 2020; van den Berg, et al., 2018) or studies spanning a large (>70 years) age range (Almeida-Santos et al., 2016; Antelmi et al., 2004; Koenig et al., 2021; Silvetti et al., 2001; Voss et al., 2015; Zulfiqar et al., 2010) measuring resting levels of HR and RMSSD in narrow age bins at different ages. They suggest a complex pattern of maturation in childhood and adolescence for both HR and HRV measures that, for HRV, gives way to a gradual but asymptotic decline in adulthood.

In these regards, chronological age and biological sex are the two predominant participant characteristics that can contribute to interdependent effects on HR and its variability across the lifespan (for reviews, see De Maria et al., 2023; Kerkhof et al., 2018). First, a distinction must be made between the phases of childhood and adolescence, when complex and nonlinear changes in HR and HRV take place related to maturation (Harteveld et al., 2021), and adulthood where HR and HRV show a more linear relationship to chronological age (Peters et al., 2020). Figure 11 depicts the development of HR and RMSSD across the lifespan. In later life and with advancing age, cardiac tissue undergoes myriad structural and functional changes that limit its performance, with many of these changes appearing to differ by sex (Keller & Howlett, 2016). The aged heart, for example, is normatively characterized by increasing atrial dilation, calcification of the aortic valve, thickening of the left ventricle, fibrosis, an increase in fibroblast cells, a decrease in myocytes, enlargement of the remaining myocytes, and a slowing of electrical conduction in cardiac tissue (Kane & Howlett, 2018; Keller & Howlett, 2016). As a result of aging, the pacemaker activity of SA node myocytes declines. These declines stem from both intrinsic structural and electrical changes that occur in individual nodal cells, as well as changes in the responsiveness of these cells to extrinsic (e.g., autonomic) input (Peters et al., 2020). A caveat, however, is that basal resting HR appears to stay relatively stable across adulthood, although there is a linear decrease in the intrinsic HR as measured under dual autonomic blockade, and a decline in the maximum HR that can be attained with maximum aerobic exertion in later life (Peters et al., 2020). The stability of basal HR in adulthood with advancing chronological age in the face of a declining intrinsic HR (estimated at 118 – (0.57 × age in years)) is interpreted as reflecting an age-related decrease in parasympathetic cardiac activity and a commensurate later-life increase in sympathetic cardiac activity (Jose & Collison, 1970; Peters et al., 2020).

A downward trend for the intrinsic and maximum HRs with advancing age appears comparable for men and women (Burke et al., 1996; Peters et al., 2020), despite longstanding and cumulative evidence that women exhibit both higher basal and intrinsic HRs than men overall (Gowd & Thompson, 2012; Larsen & Kadish, 1998). Women may, however, show a possible decline in basal HR compared with men in late life (Smetana & Malik, 2013). That women exhibit higher intrinsic HRs than men has been taken as evidence that autonomic influences do not fully account for manifest sex differences in basal HR (Larsen & Kadish, 1998). Although evidence indicates that women exhibit a faster basal HR than men (Gowd & Thompson, 2012; Kerkhof et al., 2018; Larsen & Kadish, 1998), the developmental timing of when this sex difference emerges may be variable (Larsen & Kadish, 1998; Smetana & Malik, 2013). Finally, sex differences in cardiac electrophysiology and chronotropic activity also can be modified by sex-specific states (e.g., phase of the menstrual cycle, pregnancy, oral contraceptive and hormone therapy usage, menopause; Schmalenberger et al., 2019, 2020; Simon et al., 2021), as well as with exposure to sex steroid hormones (Dart et al., 2002; Mendelsohn & Karas, 2005; Schmalenberger et al., 2019).

Other factors that can influence HR and its variability have been increasingly studied in addition to chronological age and biological sex. These include race and ethnicity (Farrell et al., 2020; Hill et al., 2015; Kemp, Koenig, et al., 2016; Sloan et al., 2008), aerobic fitness and physical activity (Allen et al., 2007; Parker et al., 2010; Sandercock et al., 2005, 2008), features of the metabolic syndrome (Stuckey et al., 2014), and use of certain prescription drugs, such as some classes of antidepressants (Kemp, Fráguas, et al., 2016; Kemp et al., 2010; Licht et al., 2010; for review, see Fiani et al., 2023).

Beyond the factors described above, others that may plausibly exert autonomic and cardiovascular effects and impact HR and its variability over acute or long-term timeframes include tobacco use, particularly smoking; caffeine and alcohol consumption, particularly in time periods immediately preceding or coincident with (e.g., ambulatory) cardiovascular monitoring; and eating and drinking (Allen et al., 2007; Grant et al., 2018; Uijtdehaage et al., 1992). Existing evidence on their systematic effects is lacking, however, including evidence on their timeframes of action. We nonetheless recommend standardizing abstinence protocols (e.g., from tobacco, caffeine, alcohol, drugs, food, and beverages) across participants for laboratory studies, reporting participant characteristics with respect to habitual use of tobacco products and other drugs, and recording and reporting consumption behaviors for ambulatory studies, including the occurrence and extent of consumption both before and during monitoring periods in field settings. In addition, we recommend avoiding correction for participant characteristics by covariance approaches whenever feasible. Although covariance approaches can suppress confounding they are often unjustified on conceptual grounds and may actually “remove” variance-of-interest in primary dependent measures or outcome variables. Where possible, conducting and reporting stratified analyses or tests for effect moderation by sample-level factors is to be preferred over covariance analysis.

In contrast to the robust within-subject relationship between changes in respiration rate and or volume and changes in HRV measures, the between-subject effects of differences in respiratory behavior on HRV levels is less clear. A number of studies in healthy participants have reported no associations between respiration rate and HRV at rest (Ben Lamine et al., 2004; Denver et al., 2007; Goedhart et al., 2007; Quintana, Elstad, et al., 2016) or a small (<0.3) association only (at least for a non-linear dynamical HRV measure; Snieder et al., 2007). However, some between-group comparisons may still benefit from adjustment because the link between resting respiration rate and HRV may be stronger in some patient groups (Quintana, Elstad, et al., 2016) or during nighttime recordings (Kupper et al., 2005). Adjustment can be done using a classical covariate approach (e.g., see Section 5.5) or can be integrated into structural equation models testing the effects of participant characteristics of interest on HR and HRV simultaneously (de Geus et al., 2019).

## 7 | MEASURING HP AND HRV CONCURRENT WITH NEUROIMAGING IN A BRAIN SCANNER

Monitoring peripheral physiology—including ECG, PPG, respiratory, and blood pressure signals—during human functional neuroimaging provides a basis to examine brain-to-body and body-to-brain interactions across a range of behavioral states that are relevant to understanding both efferent and afferent features of cardiovascular and cardiac autonomic phenomena. Monitoring peripheral physiology also affords opportunities to improve the signal-to-noise ratio in some forms of brain-imaging data, where pulsatile (cardiac and respiratory) movement of the brain physically distorts imaging signal quality. The simultaneous monitoring of peripheral physiology during functional neuroimaging, however, can pose major technical challenges and safety risks. In most psychophysiological applications, human neuroimaging is often conducted by functional magnetic resonance imaging (fMRI). Less often used methods include positron emission tomography (PET), functional near-infrared spectroscopy (fNIRS), and magnetoencephalography (MEG). Compared with the latter imaging modalities, fMRI poses the most complex technical barriers and creates the greatest potential for danger to participants, operators, and equipment when combined with certain physiological monitoring methods. These barriers and dangers have been comprehensively reviewed for the neuroimaging and psychophysiological communities (Gray, Minati, et al., 2009; Mulcahy et al., 2019).

In brief, safety risks in the fMRI environment can be created by exposing recording instruments and their sensors and components to magnetic fields, magnetically induced forces, voltages, and heating currents created by time-varying energy changes from radiofrequency waves. For these reasons, peripheral physiological recording instrumentation must be MR-compatible, and care must be taken in the positioning and placement of recording sensors on the body to avoid burning the skin and stimulating nerves. In addition to these safety issues, signal analysis issues are complex. In the fMRI environment, bioelectrical potentials, such as the ECG, and other common signals of interest are susceptible to artifacts from numerous sources, especially imaging gradients and radiofrequency pulses that can be more than 1000 times larger in amplitude than peripheral physiological signals of interest. Such imaging-related artifacts can be removed from physiological (e.g., ECG) time series to some extent with appropriate collection and analytic approaches (Gray, Minati, et al., 2009; Kasper et al., 2017).

For researchers interested in deriving physiological time-series data to estimate HPs and derive HRV metrics concurrent with fMRI, arguably the safest and least artifact-prone monitoring method is PPG. The PPG signal can be easily obtained from a finger or toe during fMRI. However, the very low temperature of the MRI scanning room can cause vasoconstriction and hence loss or dampening of the PPG signal. Placing blankets over PPG recording sites can help to minimize these effects. Also, during fMRI, participants are often asked to hold a “squeeze ball” in one hand to alert the operator if there is an emergency, while at the same time, the other hand may be used to operate a device (e.g., response box) for task responding. When both hands are in use, movement artifacts will thus likely result from positioning a PPG sensor on a finger of either hand, and hence, the suggestion to consider placing the PPG sensor on a toe. Lastly, if brachial blood pressure is obtained simultaneously with a PPG recording from a finger, then cuff inflation will largely eliminate the downstream PPG signal for the duration of vessel occlusion and thus disrupt any derived time-series data for estimating HP and HRV metrics. In general, it is not advisable to position a PPG sensor on an earlobe due both to poor signal quality at the ear lobe and due to proximity to the magnet surrounding the head. As in laboratory monitoring and as noted in prior sections, deriving HRV metrics from PPG signals is not without problems.

As previously noted, it is equally important to collect respiration when collecting measures of HP and HRV within the scanner. The currently most feasible measures include use of an inflatable cuff on a torso belt and remote sensing of pressure change in the cuff. As with other measures, over time technological advances should reduce the difficulty of assessing peripheral physiological measures within the scanner. Notwithstanding these issues, concurrent monitoring of ECG, PPG, and respiratory activity during imaging is feasible, but technically more challenging than when these measures are obtained outside the scanning environment.

## 8 | AMBULATORY MEASUREMENT OF HP AND HRV

In the above, we have mostly considered HP and HRV metrics as recorded under well-controlled conditions in a laboratory. For some questions, however, the advantages of a controlled but artificial laboratory setting may not outweigh its disadvantages. Examples of this can be found in stress research, where the psychological and physiological processes induced by laboratory conditions may be a poor reflection of the complex dynamics or intensity of real-world stressor exposures and responses. For ethical reasons, laboratory stressors are often not sufficiently intense or prolonged, and thus do not result in the full array of physiological responses that occur with real-world stressors (e.g., Busscher et al., 2015). As a result, laboratory stressors also may fail to reveal slower counter-regulatory responses as well as allostatic adaptations that occur on a time scale of days, weeks or years (Vrijkotte et al., 2000, 2004). Laboratory studies also preclude examination of events that may have the most important clinical or functional relevance such as job-related strain, marital conflict, care of a dependent, or even restful sleep. Not surprisingly, longer term 24-h recordings in natural settings have been shown to have higher predictive validity for future disease outcomes than brief assessments in artificial laboratory or clinic settings for both blood pressure (Hansen et al., 2006; Mallion et al., 1999; Niiranen et al., 2010; Palatini et al., 2004; Pickering & Devereux, 1987; Verdecchia, 2000; Ward et al., 2012) and HRV (Bodapati et al., 2017; Kleiger et al., 1987).

Better ecological validity by way of real-world, ambulatory psychophysiological recordings has become increasingly feasible thanks to portable, lightweight, sturdy, and relatively inexpensive biosensors and data-logging devices for noninvasive ambulatory assessment of physiological measures (de Geus & Gevonden, 2023; Peake et al., 2018). The most common ambulatory measure is HP, but increasingly, researchers are also estimating the independent activity of the sympathetic and parasympathetic branches of the autonomic nervous system. For example, to estimate parasympathetic nervous system activity (Carnevali et al., 2018; Verkuil et al., 2016), HF HRV and RMSSD have been derived from ambulatory ECG or PPG recordings (but for cautions in using PPG-based HRV measures, see Section 8.1.2).

### 8.1 | Ambulatory ECG and PPG

#### 8.1.1 | Ambulatory ECG

Ambulatory ECG recording has a long history due to the early adoption of ambulatory measurement in cardiology. “Holter monitoring” named after Dr. Norman Holter, who pioneered this technique, is used routinely for the early detection of cardiac conduction and rhythm disturbances and for timely management and prevention of sudden death (Corday, 1991). The Holter monitor continuously records a standard nine-electrode (V1-V6) set of clinical ECG channels over a 24-h recording and stores these to a recording device worn on a belt, the hip, or on a cord around the neck. For many psychophysiological applications, multiple ECG channels often are not needed because HP and HRV can be extracted with reasonable fidelity from any two skin contacts. These contacts can be created using Ag/AgCl electrodes on the torso, often in a modified Lead II configuration (de Geus et al., 1995; Hoemann et al., 2020), or via contact strips in chest bands (e.g., as used by the Polar and Movisens systems; Brown et al., 2020; Navalta et al., 2020), or in patches (e.g., as used in iRhythm’s Zio or Cardea’s Cardea-Solo patches; Lobodzinski, 2013; Sheridan et al., 2021). The latter patch-based devices are primarily designed to detect cardiac arrhythmias for clinical use and are becoming increasingly common. Several provide an ECG signal obtained from a small form-factor device to which offline (or on-board) machine learning algorithms for fast, efficient detection of potentially dangerous cardiac rhythm abnormalities can be applied (Murat et al., 2020; Neha et al., 2021b; Oudkerk Pool et al., 2021; Sahoo et al., 2020). These smaller, wearable devices are typically optimized for user comfort and unobtrusiveness, and ease of use for clinicians and targeted at the clinical cardiology market, but also can be useful for research when the researcher has access to the raw, non-preprocessed data obtained with a sufficiently high sampling rate.

Ambulatory ECG recordings are more prone to technical failures than lab-based recording because no experimenter is present to monitor or correct technical issues such as loose electrodes, and because the participant’s free movement itself can be a major source of artifact, even with stably attached electrodes. This creates an even larger demand on quality control assessment of the ECG signal, and on artifact detection and correction than is typically required in a laboratory setting. Artifact detection and correction needs to be at least partly automated, since the sheer volume of data can make it virtually impossible to visually inspect all data. The advantage, however, is that a substantial number of problematic beats can be removed and still leave sufficient data for average HP and HR calculation. Large amounts of data loss due to artifact rejection will negatively impact HRV measures which rely on an unperturbed time series (Alcantara et al., 2020).

Despite the many factors outside of the researcher’s control, 24-h duration recordings of ECG-based HP and HRV (RMSSD, HF HRV, pvRSA), when separately tested across waking and sleep, show high test–retest reliability over consecutive days (Bigger et al., 1992; Bjelakovic et al., 2017; Sztajzel et al., 2008; Vrijkotte et al., 2001). Even across much longer periods of months or years, there is good temporal stability of ambulatory pvRSA, HF HRV, and RMSSD (Goedhart et al., 2007; Harteveld et al., 2021; Pitzalis et al., 1996).

#### 8.1.2 | Ambulatory PPG

A disadvantage of continuous skin contact-based ECG recording is that it can be tolerated for a few days to weeks, but is harder to maintain when recordings last multiple weeks or months. Yet only longer ambulatory recordings can provide sufficient time series to establish some associations between longer-term psychological and physiological states within persons. More user-friendly devices requiring less skin contact can be useful when performing prolonged small-sample studies of HP and HRV, or multilevel modeling of larger samples with prolonged idiographically rich time series. The PPG has the major advantage of being minimally invasive and easy to incorporate into wrist-worn devices. As noted above, the HP is most reliably detected using the distance between two R waves in the ECG (see Section 3), but it may be appropriate for certain questions to use the distance between two sequential peaks of the PPG signal. Investigators should weigh the issues related to the less-than-optimal reliability and validity of the PPG-derived HRV versus the greater feasibility/participant acceptance of a PPG recording, allowing for very long recordings.

The use of ECG versus PPG for ambulatory measurement of HP and HRV reflects a prime example of the difficult trade-offs facing today’s researcher when selecting an instrument for their ambulatory psychophysiological research. Research-grade devices generally fare best when it comes to validity, but consumer-grade devices fare better in user acceptance, are typically less invasive, thereby reducing measurement reactivity, and often come at a lower cost. A key feature for researchers to consider is the nature of the measurement desired (e.g., a multi-minute HP mean vs. a precise measure of HF HRV). The nature of the measure (and the question for which the measure is being used) must, in part, dictate the researcher’s choice. Recent guidance on considerations when choosing ambulatory devices for research use can be found in de Geus and Gevonden (2023) and Kleckner et al. (2021).

### 8.2 | Addressing confounders in ambulatory studies

There are two key requirements for ambulatory psychophysiological recording. First, in exchange for the high ecological validity of recording in naturalistic settings we relinquish control over exposure to many of the factors of interest. We can observe but not control stressors (e.g., interpersonal conflict, high cognitive load, low predictability, high background noise), emotional or affective states (e.g., subjective distress, positive and negative affect), physical and social contexts (e.g., features of the home or work such as temperature and humidity) and/or interactions with significant others, colleagues, or strangers. Second, we relinquish control over the many confounders impacting HP and HRV in 24-h recordings, of which the most notable are physical activity, postural change, speaking/not speaking, and the sleep–wake cycle (Grossman et al., 2004; van Lien et al., 2011; Wilhelm & Grossman, 2010).

The solution to these challenges of ambulatory psychophysiological recording is to co-record the features of interest as well as the potential confounders, for example by using ecological momentary assessment (EMA)/experience sampling methods (ESM; e.g., self-report of current social interaction partners; Hoemann et al., 2020; Shiffman et al., 2008; Trull & Ebner-Priemer, 2020) and/or measuring features of the context (e.g., via audio- or videorecording, measurement of physical activity and posture). EMA/ESM uses smartphone-based self-reports and/or passive sensing from wearable sensors (e.g., accelerometry and GPS in fitness trackers and sport watches). Furthermore, smartphones can be used, often in combination with machine learning-based prediction algorithms, to detect the person’s environment or infer the activity in which the person is engaged (e.g., using call logs, sound snippets, and density of Wi-Fi networks; Berlin & Van Laerhoven, 2012; Soares Teles et al., 2017).

#### 8.2.1 | Co-recording of posture and physical activity

As a first step in the analysis, the ambulatory HP and HRV recordings should be adjusted for confounding effects of changes in physical activity and postural change. This can either be done by selecting episodes with fixed posture and low activity levels (e.g., Hoemann et al., 2020; Vrijkotte et al., 2000), or by establishing the transfer function of HP/HRV on the confounders and then adjusting the observed signal for the signal predicted by the confounders. Applied to HR this yields the “additional HR” (Brouwer et al., 2018; Myrtek et al., 1988; Wilhelm et al., 2006), and the “additional” concept can also be extended to HRV (Brown et al., 2020; Verkuil et al., 2016). After stratifying for posture and activity level or after statistically removing the effects of these confounders, the second step is to test for the association between the HP/HRV and the co-recorded behavioral/psychological factors in daily life that are of interest to the researcher. This can be done separately across different classes of posture and physical activity or on the values of HP/HRV adjusted for the effects of posture and physical activity.

#### 8.2.2 | Co-recording of the respiration signal

As outlined above, respiratory behavior can strongly impact HRV measures independent of changes in parasympathetic activity (Eckberg, 2003; Grossman & Taylor, 2007; Houtveen et al., 2002), suggesting that co-recording of respiration is considered a best practice anytime HRV metrics are derived. As discussed above, in controlled laboratory settings, respiratory behavior can either be controlled or measured to ensure that changes in respiratory rate and depth are minimal or are addressed in the analyses. In ambulatory settings, it is not feasible to exert experimental control over these parameters, and the range of variation in respiratory depth and rate are greatly increased due to effects of physical activity, postural changes, and speech (or singing). This makes it important to co-record respiratory behavior in ambulatory studies, particularly when using HRV metrics to assess within-subject changes in cardiac parasympathetic activity over time.

The best validated sensors for continuously measuring frequency and depth of breathing in ambulatory settings either use airflow detection through face masks or nose thermistors, but these are not well-tolerated for prolonged periods and interfere with daily life activities. An alternative is to utilize respiratory inductance plethysmography (Grossman et al., 2010; Kent et al., 2008; Kent et al., 2009) or impedance plethysmography, which can be obtained during impedance cardiography (Ernst et al., 1999; Houtveen et al., 2006). When using the latter technique, there is the advantage that one can also measure various impedance-derived variables such as estimates of pre-ejection period (PEP) and stroke volume, which can be estimated from measures of thoracic impedance. Co-derivation of respiration rate during impedance cardiography has proved to be highly reliable, but estimating respiratory depth is feasible only after posture- and person-specific calibration (Houtveen et al., 2006).

Less burdensome for participants is extracting respiration rate from an ECG or PPG signal. A respiration signal can be re-constructed using changes in the position of the ECG electrodes caused by respiratory-induced chest movements, and based on morphological changes in the ECG, including baseline drift, slope metrics derived between points in the QRS complex, or from R-wave amplitude variation (Varon et al., 2020). Over 100 algorithms have been proposed to estimate respiratory rate (RR) from the ECG or HP time series, and a number of these perform better than the hospital standard of impedance pneumography (Charlton et al., 2016), although their performance in the field remains to be established (Liu et al., 2019). Using spectral decomposition of the HP time-series data obtained from ECG or PPG, the respiration rate also can be estimated using the peak frequency in the HF band. However, the drawback of this approach is that there is often not a focal peak or there may be multiple peaks in the HF band that can complicate estimation of respiratory rate. Moreover, there is no opportunity, with this method, to identify or detect respiratory power outside the prescribed HF frequency band.

## 9 | PHYSIOLOGICAL ORIGIN OF THE HEART RHYTHM

Pacemaker cells of the SA node driving the rhythmic electromechanical activity of the heart are usually localized to the border between the superior vena cava and right atrium, but the location can change with the prevailing HP (Brennan et al., 2020). We use the classical term “SA node” in this report; however, the pacemaker-generating region is more accurately described as a “pace-making complex” of multiple interacting pacemaker regions (Brennan et al., 2020; Papaioannou et al., 2013). Pacemaker cell firing exhibits intrinsic rhythmicity that derives from the coupled action of two “clocks,” a “membrane clock” and a “calcium clock” (MacDonald et al., 2020; Monfredi et al., 2013). The “membrane clock” comprises feedback loops among a set of ionic currents across the SA nodal cell membrane (MacDonald et al., 2020). Activity of the membrane clock couples to a “calcium clock.” The resulting “coupled clock” generates the pacemaker rhythm (Lakatta et al., 2008). Once a threshold potential is reached by spontaneous depolarization across the SA nodal cell membranes, an action potential is generated (see Box 2).

If the HR was dependent solely on the pacemaker rhythm, its basal frequency would be in the range of 70–120 bpm. However, the typical resting HR is much lower than this basal pacemaker frequency, averaging around 65–70 bpm in healthy young to middle-aged adults. The difference between the basal pacemaker frequency and resting HR is caused by the modulation of pacemaker activity by intrinsic and extrinsic factors. Intrinsic factors include physical stretch of the SA node itself, and extrinsic factors include autonomic influences (Wink et al., 2020), and circulating humoral factors (MacDonald et al., 2020). Extrinsic modulation by the PNS is achieved by the vagus nerve, with the source of the efferent vagal chronotropic fibers arising predominantly from the nucleus ambiguus, and to a much lesser extent, the dorsal motor nucleus of the medulla (Gourine et al., 2016). Extrinsic modulation by the SNS is principally achieved by postganglionic neurons whose cell bodies reside in sympathetic chain ganglia abutting the spinal cord and whose actions are relayed via the cardiac nerves (Wink et al., 2020). Additional sympathetic modulation may be achieved neurohumorally via compounds released into systemic circulation by specialized chromaffin cells of the adrenal medulla, which itself is considered a postganglionic sympathetic cell complex. At the heart, axon terminals of the PNS and SNS form synaptic contacts with the pacemaker cells and release acetylcholine (ACh; from the PNS) and norepinephrine (NE; from the SNS) as their primary neurotransmitters. Additionally, firing of the SA node is impacted by the intracardiac ganglionated plexuses, sometimes referred to collectively as the “little brain of the heart” (Armour, 2008); these plexuses also predominantly utilize ACh and the role of these plexuses are an area of active inquiry (Hanna et al., 2021). Finally, electrical activity of the SA node is influenced by co-released factors, including vasoactive intestinal polypeptide (VIP), adenosine triphosphate (ATP), and nitric oxide (NO), among other peptides (MacDonald et al., 2020).

**FIGURE 12:**
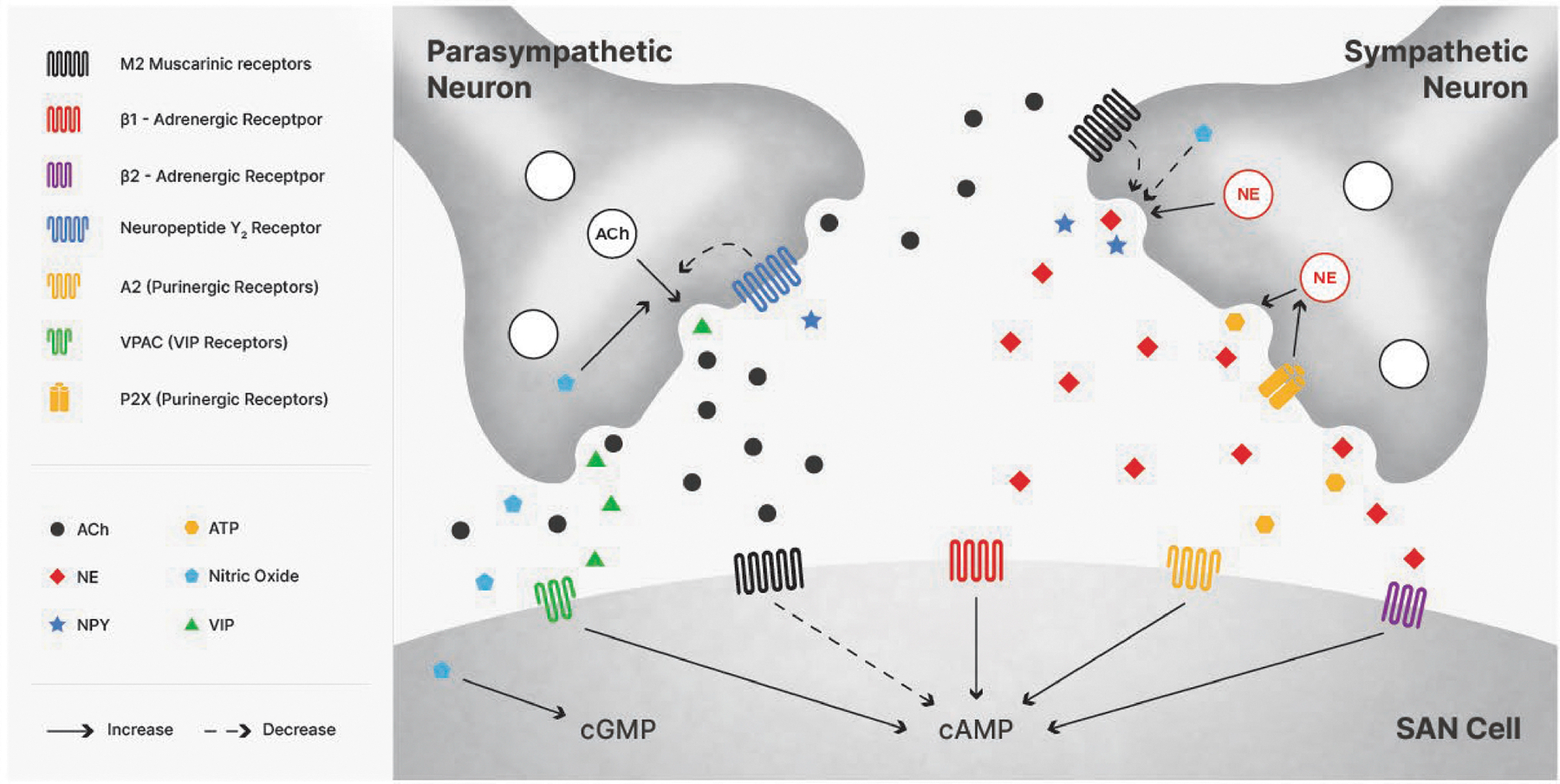
Main and interactive effects of parasympathetic nervous system (PNS) and sympathetic nervous system (SNS) neurons on SA nodal cell activity. Transmitters and receptors diagrammed to show action of these for PNS and SNS effects on sinoatrial (SA) nodal (SAN) activity. We show both positive effects (+) or shortening heart period (HP) and negative effects (−) or lengthening HP. Note there are presynaptic interactions in both parasympathetic and sympathetic neurons, as well as a multiplicity of influences postsynaptically. Figure redrawn from Figure 5 by Fedele & Brand (2020) under the Creative Commons Attribution License.

The effects of parasympathetic and sympathetic activity on the sinus node are not independent. The close vicinity of the parasympathetic and sympathetic synapses and their axo-axonal connections allows for presynaptic neuromodulation prior to downstream effects on the SA node itself. Indeed, as depicted schematically in Figure 12, neurotransmitters released at the parasympathetic axon terminals also exert modulatory effects at sympathetic terminals, and vice versa. Specifically, ACh *inhibits* NE release by muscarinic M2 receptors on adrenergic varicosities.

Similarly, neuropeptide Y (NPY), which is co-released with NE by the SNS, *inhibits* ACh release through activation of NPY receptors on the terminal buttons of vagal (PNS) fibers. Recent work also has found NPY within most neurons of the right atrial (intracardiac) ganglionated plexus (Hanna et al., 2021). Despite these local modulatory effects, the predominant effect of parasympathetic activity is to slow chronotropic action (lengthen HP), whereas that of sympathetic activity is to speed chronotropic action (shorten HP).

### 9.1 | Neurophysiology of ANS modulation of the SA node

Actions of the PNS and SNS on chronotropy (heartbeat timing) arise predominantly by their influence over the time between spontaneous depolarizations, but PNS and SNS actions also impact action potential duration (MacDonald et al., 2020).

#### 9.1.1 | Parasympathetic activity

As depicted in Figures 6 & 12, ACh is released at axon terminals of the vagus nerve and binds to muscarinic M2 receptors. This binding dissociates the inactive G-protein heterotrimer (Gαβγ), which is composed of three subunits (α, β, and γ) and two components, the Gα subunit and a Gβγ component. The Gβγ component interacts with and activates the G-protein-gated inwardly rectifying potassium (GIRK) channel, which is composed of GIRK1 and GIRK4 subunits. A potassium current (I_KACh_) flows across the GIRK channel, such that positively charged potassium ions (K^+^) exit the cell, resulting in membrane hyperpolarization. This hyperpolarization slows depolarization of the pacemaker membrane thereby prolonging the HP. Conversely, Regulator of G-protein Signaling 6 (RGS6) proteins act as a natural brake on GIRK channel activation when RGS6 binds with G-protein β5 to create the RGS6/Gβ5 dimer complex. This complex activates a cascade that causes a Gαi/o subunit and a Gβγ component to rejoin to form an inactive Gαβγ heterotrimer. Additional details are provided in other sources, including Aziz et al. (2018), Mighiu and Heximer (2012), and MacDonald et al. (2020).

#### 9.1.2 | Sympathetic activity

SNS effects occur when post-ganglionically released NE binds to the adrenergic β1- (and β2-) receptors, which causes adenylyl-cyclase to catalyze cyclic adenosine monophosphate (cAMP) production from adenosine 5′-triphosphate (ATP). cAMP-dependent protein kinases then activate L-type Ca^2+^ channels that hasten depolarization of the pacemaker membrane to shorten the HP. The adenylyl/cAMP signaling pathway constitutes yet another source of interaction between parasympathetic and sympathetic effects, here achieved by exerting opposing effects on the I_f_ current through “funny” channels. Whereas the “stimulatory” pathway enabled by the Gαs subunit accelerates I_f_ diastolic depolarization, the “inhibitory” pathway—enabled by either the Gαi or Gαo subunits—counters this acceleration by decelerating the I_f_-mediated diastolic depolarization. Therefore, the funny channel passes K^+^, Na^+^, and Ca^2+^ ions to yield a net inward I_f_ current, which plays a key role in the generation of the diastolic depolarization. Additional details about the signaling pathways that control SA nodal activity can be found in Herring et al. (2019), Behar et al. (2016), and MacDonald et al. (2020).

##### Autonomic contributions to cardiac timing

The responsivity of the heart to autonomic neural stimulation at different frequencies has been described for both PNS (i.e., vagal) and SNS systems using both steady-state and dynamic stimulation, as well as pharmacological blockades. For example, Parker et al. (1984) demonstrated in dogs that the steady-state increase in HP to vagal stimulation at frequencies between 1 and 30 Hz was almost perfectly linear, findings that have been shown to generalize across many mammalian species (Berger, 1987; Berntson et al., 1992; Rosenblueth & Simeone, 1934; for reviews, see Berntson et al., 1995; de Geus et al., 2019; Eckberg & Sleight, 1992). Parallel studies of SNS stimulation have revealed somewhat less linear effects on steady-state decreases in HP, with diminishing effects at frequencies above 1.5–2 Hz and saturation (i.e., a ceiling effect) at higher frequencies in dogs and cats (Berger, 1987; Levy & Zieske, 1969; Rosenblueth & Simeone, 1934).

Although autonomic nerve stimulation offers some promise for clarifying chronotropic effects of PNS and SNS inputs to the heart, there are caveats. For one, the vagus nerve carries both parasympathetic afferent fibers, as well as parasympathetic and sympathetic efferent fibers (Jayaprakash et al., 2023; Kawagishi et al., 2008; Nielsen et al., 1969). This neuroanatomical arrangement greatly complicates inferences about the effects of vagal efferent activity from vagal stimulation in intact organisms. However, recent developments in recording from vagus nerve fibers in awake humans could enable more precise examination of vagal effects on the heart (Ottaviani et al., 2020; Patros et al., 2022).

The most widely used technique for understanding autonomic contributions to the timing of cardiac events (chronotropy) is the selective blockade of parasympathetic and sympathetic actions on the heart with pharmacological agents. The change in functional status (or response) of an organ after selective autonomic blockade via the administration of antagonists provides one index of the contribution of an autonomic branch to target organ activity (e.g., HP). Thus, the change in mean HP after parasympathetic blockade with atropine often has been used as a criterion index of parasympathetic effects on cardiac chronotropy. Moreover, corresponding sympathetic effects can be similarly calculated via pharmacological blockade (Berntson, Cacioppo, & Quigley, 1994). The blockade approach has been especially useful in elucidating autonomic contributions to different frequency components of HRV (Akselrod et al., 1985; Cacioppo et al., 1994; Grossman & Kollai, 1993; Katona & Jih, 1975; Koh et al., 1994; Kromenacker et al., 2018; Pagani et al., 1986; Pomeranz et al., 1985; Saul et al., 1991).

##### Humoral contributions to cardiac timing

In addition to direct neural innervation of the heart, the SNS also modulates the HP indirectly by adrenomedullary catecholamines (norepinephrine and epinephrine) in the bloodstream, partly by neural spillover and partly by direct hormonal release from the adrenal medulla. Catecholamines can diffuse from the circulation to impact synaptic sinoatrial membranes and bind to adrenergic β-receptors. There is no humoral effect of circulating ACh on sinoatrial muscarinic M2 receptors because this neurotransmitter is cleaved by acetylcholinesterase in the synaptic cleft and does not reach the bloodstream. Norepinephrine is rapidly catabolized in the synapse, but some overflow occurs. Measurement of the latter in plasma has been used to estimate sympathetic activity, although adrenomedullary contributions are still primary (Esler et al., 1985; Tank & Wong, 2011).

## 10 | SUMMARY RECOMMENDATIONS FOR MEASUREMENT AND REPORTING

In several sections, most notably in Section 9, the Committee focused in some detail on the complex biology of the heart and its control, because we felt a solid understanding of this biology is essential for the interpretation of HP and HRV in psychophysiological studies, regardless of the measures and approaches adopted. Owing to its complex biology and determinants, HP and HRV have no one-to-one mapping onto any psychological state, and cannot be used to invariably index states, processes, or individual difference factors across variable contexts. Following an adage long treasured by members of the Society, that inferring psychological significance from physiological signals is very challenging (Cacioppo & Tassinary, 1990), the adoption of an overall stance of careful inference and restraint when interpreting HP and HRV should therefore be encouraged.

This does not distract from the value that these metrics have and will continue to provide to our field. The intent of the checklists in this closing section is to guide authors in the reporting of key methodological details and the characteristics of the samples under study. In these checklists (Tables 3, 4, 5, and 6), we refer the reader to the sections of this report that provide background on the items in the checklists. We do not advise that these guidelines for study design or measure/method selections should be rigidly applied. This is especially true when technological and analytical developments rapidly outdate even the most recent publications. Nonetheless, it is important to provide recommendations for precise and complete reporting on the methods used to derive HP and HRV measures, both for replication and re-use of data, for example, in meta-analyses. As a general rule, the explanation of these methods should allow a competent external researcher to reproduce the analysis solely from the manuscript. In shaping our recommendations for reporting, we build on guidelines offered in other recommended methodological reviews (e.g., Allen et al., 2007; Laborde et al., 2017; Nelson et al., 2020; Quintana, Alvares, & Heathers, 2016).

## Figures and Tables

**TABLE 1:** Heart rate variable (HRV) measures that are derived from the heart period (HP) time series.

HRV measures	Description	Remarks	Recording duration^a^
Descriptive methods			
SDNN (ms)	Standard deviation of Normal-to-Normal interval time series (NB: NN interval equals heart period)	The total variance of HRV increases with the length of the recording used to compute SDNN. In a 24-h period 30–40% of SDNN can be attributed to the wake/sleep transition (Kleiger et al., 2005)	SHORT + LONG
SDANN (ms)	Standard deviation of average heart period calculated in consecutive periods, usually of 5 min	Indexes changes in heart periods due to processes longer than 5 min	LONG
SDNN index (ms)	24 h mean of the standard deviation of heart periods over consecutive periods of, usually, 5 min	Approximates total power in spectral analysis	LONG
RMSSD (ms)	Square root of mean squared differences of successive heart periods	Computing successive differences in heart periods effectively applies a high-pass filter, with gain function 4sin^2^ (π*fHP*). At a HP of 930 ms, the RMSSD is thus suppressed ~4-fold more when caused by the HRV component of 0.1 Hz than by the HRV component of 0.3 Hz. RMSSD is therefore mostly caused by RSA (Berntson et al., 2005). For more on the relative impact of respiratory and blood pressure variation on the overall HRV, see de Boer et al. (1985b). RMSSD can be approximated by the mean absolute value of difference (MSD) between successive heart periods (Allen et al., 2007)	SHORT
NN50 count	Number of interval differences of successive heart periods of which the absolute value is greater than 50 ms	A closely related alternative is the pNN50 proportion (%) derived by dividing NN50 by the total number of heart periods	SHORT + LONG
HRV triangular index	A heart period density distribution, D, is constructed which assigns the number (on the Y axis) of equally long heart periods to each value of their lengths (in ms, on the X axis). The maximum *N*_max_ is D(X), where X is the length of the most frequent heart period. The HRV triangular index is obtained by dividing the area integral of D by the maximum Y, approximated by the value: (total number of heart periods)/*N*_max_	Geometric methods are more influenced by the lower than by the higher frequencies and are inappropriate to assess short-term changes in HRV	LONG
TINN	Triangular interpolation of NN also uses the distribution density plot of heart periods and is the baseline width of the triangle that best captures D (by minimizing the difference between the areas of the triangle and D)		LONG
Periodic pattern methods using Frequency decomposition			
Total Power	Total area under the power spectral density (PSD) curve that equals the total variance	Same as SDNN squared	SHORT + LONG
ULF (ms^2^)	Ultralow-frequency component of the PSD, defined as power in the frequency band ≤0.0033 Hz	Power components should always be reported in absolute values of power (ms^2^), although statistical analysis may require a log transformation. LF and HF can additionally be expressed and analyzed in normalized units (n.u.) by dividing each of these power components by the total power minus the (ULF +) VLF components (Pagani et al., 1986)	LONG
VLF (ms^2^)	Very low-frequency component of the PSD, defined as power in the frequency band 0.0053–0.05 Hz		LONG
LF (ms^2^)	Low-frequency component of the PSD, defined as power in the frequency band 0.05–0.12 Hz		SHORT
HF (ms^2^)	High-frequency component of the PSD, defined as power in the frequency band 0.12–0.4 Hz		SHORT
$\hat{V}$	V-hat metric is a hybrid of frequency-method filtering and a time-domain method to extract variance in a specified frequency band	The Porges-Bohrer method (Porges et al., 1980; Porges & Bohrer, 1990) applies an algorithm that extracts HRV even if superimposed on a complex baseline that may include aperiodic and slow periodic processes	SHORT
Nonlinear methods			
Poincaré plot SD1 (ms)	A Poincaré plot (return map) is created by plotting every R-R interval against the prior interval, creating a scatter plot the area of which conforms to an ellipse that represents total HRV. The SD1 is simply the standard deviation perpendicular to the line of identity of the ellipse	Useful for outlier detection, and to visually search for patterns within a time series. SD1 gives similar information as the RMSSD (Ciccone et al., 2017)	SHORT
Poincaré plot SD2 (ms)	SD2 is obtained from the standard deviation of the Poincare plot along the line of identity of the ellipse	SD2 can be completely captured by combining SDNN and Standard deviation of successive differences of the RR intervals (SDSD) (Brennan et al., 2001)	LONG
Sample entropy	For approximate entropy a window of length *m* is run along the signal to generate a set of data vectors of length m. The number of times that the Euclidean distance between all pairs of these vectors is less than a threshold *r* is computed. This is repeated for windows of length *m* + 1, and the logarithm of the ratio of these two numbers is taken. Compared with Approximate entropy (now deprecated) Sample Entropy (SE) excludes self-matches	Sample entropy is just one metric of a much larger class of informational HRV measures to quantify overall complexity by detecting low or high level of repetition of patterns in a signal. These include, for example, conditional entropy, compression entropy, fuzzy entropy, Kullback-Leibler permutation entropy, multiscale entropy, Shannon entropy, Renyi entropy (Bravi et al., 2011)	LONG
Correlation dimension (CD_2_)	Minimum number of variables needed to describe a HP time series in terms of a nonlinear dynamical system	The CD_2_ value will be higher for chaotic data and decreases with lower total variance or increased rhythmicity	LONG
Lyapunov exponent (λ)	The largest λ exponent reflects the sensitivity of a chaotic system to its initial conditions	Based on the divergence of initially nearby trajectories in the phase space	LONG
DFA α1 and α2	Detrended fluctuation analysis (DFA) measures the root mean square fluctuation of an integrated and detrended HP time series in windows of the time series with different length. A log-log plot of the root mean square fluctuation as a function of the window length variation yields the α1 slope from 4–16 beats, and α2 from 16–64 beats	At least 20 min of data are needed to compute fractal scaling components, and a large part of HRV data in healthy individuals may not conform to the requirements of a fractal model (Tan et al., 2009)	LONG
1/f^α^	Power-law exponent α of the PSD in the very low-frequency band (10^−2^ to 10^−4^ Hz) when plotted on a log-log scale	Like DFA α1 and α2 or the Hurst exponent, 1/f scaling reflects the self-similarity properties of HP time series (Kobayashi & Musha, 1982)	LONG

*Note:* Note that this table excludes measures like pvRSA, and HP/SBP and HP/Respiration cross-spectral measures that combine the HP time series with beat-to-beat blood pressure recordings.

a Can be computed on SHORT term (e.g., 5 min) or on LONG term (e.g., >20 min and up to 24 h) recordings.

**TABLE 2:** Heart rate variable (HRV) measures that derive from the combination of the heart period (HP) time series with the continuously recorded respiration signal.

HRV measures	Description	Remarks	Recording duration^a^
Periodic pattern methods based on the Transfer function			
pvRSA (ms)	Difference between the longest HP during expiration, and early inspiration, and the shortest HP during inspiration and early expiration	Peak-valley RSA can be computed breath-to-breath across all breaths with a clear respiratory-related shortest and longest HP (Grossman & Svebak, 1987; Grossman, van Beek, & Wientjes, 1990). Breaths that show no phase-related acceleration or deceleration are assigned an RSA value of zero. Mean RSA is then computed across all breaths in relevant conditions	SHORT + LONG
*C*w	Respiratory modulation of the HP rhythm obtained from the gain in the cross-spectrum	This quantification of RSA uses cross-spectral analysis on a time series of the HP (rendered continuous) and the respiratory signal (Porges et al., 1980; Porges & Bohrer, 1990). *C*_w_ or weighted coherence in the formulation by Porges et al., 1980) is the proportion of variance shared by the HP and respiration signals in a fixed respiratory frequency range (0.12–0.40 Hz). Ideally use of the gain between respiration and HP is restricted to frequencies where the coherence between the two signals exceeds 0.50	SHORT

a Can be computed on SHORT term (e.g., 5 min) or on LONG term (e.g., >20 min and up to 24 h) recordings.

**TABLE 3:** Checklist for reporting on general design and data collection methods.

#	Information to be included in the manuscript	Sections	Completed?
1.	Properties of the data acquisition set-up (i.e., instrumentation, amplifiers, filters, electrode configuration, sampling rate, and software). Authors are encouraged to report ample and transparent details to enable methodological understanding and replication. It is necessary to describe the raw electric or pulsatile signal used to extract HP or HR (e.g., ECG or PPG signals), including hardware specifications (e.g., brand and model), sensor placement (e.g., lead configuration), sampling rate, and real-time or offline filtering properties of the raw signal. *We recommend preferential use of the ECG over the PPG, with electrode placement favoring R-wave detection*, that is, *in a (modified) Lead II placement, and sampling at 1000 Hz. Use of PPG and lower sampling frequencies down to 100 Hz should be restricted to situations that limit feasibility of ECG use,* for example, *prolonged ambulatory recording. This usage should be accompanied by an acknowledgment of the limitations in precision when interpreting results based on, in particular, the HRV metrics.*	3.1 5.1	Y/N
2.	Experimental recording conditions. For laboratory studies, authors should report details on the study context (e.g., whether there was real-time visualization of the raw ECG or vascular signal, whether participants were supine, seated or standing, whether the recording environment was temperature- and noise-controlled, and whether participants were positioned in a way that reduced movement and strain on electrode leads). For each experimental condition, the length of data collection and duration of the period(s) used for the analysis of HR and HRV metrics should be provided. It also recommended to provide information about how the potential overlap between stimulus or response-driven cardiac reactions and the frequency band of HRV metrics used has been avoided or minimized.	5.3	Y/N
3.	Talking, posture change, and movement. Speaking, respiratory maneuvers (e.g., erratic breathing, sneezing, and coughing), postural change, and changes in gross motor activity can substantially alter raw signals and HR/HRV. Researchers should avoid analyzing signals recorded during such confounding events. If speaking is integral to the experimental condition as in public speech stress tasks, the interpretation of HR and HRV metrics should explicitly address the impact of speaking per se. If the psychological state is manipulated in combination with parallel changes in posture or energy expenditure, the interpretation of HR and HRV metrics should explicitly address the impact of orthostatic and exercise maneuvers per se.	4.6 8.2	Y/N
4.	Behavioral and relevant contextual factors. Authors should provide descriptive information as feasible on all possible behavioral factors and states that may have a confounding impact on HR and HRV (e.g., time of day, time since last meal, recent intensive or prolonged physical activity, time since last intake of caffeine, tobacco products, and alcohol).	6	Y/N
5.	Quantitative characteristics of the HP time-series generation. Report peak-picking detection algorithms used on the QRS complex or pulse wave for the HP time-series derivation, whether the detection methods were fully automated or semi-automated, whether visual inspection was employed, whether and to what extent there was any removal of segments and/or manual interactive editing of individual beats or peaks. Details should also be provided regarding all data handling steps and methodology to identify and resolve artifacts in the HP time series (e.g., interpolation), along with how successive beat events were converted to a continuous HP time series (e.g., by the cubic spline method). *We recommend using data preprocessing that includes a validated software solution (or one that you make verifiable by depositing it in an open-source repository like GitHub) that fully automatically corrects the HP time series, but adds a second stage of supervisory interactive visual inspection*.	3.2	Y/N
6.	Specification of editing steps and procedures. To avoid biases introduced by differential extent of deletion or interpolation of artifacts across recording conditions, participants, or participant groups, the number and/ or mean duration of HPs that were deleted or replaced should be reported. Periods of longer (>5 s) data loss should be reported (e.g., providing explanations for loss, including persistent ectopy, movement-induced noise, and equipment failure). Authors are also encouraged to report quantitative information about editing (e.g., percentage of beats deleted and/or corrected) and when substantial editing (>1%) was done, explicitly test their potential contribution to the results.	3.2	Y/N
7.	Timing and units of measurement. Studies may focus on HP/HR over a timeframe with a particular type of activity, for example, baseline, task, recovery. Counts of heartbeats over such periods can be employed with control of or accounting for any differences in period length. Averaging of HP can be done once transformed to a common real-time axis to appropriately compare individuals while accounting for period length. Studies examining brief responses to exactly timed events should transform HP data to real-time data, that is, HP per beat or HR per second. The events then can be accurately represented on this time scale.	2.1 3.2 Box 1	Y/N

**TABLE 4:** Checklist for considerations specific to HRV metrics.

#	Information to be included in the manuscript	Sections	Completed?
1.	Choice for an appropriate HRV metric and analysis method The many metrics and analysis methods discussed all can provide valid information on the autonomic processes underlying HRV when the target rhythm is sinusoidal, there are no exogenous (e.g., experimental) sources of bias, confounding by respiration and gross bodily movement is accounted for, the HP time series are detrended, and the analysis epochs are kept relatively short to avoid large deviation from stationarity. The relative advantages and limitations of different methods will depend on research design and instrumentation but also on the research purpose (e.g., to detect a predictable response to a stressor, or to understand the fundamental biology of the generation of HP rhythms). It is therefore essential that a rationale is given for the methods chosen and how they fit the research question. *We recommend using triangulation across multiple methods and HRV measures. When introducing a new HRV method, adding a thorough comparison and reporting against one or more established or existing methods is mandatory*.	5.3 5.4	Y/N
2.	Spectral analyses. When using frequency-domain or spectral analysis methods, authors should provide details on whether and which methods were used for low-pass or moving-average filtering, resampling, DC component removal (detrending), the number of samples used for the power spectrum calculation, windowing method (e.g., Welch, Hanning, Hamming), data length and overlap, smoothing of the raw periodogram (e.g., by the Daniell kernel), and the frequency bands used. Reporting on autoregressive (AR)-based metrics should include the central frequency for each spectral component (LF and HF), the value of the model order (number of parameters) and statistical evidence for the fit of the model used. *We recommended that the recording duration be at least 10 times the wavelength of the lower frequency bound of the periodic pattern of interest. On this basis, a recording of approximately 30 s (in typical adults with a HP < 1000 ms) or 1 min (at a HP > 1000 ms) is needed to assess the HF HRV component, and approximately 2 min are needed to assess the LF HRV component. In generating equidistant time series from the HP time series, we further recommend a sampling rate of at least 4 Hz.*	5.3	Y/N
3.	For nonlinear analysis (although these guidelines apply broadly to all methods), researchers should attend to interpreting measures in terms of their potential links to the generating physiological mechanisms.	5.2	Y/N
4.	Data transformations. Because the distributional characteristics of HRV estimates may not meet the assumptions of parametric analyses, a natural log transformation of variance estimates is often needed before statistical testing. Further transformations may be done that include a normalization of HRV measures by the mean HP, or by using modeling approaches that account for, for example, respiratory behavior. When change scores or “reactivity” are used, baseline levels are sometimes regressed out using an ANCOVA approach. A final and often used approach with frequency analyses is the use of normalized units to provide an index of relative variance of a frequency band relative to the total variance. All such transformations need to be motivated and described in sufficient detail in the methods section. *We recommend that, when using transformed, normalized or residualized values, to always also include original, untransformed values and variance/range in the descriptive tables (or at least in supplemental materials). This will support between-study comparisons, including those in meta-analyses.*	3.2 5.5	Y/N
5.	Interpreting HRV metrics as reflecting RSA. For measures to be reflective of RSA and interpreted with precision, it is important to use a co-registered respiration signal and to ensure that rhythmically occurring external events that affect heart rate are not overlapping with the respiratory frequency band. If one wishes to specifically infer HRV spectral power in a predefined frequency band as reflecting RSA *without* access to the respiratory signal, it is necessary to note that the respiratory bandwidth may have exceeded the bandwidth used to compute HRV. If a respiration signal is available, epochs containing substantial respiratory activity outside the predefined frequency band can be removed from analyses.	4.5 5.3	Y/N
6.	Interpreting LF HRV as reflecting cardiac sympathetic activity. The LF HRV reflects an unknown mixture of cardiac sympathetic and parasympathetic activity, under most experimental conditions typical in psychophysiology. *Without refuting their potential clinical utility, we recommend not using either LF/HF ratio or the LF HRV as measures of sympathetic activity in psychophysiological research.*	4.3	Y/N
7.	Interpreting RSA as reflecting cardiac parasympathetic activity. A clear distinction should be made in the use of RSA to index within-subject changes in cardiac parasympathetic activity or to index between-subject differences in cardiac parasympathetic control. For both types of designs co-recording of respiration rate (and if possible, also depth) is highly recommended when the intent is to interpret RSA as reflecting experiment/exposure induced changes in parasympathetic activity or individual differences in RSA levels in a (resting) condition to reflect individual differences in parasympathetic activity in that condition. At high levels of parasympathetic activity, a ceiling effect will increasingly dissociate RSA from parasympathetic activity. *We strongly recommend adjustment of RSA for respiratory behavior in within-subject designs, with joint reporting of adjusted and unadjusted results. Between-subject designs may also benefit from reporting respiration-adjusted analyses. Inspection of the between-subject and (in prolonged time series) the within-subject HP by RSA relationships should be used to rule out/detect ceiling effects. We further recommend using the term cardiac parasympathetic control rather than cardiac parasympathetic activity when the focus is on individual differences in RSA*.	4.5 4.6 4.7 5.5	

**TABLE 5:** Checklist for ambulatory and field recording.

#	Information to be included in the manuscript	Sections	Completed?
1.	All recommendations in Tables 3 and 4. Ambulatory devices should meet similar standards and follow all recommendations in Tables 3 and 4, with similar guidance on real-time visualization of the raw signal because it is useful during initial signal checking (many consumer devices permit signal assessment by streaming to a computer, so that signal quality is confirmed before subjects leave the lab). The specific and detailed description of the data recording protocol (e.g., device placement, minimal wear times), and data preprocessing and cleaning procedures, how much signal was lost, how signal loss could be attributed to systematic sources, and the potential impact of correction strategies on the primary research questions. This should be more detailed and complete than for laboratory studies where (referral to) extensive prior work and methodological standards can sometimes reduce the need for such detailed reporting.	Tables 3 and 4	Y/N
2.	Reproducibility. Many (most often PPG-based) ambulatory devices are available that target the consumer market, but do not provide control over sampling frequency or access to the raw signal data. Measures from such devices should also have been validated against established standards for the dependent measures of interest in published peer-reviewed studies that used representative samples, created substantial variance in the target HP/HR or HRV measure (e.g., using repeated conditions including rest, and mental and physical stress with variable intensity). *For research, we recommend avoiding devices for which the company uses proprietary firmware or hardware to generate higher level outcomes but no raw data and for which the technological principles used in device design or data preprocessing are not publicly accessible. We also recommend the usage of devices for which independent validation of their performance for HR and HRV assessment is available, under conditions similar to those of the planned research.*	8.1	Y/N
3.	Estimation of respiratory frequency. Particularly when RSA is used as an index of changes in parasympathetic activity, it is important to co-record respiratory information. When this leads to unacceptable increases in participant burden, respiratory frequency may be estimated from fluctuations in the ECG waveform or from ECG or PPG-derived spectral analyses.	8.2	Y/N
4.	Registration of ongoing events replaces experimental control. We recommend the collection of additional data on potential confounders using ecological momentary assessment and passive sensing (e.g., accelerometry) methods so that behavioral states, physical activity, and other relevant information can be reported on, used to include or exclude periods from analysis, or otherwise account for confounding influences (e.g., eating, postural change, and physical activity).	8.2	Y/N

**TABLE 6:** Checklist for reporting on participant characteristics.

#	Information to be included in the manuscript	Sections	Completed?
1.	Record and report detailed participant characteristics that could be expected to relate to autonomic regulation, including age, sex at birth and gender, race and ethnicity, current psychiatric or medical conditions, physical activity, smoking, and alcohol and drug use. *In addition to sex as a biological variable or sex assigned at birth, we recommend measuring and reporting sociocultural gender or gender identity.* *Because all these factors are likely to be related to autonomic and cardiovascular measures, we recommend standardizing abstinence protocols (e.g., from tobacco, caffeine, alcohol, drugs, food, and beverages) across participants for laboratory studies, and querying and reporting consumption behaviors both before and during ambulatory monitoring periods in field settings.*	6	Y/N
2.	Drugs with ANS effects. Either use as exclusion criteria or systematically record the use of psychoactive drugs (e.g., SSRIs, SNRIs, TCAs), drugs which lower the intrinsic HR (e.g., ivabradine) and drugs with adrenergic and cholinergic effects (e.g., β-blockers; digoxin, atropine, acetylcholinesterase inhibitors, glycopyrrolate) as they will impact HRV measures.	6	Y/N

## Data Availability

Data sharing is not applicable to this article as no data sets were generated or analyzed during the current study.

## BOX 1 The arithmetic versus the weighted mean HP of heartbeats within an epoch of fixed duration

We borrow here an illustration from Graham (1978) showing the difference between an arithmetic mean and weighted mean for an exemplar 60-mile round-trip traveled between points A to B, where speed for the out-bound trip was 60 miles per hour (mph; A to B), and the speed for the return/inbound trip was 30 mph (B to A). Graham (1978) noted that when computing the average speed over the combined trips, 45 mph may seem intuitive, but it is incorrect. Although 45 mph is the arithmetic mean of the speed across the two trips (i.e., speed/trip), the trips differ in duration and thus are not appropriately scaled by time. Because the outbound trip takes 1 h and the return trip 2 h, the average speed over both trips is 40 mph (1 h at 60 mph, and 2 h at 30 mph = [60 + 30 + 30]/3 = 40). Because the 45-mph estimate does not appropriately weight the two trips for their duration, it is a biased estimate of the average speed.

Graham’s caution also applies to calculating mean HP (or mean HR) for an epoch with a fixed time interval (as psychophysiologists often wish to do). If a HP (or HR) estimate over an epoch of time is viewed as a time series; then, the probability of sampling a specific beat is not equal (except in the very rare instance when there is a constant HP), but instead is a function of the duration of the cardiac event. Said differently, longer HPs are more likely to be sampled than shorter HPs. Incorrect estimates for either HR per or HP per will occur if the arithmetic mean is used (i.e., the mean is not appropriately weighted). Weighting for the duration of heart periods is required as illustrated below (redrawn from the example in Stern et al., 2001).

### Duration of concurrent HP at each sample point:

**Table:**

	Second 1	Second 2	Second 3
Sample 1	750 ms	730 ms	745 ms
Sample 2	750 ms	730 ms	745 ms
Sample 3	750 ms	730 ms	775 ms
Sample 4	750 ms	730 ms	775 ms
Sample 5	750 ms	730 ms	775 ms
Sample 6	750 ms	745 ms	775 ms
Sample 7	750 ms	745 ms	775 ms
Sample 8	750 ms	745 ms	775 ms
Sample 9	730 ms	745 ms	775 ms
Sample 10	730 ms	745 ms	775 ms

7460 ms/10 samples.

### Examples:

1. Calculation for a 1-second Weighted HP for Second 1:
  $$\left[750\;ms\;\times \;8\;samples\right)+\;730\;ms\;\times \;2samples]∕10\;samples\;=\;746\;ms\;for\;second\;1$$
2. Calculation for second-by-second response to a stimulus occurring at sample 16 (i.e., in the middle of Second 2):
  **Pre-stimulus second**: $\left[\left(750\;ms\;\times \;2\;samples\right)+\;\left(730\;ms\;\times \;7\;samples\right)+\;\left(745\;ms\;\times \;1\;sample\right)\right]/10samples=735.5\;ms\;for\;pre-stimulus\;second$
  **Post-stimulus second**: [(745ms×6samples)+(775ms×4samples)]∕10samples=757msforpost-stimulussecond
3. Calculation for a 3-s period (e.g., a baseline or average stimulus (or task) response)
  **3-Second Weighted *HP***: [(750×8samples)+(730×7samples)+(745×7samples)+(775ms×8samples)]∕30samples=750.8msfor3-secondaverage
4. **Conversion to Weighted Average HR**: Take the inverse of the weighted average HP and convert to beats/min
  From Example 1 above: *Weighted Average HR for Second 1*:
  $$\begin{array}{c} {\left(\begin{array}{ccccc} 746\;ms & \times & 1\;sec & \times & 1\;min\\ \text{beat} & & 1000\;ms & & 60s \end{array}\right)}^{-1}\\ \;=60,000/746=80.4\;\text{beats}\;/min\;(\text{orbpm}) \end{array}$$

## BOX 2 Generation of the pacemaker sinus rhythm

In an 82-year-old human, pacemaker cells in sinoatrial nodal tissue will have spontaneously generated over 3 billion heartbeats. Most characteristic of pacemaker cells is their diastolic depolarization interval, which is generated by the coupled membrane and calcium clocks. The depolarization interval is powerfully modulated by extrinsic factors, particularly the release of acetylcholine (ACh) by the parasympathetic vagi onto muscarinic M2 receptors and by the release of norepinephrine (NE) by sympathetic motor neurons onto adrenergic β1-receptors. ACh slows depolarization and lowers the maximal diastolic potential after repolarization, whereas NE speeds up depolarization. These basic autonomic influences on the pacemaker potential lead to the well-known observation that increases in the mean activity of the vagal nerves result in increases in the HP, and increases in the mean activity of the sympathetic nerves result in decreases in the HP.

Although the exact mechanisms remain to be elucidated, progress in our understanding of the spontaneous depolarization in pacemaker cells has been made in the past two decades. Briefly, a HP at the level of the pacemaker cells in the SA node can be decomposed into three main phases as shown in Figure 7.

The Figure introduces the key events in pacemaker potential generation while glossing over many other events that are described in detail elsewhere (Bartos et al., 2015; MacDonald et al., 2020; Monfredi et al., 2014). The end of the repolarization phase (nadir of waveform in the figure), is characterized by the conduction of K^+^ currents through I_k_ channels, resulting in a membrane potential of around −60 mV. This opens “funny” channels (I_f_) that conduct slow, inward Na^+^ currents, ultimately leading to spontaneous depolarization. The latter is further supported by a slow decline in the outward movement of K^+^ as the I_k_ channels are closing. T(ransient)-type Ca^++^ channels gradually open and the inward directed Ca^++^ currents further depolarize the cell. At the end of the depolarization, the “funny” Na^+^ currents and Ca^++^ currents through the T-t ype Ca^++^ channels decline, but now there are large spontaneous sub-sarcolemmal Ca^++^ releases from the sarcoplasmic reticulum (SR) via ryanodine receptors (RyR). This input from the calcium clock results in a depolarizing current as 1 Ca^++^ ion is extruded from the cell in exchange for 3 Na^+^ ions through nearby Na^+^-Ca^++^ exchangers. When the membrane depolarizes to around −40 mV, the L(ong-lasting)-type Ca^++^ channels open and the threshold for excitation is crossed, creating an action potential. Unlike action potentials in most cells (e.g., neurons, muscle cells), the depolarizing current is carried into the SA node cell primarily by relatively slow Ca^++^ currents instead of by fast Na^+^ currents. The action potential duration therefore can last up to 400 ms, much longer than in neurons (~1 ms) for example. The action potential causes I_k_ channels to open which triggers repolarization through the outward directed, hyperpolarizing K^+^ current. The L-type Ca^++^ channels also become inactivated and close, which decreases the inward depolarizing Ca^++^ currents. Cytoplasmic Ca^++^ levels are “reset” by both the SR Ca^++^ pump (SERCA) and the sarcolemmal sodium-calcium exchanger (NCX).

The mechanism for action potentials described for SA nodal cells above is similar to that for cells of the atrioventricular (AV) node. The AV node is therefore itself also capable of intrinsic pacemaker activity, possibly acting as an evolutionary backup. Even the branches conducting the action potential through the heart muscle (His, Purkinje fibers) can be pacemakers. However, because the rate of diastolic depolarization is fastest in the SA node, in the intact heart the SA node takes complete precedence in pacemaker activity.

## Footnotes

1 With concurrent sympathetic activation, sizeable and prolonged attenuation of vagal regulation of the heart may be mediated by neuropeptides (e.g., neuropeptide Y and galanin; See Figure 12) that are co-localized with norepinephrine in sympathetic nerve terminals (Potter et al, 1989; Yang et al., 1994). These effects are particularly potent at higher levels of sympathetic activity (Eugster et al., 2022).

2 The LF or near-0.1Hz rhythm also has also been called the Mayer wave, which can either refer to the 10-s rhythm (Mayer, 1876) or sometimes to fluctuations at frequencies not exceeding 0.05Hz (Madwed & Cohen, 1991; Preiss et al., 1975; Preiss & Polosa, 1974).

3 Across the literature, the lower end of the high-frequency HRV (HF HRV) band used varies, with both 0.12Hz and 0.15Hz as common choices. For consistency, throughout this report, we use 0.12Hz.

4 As the literature on individual differences in cardiac function generally refers to HR rather than HP, we have retained the term HR for this section.

## GLOSSARY

Arrhythmia: A deviation from steady rhythmicity in the cardiac cycle.
Artifact: Abnormalities in the ECG or PPG signal.
Baroreflex: A homeostatic mechanism that constrains oscillations in beat-to-beat BP by rapidly adjusting HR, cardiac contractility, and vascular resistance through coordinated and negative-feedback changes in autonomic nervous system activity.
Cardiac aliasing: A phenomenon that occurs when the HR is not at least twice the respiration rate and thus fails to meet the minimal Nyquist frequency for sampling which interferes with estimations of respiratory sinus arrhythmia.
Ectopic beat: Alterations in the cardiac cycle that are otherwise normal resulting in extra or skipped heartbeats.
Einthoven’s triangle: An imaginary equilateral triangle with the heart at its center and formed by vectors that represent the three standard limb leads of the electrocardiogram.
Heart period (HP): The period between any specific voltage deflections in the ECG from two consecutive heartbeats. Synonymous with the term interbeat interval.
Heart rate (HR): The number of cardiac cycles in a 60-s period, expressed in beats per minute. HR is the inverse of heart period.
Heart rate variability (HRV): Variation in the time intervals between consecutive heartbeats.
Interbeat interval: The period between any specific voltage deflections in the ECG from two consecutive heartbeats. Synonymous with the term heart period.
Intrinsic HR Intrinsic HP: The frequency at which the heart beats when all neural and hormonal modulators of cardiac chronotropy are removed (e.g., by pharmacological blockade), reflecting endogenous sinoatrial node activity. The intrinsic HP is the inverse of the intrinsic HR (60,000/intrinsic HR).
Mayer wave: Oscillations in heart period or arterial pressure at frequencies ~0.10 Hz that correspond to baroreceptor activity.
Respiratory frequency band: Also termed the high-frequency range of HRV, the band (0.12– 0.40 Hz) closely corresponds to the typical respiration rate of adult humans (7.2– 24 breaths per minute) and is used to index respiratory sinus arrhythmia in spectral approaches to HRV.
Respiratory sinus arrhythmia: A deviation from steady rhythmicity of the cardiac cycle that corresponds to the respiratory gating of cardiac autonomic regulation.
R–R interval: The period between two consecutive R peaks in the ECG waveform.
R wave: The maximum amplitude or peak in the QRS complex used to derive R–R intervals.
Sampling rate: The average number of values recorded per second from a continuous-t ime signal.
Sinoatrial node (SA node): A specialized structure within the heart that serves as an endogenous pacemaker and initiates the electrical impulses to stimulate cardiac contraction.
Stationary signal: A signal is stationary when the basic signal properties of mean and variance remain constant over time.
Vagal tone: A term used for tonic cardiac parasympathetic activity. The only valid operational definition of “vagal tone” is when it is used to refer to the difference in mean R–R interval between a resting intact baseline and complete vagal blockade. Measures of HRV vary considerably in their correlations with this criterion definition of “vagal tone,” and under many conditions, HRV measures are not at all correlated with this criterion definition. For these reasons, we do not recommend the use of this term outside the context of vagal blockade studies.
Vagus Nerve: Also known as cranial nerve X, it is the primary preganglionic projection of the parasympathetic nervous system. The vagus nerve originates in the nucleus ambiguus and dorsal motor nucleus in the medulla and serves to regulate a myriad of physiological functions including cardiac chronotropy. It also carries vagal afferents; in fact, proportionally, the vagus has more afferent fibers than efferent ones, with the proportion varying by species and by rostro-caudal location within the vagus; (Jänig, 2022; Jayaprakash et al., 2023; Neuhuber & Berthoud, 2022). Thus, the vagus is both a visceromotor and viscerosensory nerve.
